# Absence of spatial genetic structure in common dentex (*Dentex dentex* Linnaeus, 1758) in the Mediterranean Sea as evidenced by nuclear and mitochondrial molecular markers

**DOI:** 10.1371/journal.pone.0203866

**Published:** 2018-09-12

**Authors:** Alexiane Viret, Dimitris Tsaparis, Costas S. Tsigenopoulos, Patrick Berrebi, Andrea Sabatini, Marco Arculeo, Chiheb Fassatoui, Antonios Magoulas, Michel Marengo, Beatriz Morales-Nin, Nathalie Caill-Milly, Eric D. H. Durieux

**Affiliations:** 1 ISEM, Université de Montpellier, CNRS, IRD, EPHE, Montpellier, France; 2 Institute of Marine Biology, Biotechnology and Aquaculture, Hellenic Centre for Marine Research (HCMR), Thalassocosmos, Heraklion, Crete, Greece; 3 University of Cagliari, Biologia Animale ed Ecologia, Cagliari, Italy; 4 Dipartimento di Scienze e Tecnologie Biologiche Chimiche e Farmaceutiche, Università di Palermo, Palermo, Italy; 5 Institut National Agronomique de Tunisie, UR03AGRO1 Écosystèmes & Ressources Aquatiques, Tunisia; 6 UMR 6134 CNRS-UCPP Sciences pour l’Environnement, Université de Corse Pasquale Paoli, Corte, France; 7 UMS 3514, CNRS-UCPP Plateforme marine Stella Mare, Université de Corse Pasquale Paoli, Biguglia, France; 8 Department of Natural Resources, Mediterranean Institute for Advanced Studies (IMEDEA CSIC-UIB), Esporlas, Balearic Islands, Spain; 9 Ifremer, Laboratory Environnement Resources of Arcachon, UFR Sciences and Technics, 1 allée du Parc Montaury, Anglet, France; National Cheng Kung University, TAIWAN

## Abstract

The common dentex, *Dentex dentex*, is a fish species which inhabits marine environments in the Mediterranean and Northeast Atlantic regions. This is an important species from an ecological, economic and conservation perspective, however critical information on its population genetic structure is lacking. Most samples were obtained from the Mediterranean Sea (17 sites) with an emphasis around Corsica (5 sites), plus one Atlantic Ocean site. This provided an opportunity to examine genetic structuring at local and broader scales to provide science based data for the management of fishing stocks in the region. Two mitochondrial regions were examined (D-loop and COI) along with eight microsatellite loci. The COI data was combined with publicly available sequences and demonstrated past misidentification of common dentex. All markers indicated the absence of population genetic structure from the Bay of Biscay to the eastern Mediterranean Sea. Bayesian approaches, as well as the statistical tests performed on the allelic frequencies from microsatellite loci, indicated low differentiation between samples; there was only a slight (p = 0.05) indication of isolation by distance. Common dentex is a marine fish species with a unique panmictic population in the Mediterranean and likely in the Atlantic Ocean as well.

## Introduction

Patterns of geographic genetic structure in marine fishes rely mainly on life histories and environmental drivers over space and time [1, 2, 3]. Compared to terrestrial organisms, marine species, and marine fishes in particular, generally show a high genetic diversity and a weak spatial genetic differentiation [1, 2, 4, 5, 6]. This is mainly attributed the high prevalence of external fertilization and the production of a huge amount of larvae with high dispersive capability [7] associated with the lack of barriers in marine waters which in turn should facilitate high gene flow between remote populations [6]. However, the development and use of polymorphic genetic markers allow for the describing of a certain degree of spatial genetic structure in marine fishes due to specific connectivity in both adults' movement and early life stage dispersal [8, 9, 10]. Knowledge of the genetic structure of exploited populations is therefore essential to conservation and sustainable management of fishery resources [8, 9, 10, 11]. This type of research is especially necessary to identify populations, delimitate stocks, and determine the connectivity and potential resilience within populations.

The Sparidae family (Rafinesque, 1810) belongs to teleost fishes comprising more than 118 species distributed among 35 genera found in all tropical and temperate oceans [12, 13]. These species are of significant economic interest since the majority are exploited by commercial fishing activities; some have also become important for the aquaculture industry, such as the very popular gilthead sea bream (*Sparus aurata*) in the Mediterranean Sea and the Japanese red sea bream (*Pagrus major*) in East Asia. The common dentex, *Dentex dentex* (Linnaeus, 1758), is a sparid living along the Mediterranean, and the Atlantic Ocean coasts from the British Islands to Senegal, and occasionally in the Black Sea [14]. The species lives near the bottom of sea, from a few meters to 200 m depth, preferably on a rocky substrate. Adults are usually solitary and congregate only in the spring for reproduction [15]. In Corsica, fishermen observed that they congregate between 40 and 100 m depth on hard substrate (rocks, wrecks) [14]. The common dentex is gonochoristic, reaching sexual maturity between 2 and 4 years old [16, 17, 18]. It grows to a maximum length of 100 cm and a weight of 13 kg, with a relatively long life span (up to 36 years) [19]. Due to its large size, flesh quality, and high commercial value, the species is of great interest to both artisanal and recreational fisheries [20]. Furthermore, the common dentex is classified by the International Union for the Conservation of Nature (IUCN) as ‘‘vulnerable” in the Red List of Threatened Species. However, little is known about the population genetic structure of the common dentex. Previous studies sampling partly the same populations as in this survey and based either on allozymes and partial sequences of the mitochondrial D-loop (control) region [21] or morphological biometrics [22] have proposed a strong divergence between the Atlantic and Mediterranean populations. In fact, the Strait of Gibraltar is considered a biogeographical breakpoint [23] between the Mediterranean Sea and the Atlantic Ocean both for pelagic/epipelagic and demersal fish species. Species such as the European sea bass *Dicentrarchus labrax*, the common dolphinfish *Coryphaena hippurus* and other sparids like the striped *Lithognathus mormyrus*, and the black sea bream *Spondyliosoma cantharus* show significant genetic differentiation in this area (reflected by high Fst values between populations both in allozymes and mtDNA) [23, 24].

The aim of the present study was to assess the species identification and then the genetic structure and phylogeography of the common dentex by combining a representative population sampling scheme in the Mediterranean Sea and multiple markers. A denser population sampling scheme includes samples already analyzed by Bargelloni et al. [21] and adds new samples covering the Mediterranean Sea from the east (N. Aegean Sea, Greece), to the west (Alicante, Spain), and up to the Bay of Biscay in the Atlantic Ocean (France). First, sequences from the mitochondrial DNA (mtDNA) cytochrome oxidase (COI) locus were used for barcoding purposes to check for any taxonomic inconsistencies when an individual’s species is determined; subsequently, mtDNA D-loop sequences and multi-locus microsatellite genotype data were employed to refine the intra-specific common dentex population genetic structure within the study area. A recent study [25] established that there is no genetic structure around Corsica. To a lesser extent, we aim to define the species structure in the Mediterranean Sea and one site in the Atlantic Ocean.

## Materials and methods

### Sampling

**Fig 1:**
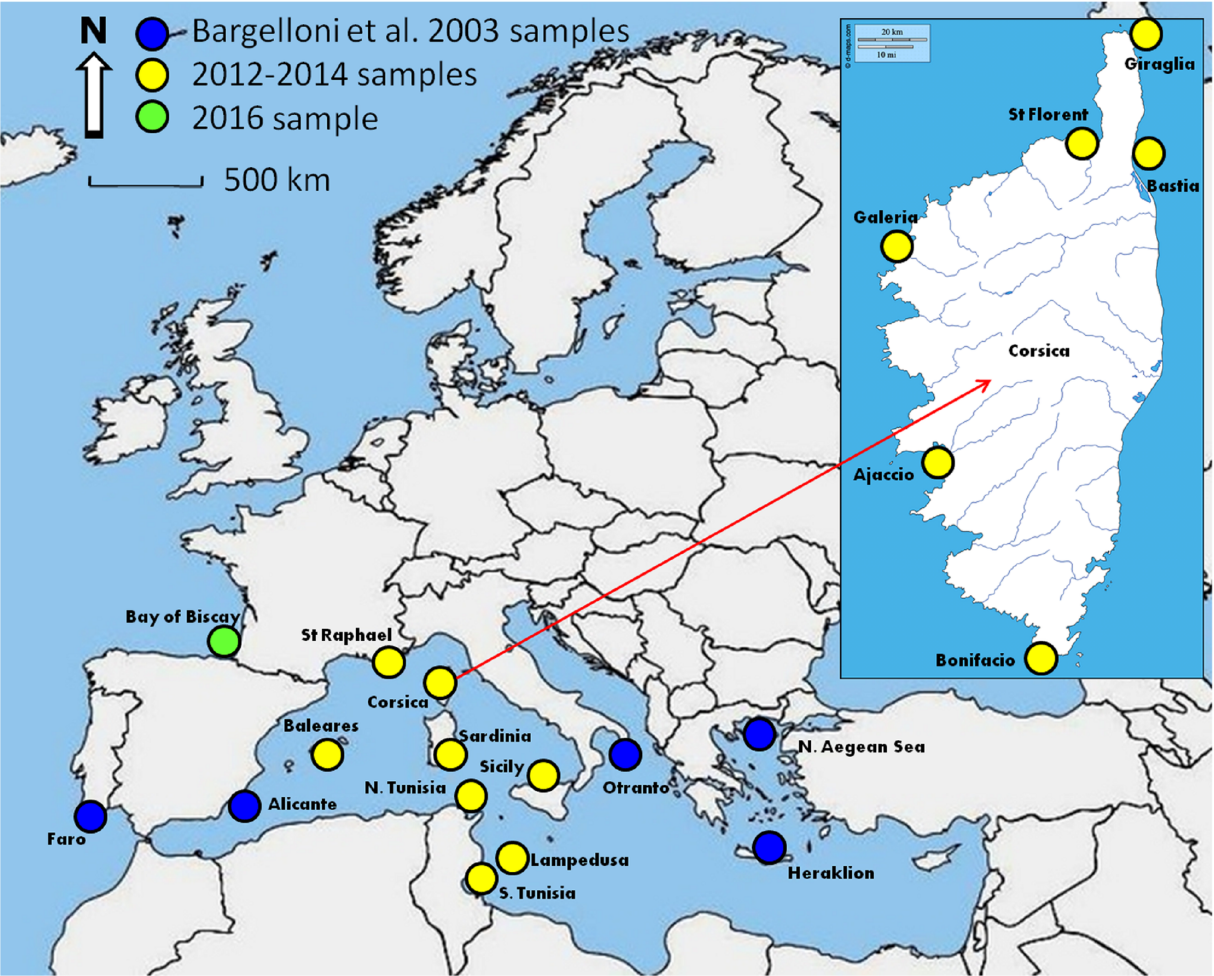
Map of sampling localities of common dentex (*Dentex dentex*) with respective years (blue circle: 2002 Bargelloni [21] samples; yellow circle: 2012–2014 samples; green circle: 2016 sample).

Common dentex tissues (fin clips), sampled in 2002 and kept in ethanol in IMBBC for Bargelloni et al. [21], were re-used in the present study. More recent samples (fin clips and muscle tissues) were obtained by professional fishermen, and fish were caught between the years 2012 and 2016 with longlines, gillnets, trammel nets and trolling. Sampling localities and sizes are reported in Fig 1 and Table 1. These individuals originated from 19 different fishing locations distributed across the Northeast Atlantic Ocean (France and Portugal), the Western basin of the Mediterranean Sea (Spain: two sites, France: seven sites, Sardinia, Sicily and Tunisia) and the Eastern basin of the Mediterranean Sea (Lampedusa, southern Tunisia, the Adriatic Sea, Crete and N. Aegean Sea). At a finer scale, six samples were located all around the Corsican coasts: Bonifacio in the South, Ajaccio, Galeria and Saint Florent in the West, Giraglia in the North and Bastia in the East (Fig 1).

**Table 1:** Collection date and number of analyzed samples (N) for each of the 19 localities and for each of the three markers used.

Locality	Date	N microsatellites	N COI	N D-loop
Heraklion (North Crete, Greece)	2002	24	5	5
North Aegean Sea (Greece)	2002	13	5	6
Otranto (South Adriatic, Italy)	2002	23	5	5
Sicily (Italy)	2014	13	-	-
Lampedusa (Italy)	2013	13	5	5
North Tunisia	2013	14	2	2
South Tunisia	2013	14	1	-
Sardinia (Italy)	2013	23	6	5
Giraglia (Corsica, France)	2012–2013	25	5	5
Bastia (Corsica, France)	2012–2013	-	6	5
St Florent (Corsica, France)	2012	18	5	3
Galeria (Corsica, France)	2012–2013	31	4	3
Ajaccio (Corsica, France)	2012–2013	15	5	5
Bonifacio (Corsica, France)	2012	24	5	5
St Raphael (France)	2013–2014	14	8	6
Baleares (Spain)	2013	19	7	5
Alicante (Spain)	2002	21	5	6
Faro (Portugal)	2002	24	20	-
Bay of Biscay (France)	2016	8	8	8
SUM		**336**	**107**	**79**

### DNA extraction

DNA was extracted from fin clips and muscle tissues. For each sample, a piece of tissue about 2 mm x 4 mm was taken from ethanol-preserved specimens, dried and dipped into 200 μL of Chelex previously heated to 56°C. The DNA extraction was performed according to a Chelex protocol [26] with proteinase K with a final concentration of 0.198 mg. mL-1. The mix was placed overnight in an oven at 56°C and then at 95°C for 5 min. Finally, samples were centrifuged at 3,000 rotations per min for 2 min. The supernatant was diluted 1:20 with sterile water. The samples were then frozen (-20°C) until use.

### MtDNA sequencing analyses

In order to resolve any taxonomic uncertainties concerning either old samples [21] or new samples, we performed a DNA barcoding analysis using the cytochrome c oxidase subunit 1 (COI) mitochondrial gene as a marker for species-level identification [27]. A 612 bp fragment at the 5' end of COI was amplified with primers FishCoxI-F2 (5'-TCGACTAATCATAAAGATATCGGCAC-3') and FishCoxI-R2 (5'-ACTTCAGGGTGACCGAAGAATCAGAA-3') [28]. The 10 μL PCR mix included 20–50 ng of template DNA, 1x Taq buffer, 0.2 μΜ of each primer, 0.2 mM dNTP mix, 1 U of Taq polymerase, 2.5 mM MgCl2 and ultrapure water. The PCR cycling protocol consisted of an initial step of 2 min at 95°C, followed by 35 cycles of 0.5 min at 95°C, 0.5 min at 50°C, and 1 min at 72°C, followed by a final extension step at 72°C for 10 min.

A 444–456 bp long fragment of the D-loop region was also amplified in order to study the mtDNA variability and geographic structure at the species level. Primers tRNA-Pro-L (5'-ACCATTGGCTCCCAAAGCTA-3') and H16498 (5'-CCTGAAGTAGGAACCAGATG-3') [29] were used for this amplification. PCR mix concentrations and cycling protocol were slightly different from COI with an MgCl2 concentration of 3 mM and an annealing temperature of 49°C.

Purification of PCR products in both cases was performed according to a standard ethanol precipitation protocol. Sequencing reactions were carried out using the BigDye Terminator version 3.1 Cycle Sequencing Kit (Applied Biosystems, Inc.). Products were sequenced in both directions on an ABI 3730 capillary sequencer following the manufacturer’s instructions. Individual sequences were edited with MEGA 6.06 [30], re-examined manually by visual inspection of raw Fluorigram data and then aligned with ClustalW (as implemented in MEGA 6.06).

### MtDNA sequence diversity and phylogeographic analyses

COI sequences from our samples were aligned to those from *Dentex* species and other closely related Sparidae species available in GenBank, using MEGA 6.06 [30]. All haplotypes from the final multiple alignment were used for the construction of a haplotype network with the median-joining network method [31] and default settings as implemented in the program Network 5.0.0.0 (Fluxus Technology Ltd., http://www.fluxus-engineering.com/). Networks are very helpful for the inferring of haplotype relationships and are used here in order to graphically represent species delimitation. A Maximum Likelihood phylogenetic tree was also constructed with the online software PHYML 3 [32] as implemented in the platform ATGC (http://www.atgc-montpellier.fr/). Model selection was made automatically by the program with the Smart Model Selection application [33], while branching support was surveyed with the aBayes method [34] and bootstrapping (10000 bootstraps). Basic diversity indices as the number of haplotypes (nh), nucleotide diversity (π), and haplotype (gene) diversity (h) for each sampled population were estimated using DnaSP 5 [35].

Using D-loop sequences, a hierarchical analysis of molecular variance (AMOVA) implemented in ARLEQUIN 3.5 was used to check for hypothesized patterns of spatial genetic structure assessing variance components between areas, between populations within areas and among individuals within populations [36]. Phylogeographic history of *D*. *dentex* was investigated using NETWORK 5.0.0.0 with default settings to reconstruct a median-joining network of haplotypes [31]. In order to explore demographic history, a mismatch distribution analysis in the whole data set of D-loop sequences (sum of sampled populations) was performed with ARLEQUIN 3.5. The simulated distribution of pairwise nucleotide differences (under the sudden expansion model and 10000 bootstrap replications) was compared with the observed distribution using the sum of square deviations (SSD) and the raggedness index (r) [36, 37].

### Microsatellite marker analysis

Microsatellite analyses were limited to the *D*. *dentex* samples alone, determined according to the COI marker. Eight microsatellite hypervariable markers established from other sparid species were applied to *D*. *dentex* (S1 Table data provides all technical information). For each pair of markers, one of the 5' ends of the two primers was end-labelled with a fluorescent dye, either 6-FAM, HEX or NED. Polymerase chain reactions (PCR) were performed using the Qiagen multiplex PCR kit (Qiagen, Courtaboeuf, France) in a final volume of 10 μL containing 3 μL of genomic DNA diluted at 10ng/μL, 5 μL of Qiagen PCR Master Mix, 1 μL of Qiagen Q-solution, and 1 μL of primers mix at 2 μM each (Eurofins MWG Operon, Ebersberg, Germany). Amplifications were carried out in a GeneAmp PCR System 2700 thermal cycler (Applied Biosystems) according to the supplier's instructions (Qiagen multiplex PCR kit): initial denaturation at 95°C for 15 min; followed by 35 cycles of denaturation at 94°C (30 s), annealing (56 and 58°C, as indicated in S1 Table, during 90 s) and extension (72°C, 60 s); with a final extension step at 60°C for 30 min. Amplified PCR fragments were then diluted and separated on an ABIPRISM 3130/xl/ sequencer (Applied Biosystems) with GeneScan 500 Rox dye size standards. Allele sizes were determined using the GeneMapper v4.1 software system (Applied Biosystems, Life Technologies). The genotype matrix was then constructed and used as a basis for all of the following statistical analyses.

A first selection of genotypes was processed; missing data were limited to 2 loci among 8 for each individual in the matrix to reduce statistical bias. The software GENETIX [38] was used, as for most of the following tests (diversity, F statistics, IBD, FCA).

Samples' polymorphism were estimated from the expected unbiased heterozygosity (Hnb) [39], observed heterozygosity (Ho) and average number of alleles per locus (A) parameters estimate the polymorphism of each sample.

The intra-population fixation index, Fis, was assessed with the Weir and Cockerham's f estimator [40], in order to check the Hardy-Weinberg equilibrium. Its significance was tested by comparing the natural Fis to 5,000 within the sample allele's permutated matrices. The Wright's index Fst, applied to nuclear genotypes, allowed for evaluating the differentiation between samples. Fst was calculated through the Weir and Cockerham's ϴ estimator; significance was tested for 5,000 permutations of genotypes between samples.

Isolation by distance (IBD) was tested by comparing a first matrix of genetic distances and a second matrix of geographical distances (obtained from Google Earth, tools path). Then the Mantel's test [41] was applied using the genetic distance based on Fst/(1-Fst) as recommended by Rousset [42]. The value of Z, the Mantel's coefficient, between the two matrices of distances was calculated with the true data, and then the significance of each test was assessed by comparing the true value with the series of pseudo-values produced by 5,000 permutations of the populations' order of one of the two matrices of distances.

Multivariate analyses display graphical representations of correlations between individuals depending on different variables. The factorial correspondence analysis, FCA, was based on an allelic disruptive matrix.

The existence and the number of sub-groups contained in the analyzed *D*. *dentex* sampling can be estimated by assignment method using the Bayesian clustering program STRUCTURE [43, 44, 45]. In our analysis, we selected the free clustering option with no prior population information and the admixture model. For the parameter settings, we set the number of MCMC repetitions at 200,000 after an initial burn-in of 100,000 repetitions. Values of K were set from 1 to 17 with 10 iterations for each value of K. Last, the ΔK Evanno's method [46] was used as an aid to define the most informative partition by calculating the variation of the rate of likelihood between K and K+1. These values correspond to local maximums of the curve ΔK function of K. The best K value is automatically calculated by the online program STRUCTURE HARVESTER [47].

## Results

### COI DNA barcoding

In total, 107 individuals from 19 locations (Table 1) were sequenced for the COI gene. Haplotype and nucleotide diversity values overall and for each sampling site are presented in Table 2. In addition, we included all the available COI sequences from *D*. *dentex*, as well as some sequences from other *Dentex* species (*D*. *gibbosus*, *D*. *maroccanus*, *D*. *macrophthalmus*, *D*. *angolensis*, *D*. *canariensis*) and other Sparidae species (*Pagrus caeruleostictus*, *Cheimerius nufar*, *Viridentex acromegalus*) already published in GenBank. A total of 66 sequences were downloaded from GenBank (S2 Table) and added to the final alignment/data set.

**Table 2:** Review of basic genetic diversity indices estimated for *Dentex dentex* populations in 16 of the 19 sampling localities and for both mitochondrial markers.

LOCALITY	COI 612bp				D-loop 444-455bp			
	N	nh	h	π	N	nh	h	π
North Aegean Sea (Greece)	5	2	0.4	0.00065	6	5	0.9	0.020089
Otranto (South Adriatic, Italy)	5	3	0.7	0.00131	5	4	0.9	0.021875
Lampedusa (Italy)	5	2	0.6	0.00078	5	5	1.0	0.017857
North Tunisia	2	2	1.0	0.00163	2	2	1.0	0.020089
South Tunisia	1	1	-	-	-	-	-	-
Sardinia (Italy)	6	4	0.8	0.00163	5	5	1.0	0.023214
Giraglia (Corsica, France)	5	1	0.0	-	5	5	1.0	0.022098
Bastia (Corsica, France)	6	3	0.6	0.00109	5	4	0.9	0.016295
St Florent (Corsica, France)	5	3	0.7	0.00196	3	3	1.0	0.039474
Galeria (Corsica, France)	4	3	0.8	0.00245	3	3	1.0	0.017857
Ajaccio (Corsica, France)	5	2	0.4	0.00131	5	5	1.0	0.028571
Bonifacio (Corsica, France)	5	2	0.4	0.00065	5	4	0.9	0.016923
***Corsica all sites***	***30***	***7***	***0*.*462***	***0*.*00371***	***26***	***22***	***0*.*987***	***0*.*022794***
St Raphael (France)	8	4	0.6	0.00123	6	6	1.0	0.022173
Baleares (Spain)	7	1	0.0	-	5	5	1.0	0.021875
Alicante (Spain)	5	3	0.7	0.00131	6	6	1.0	0.014435
Bay of Biscay (France)	8	2	0.3	0.00041	8	7	1.0	0.020727
**ALL**	**87**	**14**	**0.465**	**0.00097**	**79**	**57**	**0.985**	**0.021041**

N = number of individuals analyzed in each sampling locality; nh = number of haplotypes analyzed, h = haplotype diversity and π = nucleotide diversity.

**Fig 2:**
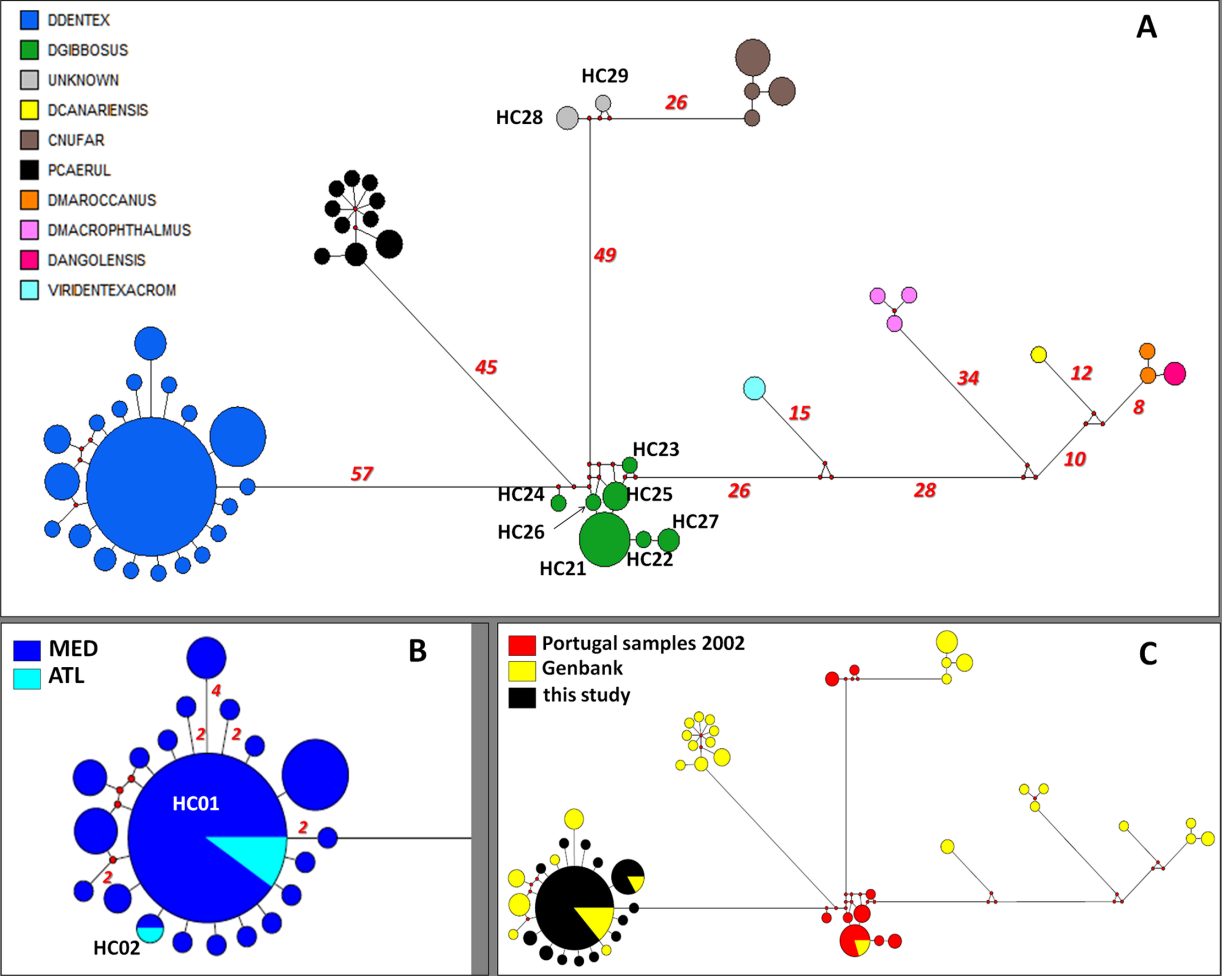
Median-joining network for the COI haplotypes identified in the common dentex DNA barcoding analysis (S2 Table). Each circle represents a haplotype and its size is proportional to haplotype frequency. Colors indicate different species in **A**, sampling regions in **B** for common dentex only, and source of all sequences used in **C**. Small red nodes represent possible median vectors while numbers indicate the number of nucleotide differences.

The construction of the COI median-joining network (Fig 2) was based on 51 different haplotypes (S2 and S4 Tables). All COI sequences of *D*. *dentex* published in GenBank and all the present study's sequences from 17 locations (Faro excluded) were clustered together in one major haplogroup in dark blue (Fig 2A). On the contrary, Bargelloni et al.'s [21] sampled sequences from Faro Portugal first attributed to *D*. *dentex* were significantly divergent and most of them (17 out of 20) constituted a distinct haplogroup (haplotypes HC21-HC27) in green. This Portuguese group, in which the published sequences for *D*. *gibbosus* are also included (HC21), probably corresponds to this last species. Haplotypes HC28 and HC29 were obtained from three samples of the same location [21] (Portugal) but seem to belong to a different haplogroup (in grey) which is significantly differentiated from *D*. *dentex* and *D*. *gibbosus* as well. Actually, these specific haplotypes (HC28, HC29) are closer to the *Chemerius nufar* haplogroup (in brown) than any other species.

**Fig 3:**
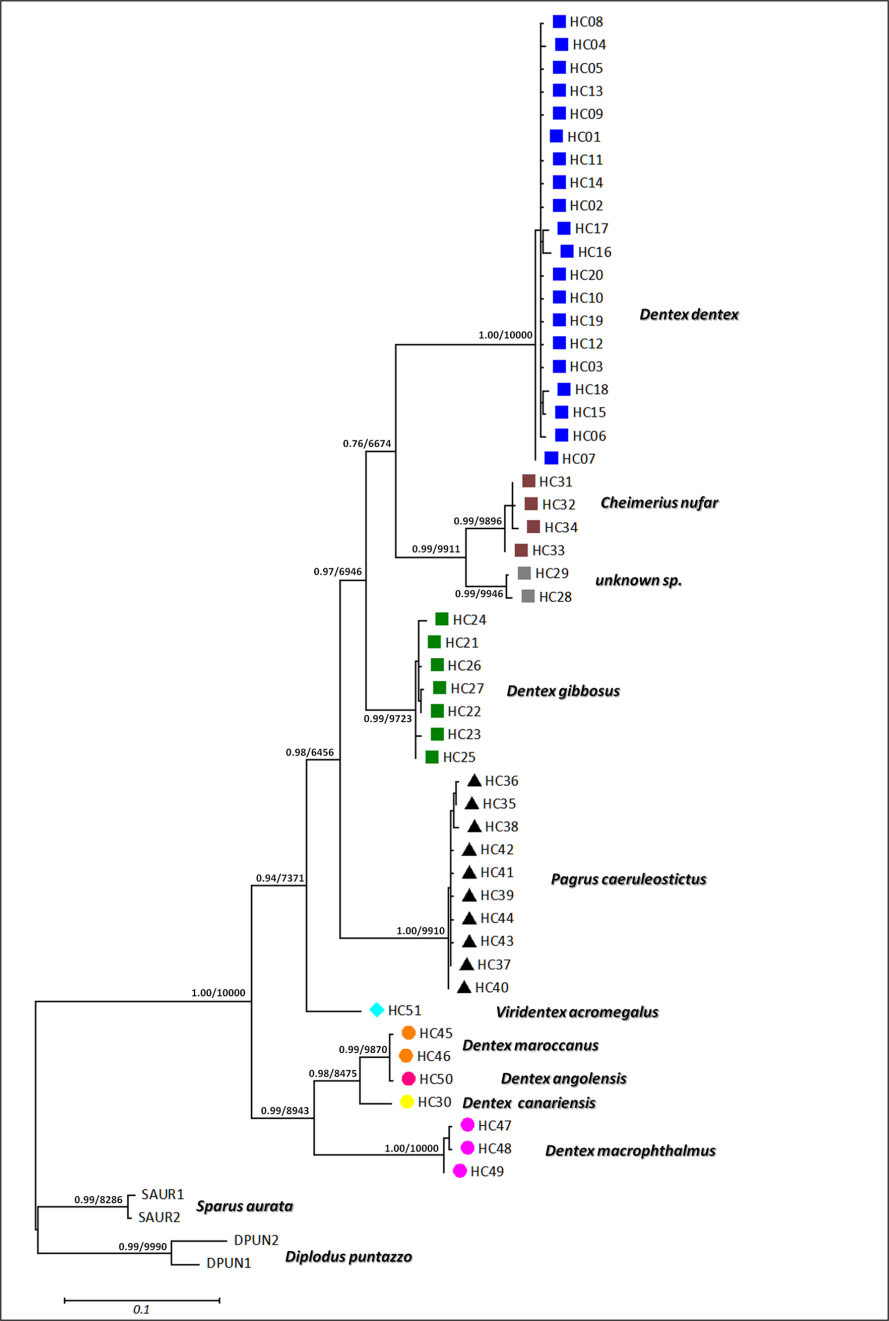
Maximum Likelihood phylogenetic tree constructed from COI haplotypes of *Dentex dentex* and 10 other species (S2 Table) with PHYML. Numbers on branches indicate aBayes / bootstrap support values (only values above 50% are shown).

The Maximum Likelihood phylogenetic analysis is based on the same marker, and the same samples gave similar results (Fig 3). For this tree construction, four extra sequences of *Sparus aurata* and *Diplodus puntazzo* were added as an outgroup (S2 Table). The best substitution model was selected by PHYML was HKY85 +G. Intraspecific genetic distances in *D*. *dentex* were low (less than 2%) and significantly lower than interspecific distances with all other putative species. DNA barcoding successfully discriminated between most of the putative species with the exception of *D*. *maroccanu*s and *D*. *angolensis* (Fig 3). Moreover this analysis shows that specimens captured in Faro, Portugal in 2002 belong to one or probably two different species (*D*. *gibbosus* and a "new" species with no COI sequence available in GenBank) and not to *D*. *dentex*.

The samples confirmed to belong to *D*. *dentex* by the COI marker include mainly Mediterranean specimens but also samples from the Atlantic Ocean, Bay of Biscay, and France collected in the present study (Fig 2B). Only these samples are involved in the following D-loop analysis.

### D-loop diversity in *D*. *dentex*

**Fig 4:**
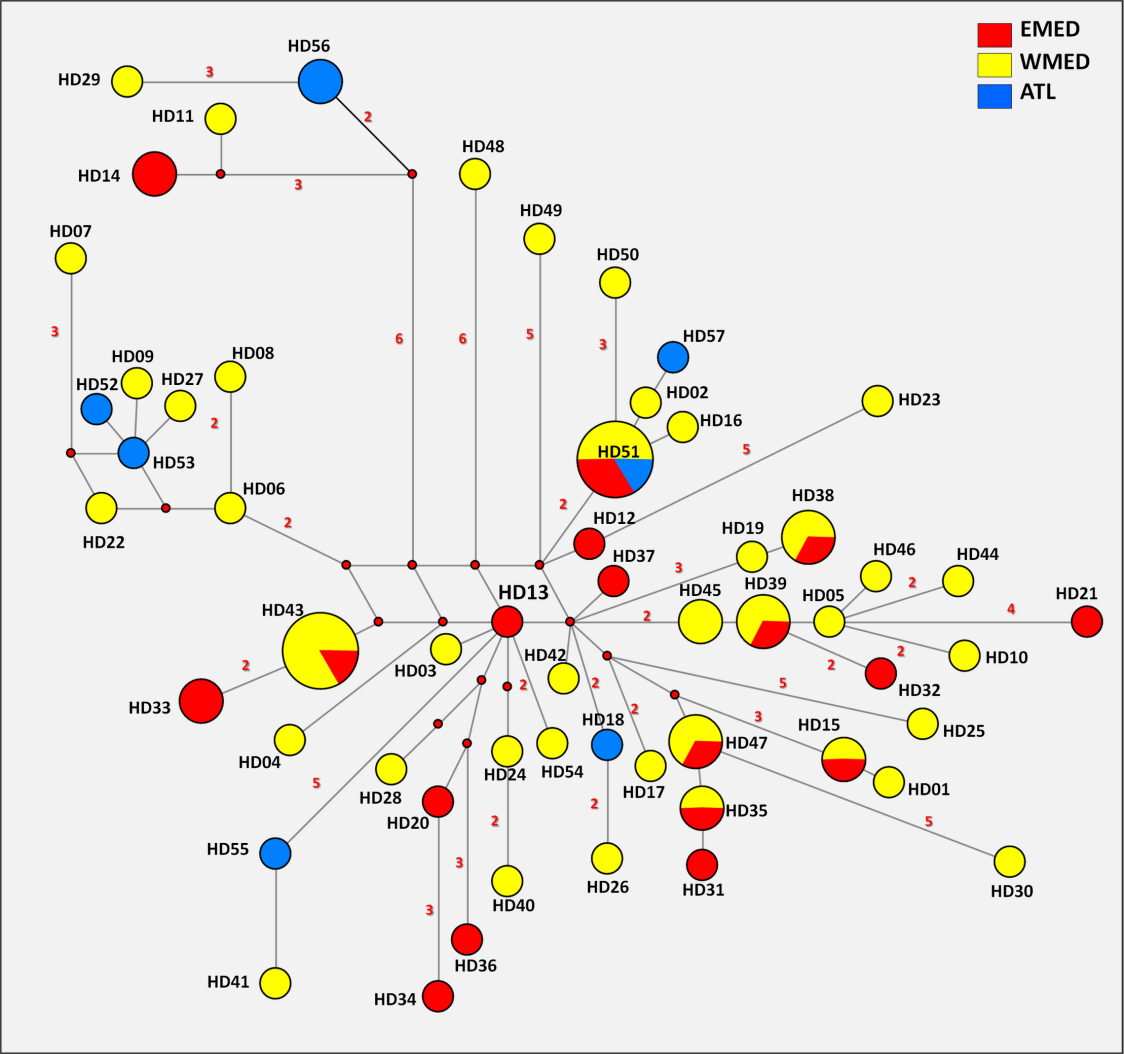
Median-joining network of 57 D-loop haplotypes of *Dentex dentex* (S3 Table). Each circle represents a haplotype and its size is proportional to haplotype frequency. Colors indicate different geographic origin (red: Eastern Mediterranean, yellow: Western Mediterranean, blue: Atlantic). Small red nodes represent possible median vectors while numbers indicate nucleotide differences greater than one between neighboring haplotypes.

Mitochondrial DNA polymorphism analysis was based on D-loop sequences of 79 common dentex individuals from 16 sampling locations (Table 1 and S4 Table), i.e. only those confirmed to belong to *D*. *dentex* by COI barcoding analysis. The sequence length ranged from 444 to 456 bp, a difference due to a duplication event. In total, 69 variable sites were found, with one 4-base and one 8-base duplication. Both global nucleotide diversity π and haplotype diversity h were high (π = 0.02104, h = 0.9854), indicating high levels of genetic variation in *D*. *dentex*. Estimations for each sampled population are summarized in Table 2. Among the 57 haplotypes identified, only few were distributed in more than one location (HD15, HD33, HD35, HD38, HD39, HD43, HD45, HD47, HD51), while most were sample-specific (see the median-joining D-loop network, Fig 4).

Nonetheless, differentiation between sampled populations was in most cases low and statistically not significant, as indicated by pairwise Fst estimations (S1 Fig). No significant spatial genetic structure was revealed by AMOVA analysis and testing of different groupings (Tamura & Nei distance matrix, 100,000 permutations). According to the AMOVA, the most probable scenario includes 3 areas/groups separated by the Gibraltar and the Siculo-Tunisian Straits: Eastern Mediterranean (Aegean, Crete, Adriatic, Lampedusa), Western Mediterranean (Tunisia, Sardinia, Corsica locations, France, Baleares, Spain) and Atlantic Ocean (Bay of Biscay). The largest component of variation (96.86%) was due to variation among individuals within populations (Fst = 0.031, p = 0.11), while variation among areas represents only 1.10% of total variation (FCT = 0.011, p = 0.24).

Phylogeographic analysis with median-joining networks also indicates a lack of spatial structure in *D*. *dentex*. In order to simplify the full median network initially produced which contained hypercubes (homoplasies), an analysis with MP option [48] was performed with NETWORK 5.0.0. for a final interpretation. The network (Fig 4) does not support the existence of any geographical structuring of haplotypes. Mismatch distribution showed a unimodal distribution of pairwise differences (S2 Fig) indicating that *D*. *dentex* has undergone periods of population growth (SSD = 0.00076, p = 0.67). A pattern of demographic expansion was also confirmed by the raggedness index, which failed to reject the null hypothesis of sudden population growth (r = 0.0044, p = 0.58). The existence of distinct clades with different demographic history would have resulted in multimodal distribution.

### Microsatellites diversity in *D*. *dentex*

Data consists of the genotyping of 312 *D*. *dentex* individuals for 8 microsatellite loci (described in S1 Table). General parameters of polymorphism and Fis parameter are given in Table 3. Significant intra-specific fixation indices are generally low but can be locally high (from 0.05526 to 0.30670).

**Table 3:** Estimated (He), unbiased (Hnb) and observed (Ho) heterozygosities and Fis values for the 17 samples of *Dentex dentex* genotyped with eight microsatellite loci.

	N	He	Hnb	Ho	A	F_IS_	F_IS_ signif.
Heraklion (North Crete, Greece)	24	0.723	0.739	0.740	10	-0.002	ns
North Aegean Sea (Greece)	13	0.690	0.721	0.523	6.6	0.284	***
Otranto (South Adriatic, Italy)	23	0.681	0.696	0.641	9.2	0.081	*
Sicily (Italy)	13	0.656	0.682	0.611	5.9	0.109	*
Lampedusa (Italy)	13	0.689	0.716	0.721	7.5	-0.007	ns
NorthTunisia	14	0.649	0.677	0.476	6.6	0.307	***
South Tunisia	14	0.677	0.705	0.558	7.4	0.216	***
Sardinia (Italy)	23	0.666	0.681	0.683	7.5	-0.002	ns
Giraglia (Corsica, France)	25	0.699	0.714	0.675	9.7	0.055	ns
St Florent (Corsica, France)	18	0.681	0.702	0.590	8.0	0.165	***
Galeria (Corsica, France)	31	0.690	0.703	0.631	10.2	0.104	**
Ajaccio (Corsica, France)	15	0.687	0.712	0.568	8.2	0.208	***
Bonifacio (Corsica, France)	24	0.673	0.689	0.618	8.9	0.105	**
St Raphael (France)	14	0.699	0.725	0.741	7.4	-0.023	ns
Baleares (Spain)	19	0.677	0.698	0.642	8.4	0.083	*
Alicante (Spain)	21	0.694	0.711	0.683	9.2	0.041	ns
Bay of Biscay (France)	8	0.658	0.703	0.734	5.9	-0.048	ns

Ns = not significant

* = p<0.05

** = p<0.01

*** = p<0.001.

Inter-sample differentiation (Fst) values are rarely significant (11 out of 128 tests before correction) and low (< 0.032) (Table 4). No geographic organization of the genetic diversity is observed since the seven inter-Mediterranean significant tests became non-significant after Bonferroni correction [49]. Moreover, organization among samples has also been investigated through multidimensional analyses, FCA (S3 Fig). This second analysis does not allow for the distinguishing of any structure among groups except for the additional fifteen Bargelloni et al. 2003 [21] samples confirming their misidentification.

**Table 4:** Calculation of inter-samples microsatellite differentiation for *Dentex dentex* (Weir and Cockerham 1984 estimator ɵ) and significance of each test.

	HE	NA	OT	SI	LA	NT	ST	SA	GI	SF	GA	AJ	BO	SR	BA	AL	BB
HE	0.000	0.000	0.007	0.025	-0.008	-0.013	0.011	-0.007	-0.004	0.009	0.008	-0.017	-0.005	0.003	0.001	0.002	0.015
NA		0.000	0.002	*0.016	0.005	-0.007	0.005	0.005	0.000	0.001	0.003	0.000	0.000	-0.007	-0.004	0.002	0.013
OT			0.000	0.009	-0.003	-0.001	0.001	*0.012	-0.001	-0.001	-0.002	0.001	0.004	-0.002	-0.006	0.001	0.012
SI				0.000	0.010	0.005	-0.007	*0.022	0.005	-0.001	0.005	0.012	0.004	0.001	0.001	0.011	0.025
LA					0.000	0.006	0.003	0.013	0.001	0.006	-0.002	-0.012	0.008	0.006	0.000	0.005	*0.020
NT						0.000	0.007	-0.004	-0.008	-0.003	-0.002	-0.013	-0.004	-0.004	-0.014	-0.007	0.011
ST							0.000	**0.026	0.003	-0.012	-0.001	0.008	0.005	-0.006	-0.003	0.004	0.004
SA								0.000	-0.004	*0.017	* 0.013	-0.003	0.008	*0.016	0.008	0.009	*0.032
GI									0.000	-0.004	0.000	-0.005	-0.002	0.005	-0.008	-0.005	0.013
SF										0.000	-0.005	0.004	-0.007	-0.000	-0.007	0.003	0.008
GA											0.000	-0.004	0.006	-0.001	-0.006	-0.003	*0.019
AJ												0.000	0.010	0.006	-0.003	-0.009	0.022
BO													0.000	0.000	-0.002	0.003	0.003
SR														0.000	-0.002	0.001	0.018
BA															0.000	-0.004	0.012
AL																0.000	*0.025
BB																	0.000

Significance:

* = p<0.05

** = p<0.01

*** = p<0.001.

HE = Heraklion (North Crete, Greece); NA = North Aegean Sea (Greece); OT = Otranto (South Adriatic, Italy); SI = Sicily (Italy); LA = Lampedusa (Italy); NT = NorthTunisia; ST = South Tunisia; SA = Sardinia (Italy); GI = Giraglia (Corsica, France); SF = St Florent (Corsica, France); GA = Galeria (Corsica, France); AJ = Ajaccio (Corsica, France); BO = Bonifacio (Corsica, France); SR = St Raphael (France); BA = Balearic Islands (Spain); AL = Alicante (Spain); BB = Bay of Biscay (France).

**Fig 5:**
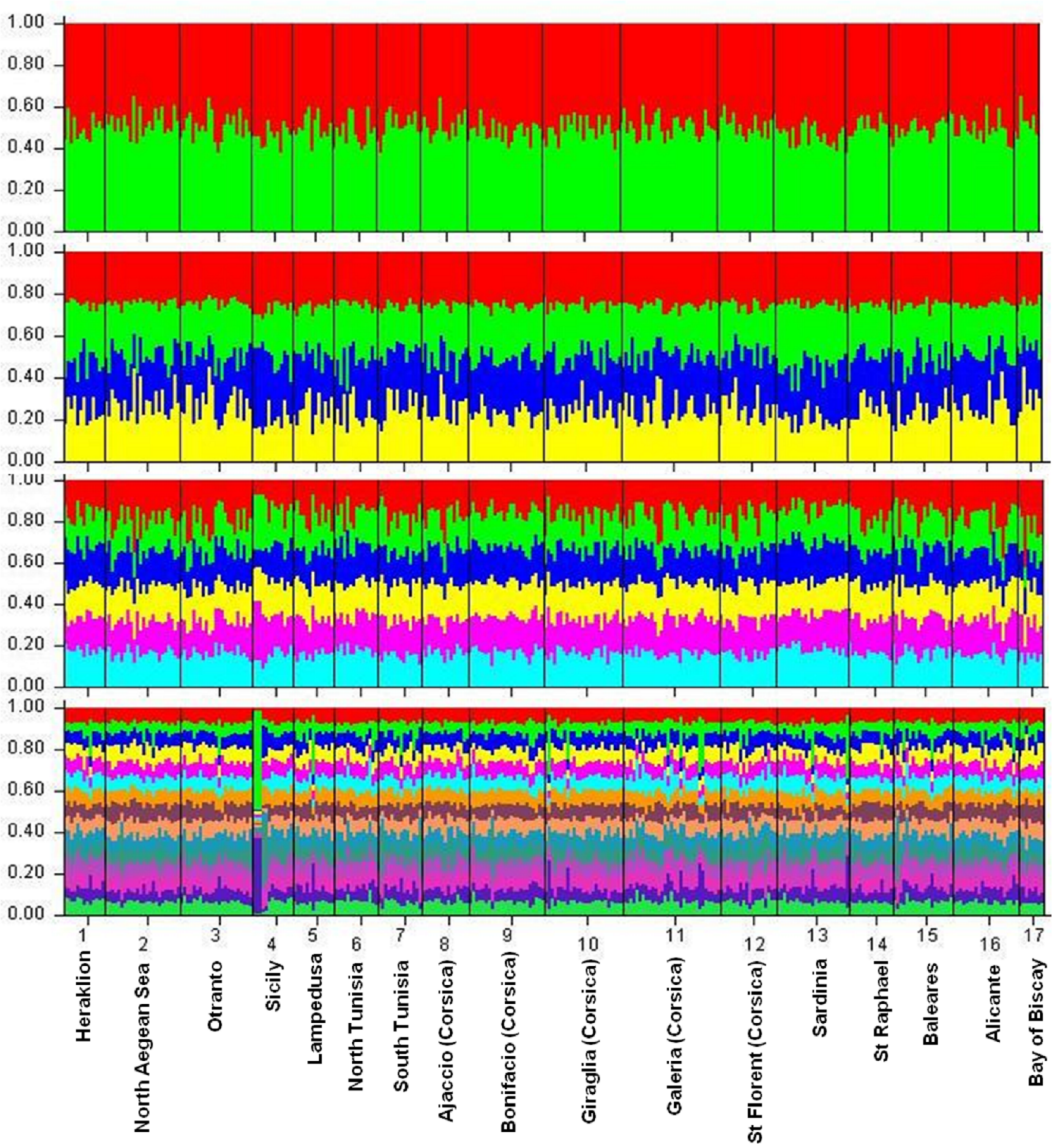
Assignment tests for 2, 4, 6 and 15 subdivisions for *Dentex dentex* population structure analysis.

Additionally, assignment Bayesian analysis led to the same result, i.e. that no structure is evidenced (Fig 5). The ΔK Evanno's method indicates a possible cluster number for K = 2 (Ln'(K) = -41.8). For this value of K, the plot from the STRUCTURE software does not allow determining a structure; each individual is assigned to both groups in equal proportion (± 50%). Thus all the individuals would form the same population. Likewise, for other bigger values of K (3 to 15), the same pattern is observed, cutting in rather than between individuals. Finally, the Mantel's test indicates a p = 0.05 probability of being right by rejecting the null hypothesis of independence of the two matrices. This limit of significance indicates a slight structure probably due to the geographical distance between the 17 stations.

## Discussion

### COI sequences: Species misidentification

The only genetic analysis available so far in *D*. *dentex* is that of Bargelloni et al. [21] using allozymes and mtDNA D-loop sequences. They also compared four other sparid species' genetic structures to that of *D*. *dentex*. This last species displayed the highest degree of genetic differentiation between Mediterranean and Atlantic populations. More specifically, in this study *D*. *dentex* presented a repeated motif in the 3' end of the sequenced region of all Mediterranean individuals that made the sequence alignment with other sequences difficult. This perturbing region was deleted and all further analyses were performed on a reduced data set of only 98 bp. This removal of unalignable regions could lead to a bias in the estimation of the divergence between the *D*. *dentex* haplotype groups [21]. In the same way, a morphological study using the same samples [22] have indicated a geographical gradient for the common dentex only, from Atlantic Ocean to the east of the Mediterranean.

In order to reach a rational phylogenetic picture, the COI sequences provided in the present analysis (107 sequences) were augmented by GenBank data (66 sequences). This was necessary for a comparison with other studies, but also to detect species misidentification considering COI sequences as a barcoding marker. Bargelloni et al. [21], using D-loop sequences from the same specimens from Faro, Portugal, have proposed the existence of two Mediterranean/Atlantic clades in *D*. *dentex*, but this finding is strongly questioned by our results. The median-joining network presented in Fig 2, based on COI sequences, clearly points out one major haplogroup in dark blue (Fig 2A) representing the species *Dentex dentex* according to numerous GenBank sequences, and a distinct haplogroup (haplotypes HC21-HC27) in green, in which most of Bargelloni's Portuguese group and the GenBank *D*. *gibbosus* sequences are included. The sequences used both in Bargelloni et al. [21] and Palma and Andrade [22] included a third group (in grey in Fig 2A) which is likely another unknown species. These results are further confirmed by nuclear (microsatellite) markers (S3 Fig). This type of misidentification is in fact quite common and has also occurred in other marine fishes [50, 51, 52]. As a consequence, the morphological gradient observed between Atlantic and Mediterranean seas by Palma and Andrade [22] is likely biased by the misidentification demonstrated here. The large unalignable region of D-loop reported by Bargelloni et al. [21] was likely a result of having multiple species in their data set.

In the present study, COI barcode analysis placed most Portuguese samples of Bargelloni et al. [21] within a distinct species that is probably *D*. *gibbosus*. Moreover, the Atlantic *D*. *dentex* sampled in the Bay of Biscay (close to Hendaye, France) for the present study are genetically undifferentiated from the Mediterranean populations. By its global coherence, the COI network seems very efficient in attributing the true species to each published and new sequences. Based on the current available sequences generated in the present study and from GenBank, a set of *D*. *dentex* samples were identified for further analyses. Support for monophyly of *D*. *dentex* cluster is high as shown Fig 3.

### MtDNA D-loop sequences

After attribution of the true species to each sequence, 57 *D*. *dentex* D-loop haplotypes were analyzed for a structure description. The D-loop median-joining network presented Fig 4 is a first method to find subgroups. However the three geographic categories (East-Mediterranean, West-Mediterranean and Atlantic populations) were randomly dispatched in the entire network evidencing no structure. Although most minority haplotypes are found in only one region, most frequent haplotypes were shared in two (HD38, HD43, HD47…) or three regions (HD51).

Such absence of structure in a very large range, from the Aegean Sea to French Atlantic regions, is quite surprising for a coastal species. However similar homogeneity has been observed in coastal-lagoonal fish species [53], jellyfish [54] and in crustacean species [55, 56], highlighting the probable importance of larval dispersal in a species range.

### Microsatellites loci

Microsatellites are generally highly variable, valuable nuclear markers. Their allele diversity generally guarantees a good description of the structure, even at a very local scale [57, 58]. Here, genotyping of 8 microsatellite loci on *D*. *dentex* populations from East-Mediterranean, West-Mediterranean on French and Spanish coast, Tyrrhenian islands and North African coasts, and finally in the Bay of Biscay on the Atlantic coast, i.e. about 4–5,000 km long, showed no indication of genetic structure at all. Inter samples Fst were not significant and assignment analysis was unable to distinguish subgroups, cutting inside individuals rather than between them, even for K = 2 (S4 and S5 Figs).

Mainly, nuclear markers did not allow for the detecting of any structure in Mediterranean and Atlantic populations of *D*. *dentex*, i.e. in the whole sampled distribution range, confirming the mtDNA results. This absence of structure has been observed in other species [59, 60, 61, 62]. These markers also highlighted the species misidentification involved in the Bargelloni et al. study [21]. However, the Mantel test is at the limit of significance. This suggests a slight east-west Mediterranean structure reminiscent of the observed Greece-to-Spain gradient thanks on morphometric measurements [22].

### High gene flow between Mediterranean common dentex populations and between the Mediterranean and Atlantic regions

In most genetic analyses of marine species, the use of variable markers allowed the description of subgroups in the Mediterranean and Atlantic range (e.g. *Platichthys flesus* [63]; *Scomber scombrus* [60]; *Lithognathus mormyrus* [64]; *Symphodus tinca* [65]). In fact, there are only few cases showing a total lack of genetic differentiation (e.g. *Sebastes mentella* [59]; *Scomber japonicus* [60]; *Epinephelus marginatus* [61]; *Conger conger* [62], and now *D*. *dentex*. According to Patarnello et al. [23], comparing the life history of several species of the same family with comparable biological traits can provide new understanding. Thus, the Sparidae family presents two patterns: *Lithognathus mormyrus*, *Spondyliosoma cantharus* [21] and *Diplodus puntazzo* [66] showing a clear Atlantic-Mediterranean differentiation, unlike *Pagellus bogaraveo*, *Pagrus pagrus* [21], *Diplodus sargus* [66] and *D*. *dentex* (the present study).

The different analyses performed based on microsatellites and mitochondrial DNA data all lead to the same observation: the absence of genetic structure in populations of common dentex. The inter-population fixation index and AMOVA's results are not significant. The FCA does not allow for the determination of groups among individuals, except the misidentified ones. The method of assignment does not give structured results. Only the IBD would reveal a slight structure separating the Mediterranean and Atlantic individuals.

It is remarkable that the sampling, all around Mediterranean, stretches along four years (2012–2014) for the new samples and along fifteen years if we consider the comparison with the Bargelloni et al. sampling [21]. This could be a bias if the Mediterranean populations were structured and dynamic with high level exchanges. However, the picture given by several markers is a very stable structure, without genetic contrast between regions. This diachronic sampling has so no effect on the general results.

The same pattern is observed around Corsica. Individuals all seem to belong to the same panmictic population. No Fst value is significant between the different stations and the other methods employed do not show any differentiation among populations. This absence of structure at this local Corsican scale has also been investigated with a multi-method approach [25] using a combination of markers that have different spatial and temporal scales of integration: microsatellite DNA markers, otolith shape analysis and parasites communities. However, although no genetic structure was found using microsatellites, a complex population structure is suggested in this study with the other marker distinguishing different “ecological population units” around Corsica. These markers, otoliths shape and parasitological tags, revealed an ecological timescale on finer temporal and spatial scales probably without genetic consequence. The holistic approach makes it possible to take into account phenotypic characteristics and thus to define more precisely some structures depending on local ecology and migrations [67].

The genetic homogeneity of many marine species is thought commonly to be due to two factors that minimize accumulation of genetic differences among populations: a large effective population size that limits genetic drift and life history characteristics that favor dispersal in continuous dynamic oceanic environments [68, 69]. This high connectivity between sampling sites may be due to the migration of adults, which are capable of large movements and/or larval dispersal. This larval dispersal may be, however, more limited than expected [70] by distance, for example. The IBD at the limit of significance and the holistic approach of Marengo et al. [25] would support this hypothesis. Among the three markers chosen for the holistic method, only the genetic nuclear markers did not detect any structure.

### Perspectives on the species distribution in the Atlantic Ocean

The established distribution range of *D*. *dentex* will probably change in the future for two reasons. First, in the Atlantic Ocean, the common dentex is considered present from the British Isles to Senegal [14]. But the misidentification described above could call into question this vast range. In the present survey, this question has not been fully developed (only one sample); more samples have to be done in the Atlantic Ocean, including the real distribution of the common dentex. The individuals observed at the extremities of the zone could belong to similar species. The species distribution in the Atlantic Ocean is scarce and more limited than previously thought. A detailed description of the sparids present along the European and African Atlantic coasts is necessary, sustained by DNA barcoding. Secondly, in a few years, global warming could again challenge the area of distribution. Indeed, the common dentex prefers a warm water environment [71]. Global warming is gradually impacting the temperature of Mediterranean waters. The seasonal stratification is still normal, but it is subject to oscillations. This phenomenon therefore has a positive impact on the common dentex, which can then extend its range to the north, along the Atlantic Ocean coasts.

## Supporting information

S1 Table Information on the eight microsatellite loci used in the present study and the PCR conditions. (CL168 and CL1014 [72]; Ds33 and Dxd16 [73]; SaGT41b [74]; SaI19 [75]; SauE82INRA and SauI41INRA [76]).
(DOCX)

S2 Table All information used for COI gene sequence analyses in Figs 2 and 3. np: not published.
(DOCX)

S3 Table D-loop haplotypes for each individual. (DOCX)

S4 Table COI and D-loop sequences included in mtDNA analyses. (DOCX)

S5 Table *Dentex dentex* genotypes at 8 microsatellites. (0 = no amplification). (DOCX)

S1 Fig Heat-map of D-loop pairwise Fst's between all pairs of localities. Asterisk indicates P≤ 0.05 in terms of statistical significance.
(TIFF)

S2 Fig Mismatch distribution for D-loop haplotypes of *Dentex dentex*. (TIFF)

S3 Fig Factorial Correspondences Analysis (FCA) of the whole *Dentex dentex* specimens. In the blue envelope are the very homogeneous samples of Mediterranean and Bay of Biscay origin. The red envelope gathers the Bargelloni et al. 2003 Portugal samples. Clearly, Bargelloni's Portuguese fish are heterogeneous (at least three clouds) and do not belong to *D*. *dentex* (except two individuals: see red arrows). Consequently, the Bay of Biscay samples are common dentex similar to those of Mediterranean populations.
(TIFF)

S4 Fig Diagram from Structure Harvester expressing Delta K according to K. (TIFF)

S5 Fig Diagram from Structure Harvester expressing likelihood according to K. Standard error bars are also indicated.
(TIFF)
