# Supplementary material for: Absence of spatial genetic structure in common dentex (Dentex dentex Linnaeus, 1758) in the Mediterranean Sea as evidenced by nuclear and mitochondrial molecular markers
Source: PLoS One. 2018 Sep 12;13(9):e0203866. doi: 10.1371/journal.pone.0203866 (PMC6135516; doi:10.1371/journal.pone.0203866)
Supplement: S1 Table — (CL168 and CL1014 [72]; Ds33 and Dxd16 [73]; SaGT41b [74]; SaI19 [75]; SauE82INRA and SauI41INRA [76]). (DOCX) [file pone.0203866.s001.docx]

**Supporting information**

**S1 Table Information on the eight microsatellite loci used in the present study and the PCR conditions** (*CL168* and *CL1014* [Vogiatzi et al. 2011]; *Ds33* and *Dxd16* [De la Herran et al. 2005]; *SaGT41b* [Batargias et al 1999]; *SaI19* [Brown et al. 2005]; *SauE82INRA* and *SauI41INRA* [Launey et al 2003])

| **Locus** | **Structure** | **Ann. t°** | **MgCl2** | **O*riginal species*** |
| --- | --- | --- | --- | --- |
| ***CL168*** | (CTA)9 | 58 | 1.5 | *Sparus aurata* |
| ***CL1014*** | (CA)16 | 58 | 1.5 | *Sparus aurata* |
| ***Ds33*** | (GT)17C(GT)2 | 56 | 1.5 | *Diplodus sargus* |
| ***Dxd16*** | (GT)15(GA)18 | 56 | 2 | *Dentex dentex* |
| ***SaGT41b*** | (AC)13 | 56 | 1.5 | *Sparus aurata* |
| ***SaI19*** | (GT)25 | 56 | 1.5 | *Sparus aurata* |
| ***SauE82INRA*** | (CA)12AA(CA)7 | 56 | 1.5 | *Sparus aurata* |
| ***SauI41INRA*** | (CA)9CG(CA)4CG(CA)13 | 56 | 1.5 | *Sparus aurata* |

Vogiatzi E, Lagnel J, Pakaki V, Louro B, Canario AVM, Reinhardt R, et al. In silico mining and characterization of simple sequence repeats from gilthead sea bream (*Sparus aurata*) expressed sequence tags (EST-SSRs); PCR amplification, polymorphism evaluation and multiplexing and cross-species assays. Marine Genomics 2011; 4(2): 83-91.

De la Herrán R, Magoulas A, Garrido-Ramos MA, Ruiz-Rejón C, Ruiz-Rejón M, Zouros E. Desarrollo de microsatélites en tres especies de espáridos con interés en acuicultura. In: La acuicultura como actividad económica de las zonas costeras. IX Congreso Nacional de Acuicultura. Ed. Consejería de Agricultura y Pesca. Junta de Andalucia. Sevilla. Spain, 2005. pp133-136.

Batargias C, Dermitzakis E, Magoulas A, Zouros E. Characterization of six polymorphic microsatellite markers in gilthead seabream, *Sparus aurata* (Linnaeus 1758). Molecular Ecology 1999; 8: 897-8.

Brown RC, Tsalavouta M, Terzoglou V, Magoulas A, Mcandrew BJ. Additional microsatellites for *Sparus aurata* and cross-species amplification within the Sparidae family. Molecular Ecology Notes 2005; 5(3): 605-7.

Launey S, Krieg F, Haffray P, Bruant JS, Vannier A, Guyomard R. Twelve new microsatellite markers for gilted seabream (*Sparus aurata* L.): characterization, polymorphism and linkage. Molecular Ecology Notes 2003; 3(3): 457-9.
