# Supplementary material for: Absence of spatial genetic structure in common dentex (Dentex dentex Linnaeus, 1758) in the Mediterranean Sea as evidenced by nuclear and mitochondrial molecular markers
Source: PLoS One. 2018 Sep 12;13(9):e0203866. doi: 10.1371/journal.pone.0203866 (PMC6135516; doi:10.1371/journal.pone.0203866)
Supplement: S2 Table — np: not published. (DOCX) [file pone.0203866.s002.docx]

**Supporting information**

**S2 Table. All information used for COI gene sequence analyses in Fig 2 and 3. np: not published**

|  |  |  | **GenBank** |  |  |  |
| --- | --- | --- | --- | --- | --- | --- |
| **Specimen** | | **Sampling** | **accession** |  |  |  |
| **N°** | **ID** | **location** | **number** | **Haplotype** | **Reference** | ***Species*** |
| 1 | ATL01 | Bay of Biscay (France) | MG981563 | HC01 | this study | *D. dentex* |
| 2 | ATL02 | Bay of Biscay (France) | MG981564 | HC01 | this study | *D. dentex* |
| 3 | ATL03 | Bay of Biscay (France) | MG981565 | HC01 | this study | *D. dentex* |
| 4 | ATL04 | Bay of Biscay (France) | MG981566 | HC01 | this study | *D. dentex* |
| 5 | ATL06 | Bay of Biscay (France) | MG981568 | HC01 | this study | *D. dentex* |
| 6 | ATL07 | Bay of Biscay (France) | MG981569 | HC01 | this study | *D. dentex* |
| 7 | ATL08 | Bay of Biscay (France) | MG981570 | HC01 | this study | *D. dentex* |
| 8 | DT001 | Ajaccio (Corsica, France) | MG981571 | HC01 | this study | *D. dentex* |
| 9 | DT004 | Bonifacio (Corsica, France) | MG981572 | HC01 | this study | *D. dentex* |
| 10 | DT007 | Bonifacio (Corsica, France) | MG981574 | HC01 | this study | *D. dentex* |
| 11 | DT011 | Bonifacio (Corsica, France) | MG981575 | HC01 | this study | *D. dentex* |
| 12 | DT013 | Bonifacio (Corsica, France) | MG981576 | HC01 | this study | *D. dentex* |
| 13 | DT031 | Giraglia (Corsica, France) | MG981577 | HC01 | this study | *D. dentex* |
| 14 | DT032 | Giraglia (Corsica, France) | MG981578 | HC01 | this study | *D. dentex* |
| 15 | DT033 | Ajaccio (Corsica, France) | MG981579 | HC01 | this study | *D. dentex* |
| 16 | DT036 | Ajaccio (Corsica, France) | MG981581 | HC01 | this study | *D. dentex* |
| 17 | DT038 | Ajaccio (Corsica, France) | MG981582 | HC01 | this study | *D. dentex* |
| 18 | DT054 | St Florent (Corsica, France) | MG981584 | HC01 | this study | *D. dentex* |
| 19 | DT055 | St Florent (Corsica, France) | MG981585 | HC01 | this study | *D. dentex* |
| 20 | DT059 | St Florent (Corsica, France) | MG981587 | HC01 | this study | *D. dentex* |
| 21 | DT069 | Galeria (Corsica, France) | MG981588 | HC01 | this study | *D. dentex* |
| 22 | DT072 | Galeria (Corsica, France) | MG981590 | HC01 | this study | *D. dentex* |
| 23 | DT120 | Alicante (Spain) | MG981613 | HC01 | this study | *D. dentex* |
| 24 | DT122 | Alicante (Spain) | MG981614 | HC01 | this study | *D. dentex* |
| 25 | DT125 | Alicante (Spain) | MG981615 | HC01 | this study | *D. dentex* |
| 26 | DT145 | Otranto (South Adriatic, Italy) | MG981617 | HC01 | this study | *D. dentex* |
| 27 | DT150 | Otranto (South Adriatic, Italy) | MG981620 | HC01 | this study | *D. dentex* |
| 28 | DT153 | Otranto (South Adriatic, Italy) | MG981621 | HC01 | this study | *D. dentex* |
| 29 | DT168 | Heraklion (North Crete, Greece) | MG981622 | HC01 | this study | *D. dentex* |
| 30 | DT169 | Heraklion (North Crete, Greece) | MG981623 | HC01 | this study | *D. dentex* |
| 31 | DT172 | Heraklion (North Crete, Greece) | MG981625 | HC01 | this study | *D. dentex* |
| 32 | DT180 | Heraklion (North Crete, Greece) | MG981626 | HC01 | this study | *D. dentex* |
| 33 | DT194 | North Aegean (Greece) | MG981628 | HC01 | this study | *D. dentex* |
| 34 | DT196 | North Aegean (Greece) | MG981629 | HC01 | this study | *D. dentex* |
| 35 | DT197 | North Aegean (Greece) | MG981630 | HC01 | this study | *D. dentex* |
| 36 | DT199 | North Aegean (Greece) | MG981631 | HC01 | this study | *D. dentex* |
| 37 | DT217 | North Tunisia | MG981632 | HC01 | this study | *D. dentex* |
| 38 | DT246 | South Tunisia | MG981634 | HC01 | this study | *D. dentex* |
| 39 | DT277 | St Raphael (France) | MG981635 | HC01 | this study | *D. dentex* |
| 40 | DT280 | St Raphael (France) | MG981638 | HC01 | this study | *D. dentex* |
| 41 | DT283 | St Raphael (France) | MG981640 | HC01 | this study | *D. dentex* |
| 42 | DT284 | St Raphael (France) | MG981641 | HC01 | this study | *D. dentex* |
| 43 | DT285 | St Raphael (France) | MG981642 | HC01 | this study | *D. dentex* |
| 44 | DT288 | Giraglia (Corsica, France) | MG981643 | HC01 | this study | *D. dentex* |
| 45 | DT294 | Giraglia (Corsica, France) | MG981644 | HC01 | this study | *D. dentex* |
| 46 | DT346 | Giraglia (Corsica, France) | MG981645 | HC01 | this study | *D. dentex* |
| 47 | DT391 | Bastia (Corsica, France) | MG981646 | HC01 | this study | *D. dentex* |
| 48 | DT395 | Bastia (Corsica, France) | MG981648 | HC01 | this study | *D. dentex* |
| 49 | DT396 | Bastia (Corsica, France) | MG981649 | HC01 | this study | *D. dentex* |
| 50 | DT398 | Bastia (Corsica, France) | MG981651 | HC01 | this study | *D. dentex* |
| 51 | DT431 | Baleares (Spain) | MG981652 | HC01 | this study | *D. dentex* |
| 52 | DT432 | Baleares (Spain) | MG981653 | HC01 | this study | *D. dentex* |
| 53 | DT433 | Baleares (Spain) | MG981654 | HC01 | this study | *D. dentex* |
| 54 | DT434 | Baleares (Spain) | MG981655 | HC01 | this study | *D. dentex* |
| 55 | DT437 | Baleares (Spain) | MG981656 | HC01 | this study | *D. dentex* |
| 56 | DT439 | Baleares (Spain) | MG981657 | HC01 | this study | *D. dentex* |
| 58 | DT456 | Lampedusa (Italy) | MG981659 | HC01 | this study | *D. dentex* |
| 59 | DT457 | Lampedusa (Italy) | MG981660 | HC01 | this study | *D. dentex* |
| 60 | DT459 | Lampedusa (Italy) | MG981662 | HC01 | this study | *D. dentex* |
| 61 | DT476 | Sardinia (Italy) | MG981664 | HC01 | this study | *D. dentex* |
| 62 | DT480 | Sardinia (Italy) | MG981666 | HC01 | this study | *D. dentex* |
| 63 | DT490 | Sardinia (Italy) | MG981667 | HC01 | this study | *D. dentex* |
| 64 | DD01 | np | JQ623927 | HC01 | np | *D. dentex* |
| 65 | DD02 | Turkey | KC500461 | HC01 | Keskin & Atar 2013 | *D. dentex* |
| 66 | DD03 | Turkey | KC500459 | HC01 | Keskin & Atar 2013 | *D. dentex* |
| 67 | DD04 | Turkey | KC500458 | HC01 | Keskin & Atar 2013 | *D. dentex* |
| 68 | DD05 | Turkey | KC500457 | HC01 | Keskin & Atar 2013 | *D. dentex* |
| 69 | DD06 | Turkey | KC500456 | HC01 | Keskin & Atar 2013 | *D. dentex* |
| 70 | DD07 | Turkey | KC500455 | HC01 | Keskin & Atar 2013 | *D. dentex* |
| 71 | DD08 | Turkey | KC500463 | HC01 | Keskin & Atar 2013 | *D. dentex* |
| 72 | DD23 | np | KT883591 | HC01 | Sanciangco *et al.* 2016 | *D. dentex* |
| 73 | DD27 | Mediterranean | KJ012327 | HC01 | Armani *et al.* 2015 | *D. dentex* |
| 74 | ATL05 | Bay of Biscay (France) | MG981567 | HC02 | this study | *D. dentex* |
| 75 | DT397 | Bastia (Corsica, France) | MG981650 | HC02 | this study | *D. dentex* |
| 76 | DT006 | Bonifacio (Corsica, France) | MG981573 | HC03 | this study | *D. dentex* |
| 77 | DT070 | Galeria (Corsica, France) | MG981589 | HC03 | this study | *D. dentex* |
| 78 | DT117 | Alicante (Spain) | MG981612 | HC03 | this study | *D. dentex* |
| 79 | DT149 | Otranto (South Adriatic, Italy) | MG981619 | HC03 | this study | *D. dentex* |
| 80 | DT171 | Heraklion (North Crete, Greece) | MG981624 | HC03 | this study | *D. dentex* |
| 81 | DT193 | North Aegean (Greece) | MG981627 | HC03 | this study | *D. dentex* |
| 82 | DT225 | North Tunisia | MG981633 | HC03 | this study | *D. dentex* |
| 83 | DT392 | Bastia (Corsica, France) | MG981647 | HC03 | this study | *D. dentex* |
| 84 | DT458 | Lampedusa (Italy) | MG981661 | HC03 | this study | *D. dentex* |
| 85 | DT460 | Lampedusa (Italy) | MG981663 | HC03 | this study | *D. dentex* |
| 86 | DD22 | Egypt | KP308272 | HC03 | np | *D. dentex* |
| 87 | DD24 | Western Mediterranean | KJ012326 | HC03 | Armani *et al.* 2015 | *D. dentex* |
| 88 | DT035 | Ajaccio (Corsica, France) | MG981580 | HC04 | this study | *D. dentex* |
| 89 | DT053 | St Florent (Corsica, France) | MG981583 | HC05 | this study | *D. dentex* |
| 90 | DT477 | Sardinia (Italy) | MG981665 | HC05 | this study | *D. dentex* |
| 91 | DT058 | St Florent (Corsica, France) | MG981586 | HC06 | this study | *D. dentex* |
| 92 | DT073 | Galeria (Corsica, France) | MG981591 | HC07 | this study | *D. dentex* |
| 93 | DT129 | Alicante (Spain) | MG981616 | HC08 | this study | *D. dentex* |
| 94 | DT147 | Otranto (South Adriatic, Italy) | MG981618 | HC09 | this study | *D. dentex* |
| 95 | DT278 | St Raphael (France) | MG981636 | HC10 | this study | *D. dentex* |
| 96 | DT279 | St Raphael (France) | MG981637 | HC11 | this study | *D. dentex* |
| 97 | DT281 | St Raphael (France) | MG981639 | HC12 | this study | *D. dentex* |
| 98 | DT491 | Sardinia (Italy) | MG981668 | HC13 | this study | *D. dentex* |
| 99 | DT492 | Sardinia (Italy) | MG981669 | HC14 | this study | *D. dentex* |
| 100 | DD09 | Turkey | KC500462 | HC15 | Keskin & Atar 2013 | *D. dentex* |
| 101 | DD10 | Turkey | KC500460 | HC15 | Keskin & Atar 2013 | *D. dentex* |
| 102 | DD11 | Turkey | KC500464 | HC15 | Keskin & Atar 2013 | *D. dentex* |
| 103 | DD12 | Turkey | KC500465 | HC15 | Keskin & Atar 2013 | *D. dentex* |
| 104 | DD13 | Turkey | KC500466 | HC15 | Keskin & Atar 2013 | *D. dentex* |
| 105 | DD14 | Turkey | KC500467 | HC16 | Keskin & Atar 2013 | *D. dentex* |
| 106 | DD15 | Turkey | KC500468 | HC16 | Keskin & Atar 2013 | *D. dentex* |
| 107 | DD16 | Turkey | KC500469 | HC16 | Keskin & Atar 2013 | *D. dentex* |
| 108 | DD17 | Turkey | KC500470 | HC16 | Keskin & Atar 2013 | *D. dentex* |
| 109 | DD18 | Turkey | KC500471 | HC17 | Keskin & Atar 2013 | *D. dentex* |
| 110 | DD19 | Turkey | KC500472 | HC17 | Keskin & Atar 2013 | *D. dentex* |
| 111 | DD20 | Turkey | KC500453 | HC17 | Keskin & Atar 2013 | *D. dentex* |
| 112 | DD21 | Turkey | KC500454 | HC18 | Keskin & Atar 2013 | *D. dentex* |
| 113 | DD25 | Western Mediterranean | KJ012328 | HC19 | Armani *et al.* 2015 | *D. dentex* |
| 115 | DGIB01 | Eastern Central Atlantic Ocean | KJ012330 | HC21 | Armani *et al.* 2015 | *D. gibbosus* |
| 116 | DGIB02 | Egypt | LC152206 | HC21 | np | *D. gibbosus* |
| 117 | DT086 | Faro (Bargelloni samples) | MG981592 | HC21 | this study | *D. gibbosus* |
| 118 | DT099 | Faro (Bargelloni samples) | MG981603 | HC21 | this study | *D. gibbosus* |
| 119 | DT100 | Faro (Bargelloni samples) | MG981604 | HC21 | this study | *D. gibbosus* |
| 120 | DT102 | Faro (Bargelloni samples) | MG981606 | HC21 | this study | *D. gibbosus* |
| 121 | DT103 | Faro (Bargelloni samples) | MG981607 | HC21 | this study | *D. gibbosus* |
| 122 | DT104 | Faro (Bargelloni samples) | MG981608 | HC21 | this study | *D. gibbosus* |
| 123 | DT105 | Faro (Bargelloni samples) | MG981609 | HC21 | this study | *D. gibbosus* |
| 124 | DT106 | Faro (Bargelloni samples) | MG981610 | HC21 | this study | *D. gibbosus* |
| 125 | DT087 | Faro (Bargelloni samples) | MG981593 | HC22 | this study | *D. gibbosus* |
| 126 | DT092 | Faro (Bargelloni samples) | MG981598 | HC23 | this study | *D. gibbosus* |
| 127 | DT094 | Faro (Bargelloni samples) | MG981599 | HC24 | this study | *D. gibbosus* |
| 128 | DT091 | Faro (Bargelloni samples) | MG981597 | HC25 | this study | *D. gibbosus* |
| 129 | DT095 | Faro (Bargelloni samples) | MG981600 | HC25 | this study | *D. gibbosus* |
| 130 | DT096 | Faro (Bargelloni samples) | MG981601 | HC25 | this study | *D. gibbosus* |
| 131 | DT097 | Faro (Bargelloni samples) | MG981602 | HC26 | this study | *D. gibbosus* |
| 132 | DT101 | Faro (Bargelloni samples) | MG981605 | HC27 | this study | *D. gibbosus* |
| 133 | DT107 | Faro (Bargelloni samples) | MG981611 | HC27 | this study | *D. gibbosus* |
| 134 | DT088 | Faro (Bargelloni samples) | MG981594 | HC28 | this study | ? |
| 135 | DT089 | Faro (Bargelloni samples) | MG981595 | HC28 | this study | ? |
| 136 | DT090 | Faro (Bargelloni samples) | MG981596 | HC29 | this study | ? |
| 137 | DCAN01 | Eastern Central Atlantic Ocean | KJ012325 | HC30 | Armani *et al.* 2015 | *D. canariensis* |
| 138 | CNUF01 | South Africa | JF493134 | HC31 | np | *Cheimerius nufar* |
| 139 | CNUF03 | South Africa | HQ611102 | HC32 | Cawthorn *et al.* 2011 | *Cheimerius nufar* |
| 140 | CNUF04 | South Africa | HQ611103 | HC32 | Cawthorn *et al.* 2011 | *Cheimerius nufar* |
| 141 | CNUF05 | South Africa | HQ611104 | HC32 | Cawthorn *et al.* 2011 | *Cheimerius nufar* |
| 142 | CNUF06 | Western Indian Ocean | KJ012317 | HC32 | Armani *et al.* 2015 | *Cheimerius nufar* |
| 143 | CNUF07 | Western Indian Ocean | KJ012319 | HC32 | Armani *et al.* 2015 | *Cheimerius nufar* |
| 144 | CNUF09 | Western Indian Ocean | KJ012318 | HC33 | Armani *et al.* 2015 | *Cheimerius nufar* |
| 145 | CNUF02 | South Africa | HQ611105 | HC34 | Cawthorn *et al.* 2011 | *Cheimerius nufar* |
| 146 | CNUF08 | Western Indian Ocean | KJ012316 | HC34 | Armani *et al.* 2015 | *Cheimerius nufar* |
| 147 | CNUF10 | np | KT883592 | HC34 | Sanciangco *et al.* 2016 | *Cheimerius nufar* |
| 148 | PCAE01 | np | JN868714 | HC35 | np | *Pagrus caeruleostictus* |
| 149 | PCAE10 | Western Mediterranean | KJ012409 | HC35 | Armani *et al.* 2015 | *Pagrus caeruleostictus* |
| 150 | PCAE02 | South China Sea | KF857267 | HC36 | np | *Pagrus caeruleostictus* |
| 151 | PCAE03 | South China Sea | KF857268 | HC37 | np | *Pagrus caeruleostictus* |
| 152 | PCAE04 | Israel | KM538479 | HC38 | np | *Pagrus caeruleostictus* |
| 153 | PCAE05 | Israel | KM538481 | HC38 | np | *Pagrus caeruleostictus* |
| 154 | PCAE09 | Israel | KM538480 | HC38 | np | *Pagrus caeruleostictus* |
| 155 | PCAE06 | Israel | KM538478 | HC39 | np | *Pagrus caeruleostictus* |
| 156 | PCAE07 | Israel | KM538477 | HC40 | np | *Pagrus caeruleostictus* |
| 157 | PCAE08 | Israel | KM538476 | HC41 | np | *Pagrus caeruleostictus* |
| 158 | PCAE11 | Eastern Mediterranean | KJ012407 | HC42 | Armani *et al.* 2015 | *Pagrus caeruleostictus* |
| 159 | PCAE12 | Eastern Central Atlantic Ocean | KJ012411 | HC43 | Armani *et al.* 2015 | *Pagrus caeruleostictus* |
| 160 | PCAE13 | Eastern Central Atlantic Ocean | KJ012408 | HC44 | Armani *et al.* 2015 | *Pagrus caeruleostictus* |
| 161 | DMAR01 | Eastern Mediterranean Sea | KJ012335 | HC45 | Armani *et al.* 2015 | *D. maroccanus* |
| 162 | DMAR02 | Eastern Mediterranean Sea | KJ012334 | HC46 | Armani *et al.* 2015 | *D. maroccanus* |
| 163 | DMAC01 | Eastern Mediterranean Sea | KJ012331 | HC47 | Armani *et al.* 2015 | *D. macrophthalmus* |
| 164 | DMAC02 | Eastern Mediterranean Sea | KJ012333 | HC48 | Armani *et al.* 2015 | *D. macrophthalmus* |
| 165 | DMAC03 | Eastern Mediterranean Sea | KJ012332 | HC49 | Armani *et al.* 2015 | *D. macrophthalmus* |
| 166 | DANG01 | Eastern Central Atlantic Ocean | KJ012323 | HC50 | Armani *et al.* 2015 | *D. angolensis* |
| 167 | DANG02 | Eastern Central Atlantic Ocean | KJ012321 | HC50 | Armani *et al.* 2015 | *D. angolensis* |
| 168 | VACR01 | Eastern Central Atlantic Ocean | KJ012449 | HC51 | Armani *et al.* 2015 | *Viridentexacromegalus* |
| 169 | VACR02 | np | KT883595 | HC51 | Sanciangco *et al.* 2016 | *Viridentexacromegalus* |
| 170 | SAUR1 | Western Mediterranean | KJ012432 | SAUR1 | Armani *et al.* 2015 | *Sparus aurata* |
| 171 | TR1587EK | Turkey | KC501566 | SAUR2 | Keskin & Alar 2013 | *Sparus aurata* |
| 172 | DPUN1 | Western Central Atlantic | KJ012347 | DPUN1 | Armani *et al.* 2015 | *Diplodus puntazzo* |
| 173 | TR598EK | Turkey | KC500572 | DPUN2 | Keskin & Alar 2013 | *Diplodus puntazzo* |
