# Supplementary material for: Absence of spatial genetic structure in common dentex (Dentex dentex Linnaeus, 1758) in the Mediterranean Sea as evidenced by nuclear and mitochondrial molecular markers
Source: PLoS One. 2018 Sep 12;13(9):e0203866. doi: 10.1371/journal.pone.0203866 (PMC6135516; doi:10.1371/journal.pone.0203866)
Supplement: S3 Table — (DOCX) [file pone.0203866.s003.docx]

**Supportive information**

**S3 Table. D-loop haplotypes for each individual.**

**Sample ID Sampling location GenBank Accession D-loop 444-455bp Haplotypes**

| 1 | DT006 | Bonifacio (Corsica, France) | MG981670 | HD01 |
| --- | --- | --- | --- | --- |
| 2 | DT011 | Bonifacio (Corsica, France) | MG981671 | HD02 |
| 3 | DT013 | Bonifacio (Corsica, France) | MG981672 | HD03 |
| 4 | DT031 | Giraglia (Corsica, France) | MG981673 | HD04 |
| 5 | DT032 | Giraglia (Corsica, France) | MG981674 | HD05 |
| 6 | DT288 | Giraglia (Corsica, France) | MG981675 | HD06 |
| 7 | DT294 | Giraglia (Corsica, France) | MG981676 | HD07 |
| 8 | DT346 | Giraglia (Corsica, France) | MG981677 | HD08 |
| 9 | DT033 | Ajaccio (Corsica, France) | MG981678 | HD09 |
| 10 | DT035 | Ajaccio (Corsica, France) | MG981679 | HD10 |
| 11 | DT038 | Ajaccio (Corsica, France) | MG981680 | HD11 |
| 12 | DT145 | Otranto (South Adriatic, Italy) | MG981681 | HD12 |
| 13 | DT147 | Otranto (South Adriatic, Italy) | MG981682 | HD13 |
| 14 | DT150 | Otranto (South Adriatic, Italy) | MG981683 | HD14 |
| 15 | DT153 | Otranto (South Adriatic, Italy) | MG981684 | HD14 |
| 16 | DT149 | Otranto (South Adriatic, Italy) | MG981685 | HD15 |
| 17 | DT117 | Alicante (Spain) | MG981686 | HD15 |
| 18 | DT120 | Alicante (Spain) | MG981687 | HD16 |
| 19 | DT124 | Alicante (Spain) | MG981688 | HD17 |
| 20 | DT125 | Alicante (Spain) | MG981689 | HD18 |
| 21 | DT129 | Alicante (Spain) | MG981690 | HD19 |
| 22 | DT168 | Heraklion (North Crete, Greece) | MG981691 | HD20 |
| 23 | DT180 | Heraklion (North Crete, Greece) | MG981692 | HD21 |
| 24 | DT433 | Baleares (Spain) | MG981693 | HD22 |
| 25 | DT439 | Baleares (Spain) | MG981694 | HD23 |
| 26 | DT445 | Baleares (Spain) | MG981695 | HD24 |
| 27 | DT394 | Bastia (Corsica, France) | MG981696 | HD25 |
| 28 | DT397 | Bastia (Corsica, France) | MG981697 | HD26 |
| 29 | DT069 | Galeria (Corsica, France) | MG981698 | HD27 |
| 30 | DT073 | Galeria (Corsica, France) | MG981699 | HD28 |
| 31 | DT055 | St Florent (Corsica, France) | MG981700 | HD29 |
| 32 | DT058 | St Florent (Corsica, France) | MG981701 | HD30 |
| 33 | DT193 | North Aegean Sea (Greece) | MG981702 | HD31 |
| 34 | DT194 | North Aegean Sea (Greece) | MG981703 | HD32 |
| 35 | DT200 | North Aegean Sea (Greece) | MG981704 | HD33 |
| 36 | DT169 | Heraklion (North Crete, Greece) | MG981705 | HD33 |
| 37 | DT457 | Lampedusa (Italy) | MG981706 | HD34 |
| 38 | DT392 | Bastia (Corsica, France) | MG981707 | HD35 |
| 39 | DT458 | Lampedusa (Italy) | MG981708 | HD35 |
| 40 | DT459 | Lampedusa (Italy) | MG981709 | HD36 |
| 41 | DT460 | Lampedusa (Italy) | MG981710 | HD37 |
| 42 | DT001 | Ajaccio (Corsica, France) | MG981711 | HD38 |
| 43 | DT197 | North Aegean Sea (Greece) | MG981712 | HD38 |
| 44 | DT476 | Sardinia (Italy) | MG981713 | HD38 |
| 45 | DT456 | Lampedusa (Italy) | MG981714 | HD39 |
| 46 | DT480 | Sardinia (Italy) | MG981715 | HD39 |
| 47 | DT431 | Baleares (Spain) | MG981716 | HD39 |
| 48 | DT490 | Sardinia (Italy) | MG981717 | HD40 |
| 49 | DT491 | Sardinia (Italy) | MG981718 | HD41 |
| 50 | DT279 | St Raphael (France) | MG981719 | HD42 |
| 51 | DT036 | Ajaccio (Corsica, France) | MG981720 | HD43 |
| 52 | DT059 | St Florent (Corsica, France) | MG981721 | HD43 |
| 53 | DT395 | Bastia (Corsica, France) | MG981722 | HD43 |
| 54 | DT398 | Bastia (Corsica, France) | MG981723 | HD43 |
| 55 | DT172 | Heraklion (North Crete, Greece) | MG981724 | HD43 |
| 56 | DT280 | St Raphael (France) | MG981725 | HD43 |
| 57 | DT283 | St Raphael (France) | MG981726 | HD44 |
| 58 | DT285 | St Raphael (France) | MG981727 | HD45 |
| 59 | DT437 | Baleares (Spain) | MG981728 | HD45 |
| 60 | DT217 | North Tunisia | MG981729 | HD46 |
| 61 | DT070 | Galeria (Corsica, France) | MG981730 | HD47 |
| 62 | DT171 | Heraklion (North Crete, Greece) | MG981731 | HD47 |
| 63 | DT225 | North Tunisia | MG981732 | HD47 |
| 64 | DT281 | St Raphael (France) | MG981733 | HD48 |
| 65 | DT492 | Sardinia (Italy) | MG981734 | HD49 |
| 66 | DT278 | St Raphael (France) | MG981735 | HD50 |
| 67 | DT004 | Bonifacio (Corsica, France) | MG981736 | HD51 |
| 68 | DT007 | Bonifacio (Corsica, France) | MG981737 | HD51 |
| 69 | DT196 | North Aegean Sea (Greece) | MG981738 | HD51 |
| 70 | DT199 | North Aegean Sea (Greece) | MG981739 | HD51 |
| 71 | DT122 | Alicante (Spain) | MG981740 | HD51 |
| 72 | ATL02 | Bay of Biscay (France) | MG981741 | HD51 |
| 73 | ATL03 | Bay of Biscay (France) | MG981742 | HD52 |
| 74 | ATL04 | Bay of Biscay (France) | MG981743 | HD53 |
| 75 | ATL05 | Bay of Biscay (France) | MG981744 | HD54 |
| 76 | ATL06 | Bay of Biscay (France) | MG981745 | HD55 |
| 77 | ATL01 | Bay of Biscay (France) | MG981746 | HD56 |
| 78 | ATL07 | Bay of Biscay (France) | MG981747 | HD56 |

79 ATL08 Bay of Biscay (France) MG981748 HD57
