# Supplementary material for: Absence of spatial genetic structure in common dentex (Dentex dentex Linnaeus, 1758) in the Mediterranean Sea as evidenced by nuclear and mitochondrial molecular markers
Source: PLoS One. 2018 Sep 12;13(9):e0203866. doi: 10.1371/journal.pone.0203866 (PMC6135516; doi:10.1371/journal.pone.0203866)
Supplement: S4 Table — (DOCX) [file pone.0203866.s004.docx]

**Supportive information**

**S4 Table.**

**First part: COI sequences included in mtDNA analyses.**

>HC01

GGCCGGGATAGTCGGGACTGCCTTAAGTCTGCTCATTCGAGCTGAACTTAGCCAACCCGGGGCTCTCC TAGGCGACGACCAGATTTATAATGTTATTGTTACAGCACACGCATTTGTAATAATTTTCTTTATAGTT ATACCAATTATGATTGGAGGTTTTGGAAACTGACTAATTCCGCTAATGATTGGCGCCCCTGATATAGC ATTCCCTCGAATAAACAACATAAGCTTCTGATTGCTCCCTCCATCATTTCTTCTTCTACTCGCCTCCT CAGGGGTTGAGGCCGGGGCTGGCACTGGATGAACAGTCTATCCTCCGCTGGCGGGAAATCTCGCCCAC GCGGGGGCATCCGTTGACCTAACCATTTTTTCTCTTCACTTAGCAGGTATTTCATCAATCCTAGGGGC AATTAATTTTATTACTACAATTATCAATATAAAACCCCCTGCTATTACCCAGTATCAAACTCCCCTAT TCGTCTGGGCCGTCCTTATCACCGCCGTTCTCCTTCTTCTCTCTCTCCCAGTCCTTGCTGCAGGGATT ACAATACTACTCACAGACCGTAACCTAAACACCACCTTCTTCGACCCAGCAGGAGGGGGAGACCCA

>HC02

GGCCGGGATAGTCGGGACTGCCTTAAGTCTGCTCATTCGAGCTGAACTTAGCCAACCCGGGGCTCTCC TAGGCGACGACCAGATTTATAATGTTATTGTTACAGCACACGCATTTGTAATAATTTTCTTTATAGTT ATACCAATTATGATTGGAGGTTTTGGAAACTGACTAATTCCGCTAATGATTGGCGCCCCTGATATAGC ATTCCCTCGAATAAACAACATAAGCTTCTGATTGCTCCCTCCATCATTTCTTCTTCTACTCGCCTCCT CAGGGGTTGAAGCCGGGGCTGGCACTGGATGAACAGTCTATCCTCCGCTGGCGGGAAATCTCGCCCAC GCGGGGGCATCCGTTGACCTAACCATTTTTTCTCTTCACTTAGCAGGTATTTCATCAATCCTAGGGGC AATTAATTTTATTACTACAATTATCAATATAAAACCCCCTGCTATTACCCAGTATCAAACTCCCCTAT TCGTCTGGGCCGTCCTTATCACCGCCGTTCTCCTTCTTCTCTCTCTCCCAGTCCTTGCTGCAGGGATT ACAATACTACTCACAGACCGTAACCTAAACACCACCTTCTTCGACCCAGCAGGAGGGGGAGACCCA

>HC03

GGCCGGGATAGTCGGGACTGCCTTAAGTCTGCTCATTCGAGCTGAACTTAGCCAACCCGGGGCTCTCC TAGGCGACGACCAGATTTATAATGTTATTGTTACAGCACACGCATTTGTAATAATTTTCTTTATAGTT ATACCAATTATGATTGGAGGTTTTGGAAACTGACTAATTCCGCTAATGATTGGCGCCCCTGATATAGC ATTCCCTCGAATAAACAACATAAGCTTCTGATTGCTCCCTCCATCATTCCTTCTTCTACTCGCCTCCT CAGGGGTTGAGGCCGGGGCTGGCACTGGATGAACAGTCTATCCTCCGCTGGCGGGAAATCTCGCCCAC GCGGGGGCATCCGTTGACCTAACCATTTTTTCTCTTCACTTAGCAGGTATTTCATCAATCCTAGGGGC AATTAATTTTATTACTACAATTATCAATATAAAACCCCCTGCTATTACCCAGTATCAAACTCCCCTAT TCGTCTGGGCCGTCCTTATCACCGCCGTTCTCCTTCTTCTCTCTCTCCCAGTCCTTGCTGCAGGGATT ACAATACTACTCACAGACCGTAACCTAAACACCACCTTCTTCGACCCAGCAGGAGGGGGAGACCCA

>HC04

GGCCGGGATAGTCGGGACTGCCTTAAGTCTGCTCATTCGAGCTGAACTTAGCCAACCCGGGGCTCTCC TAGGCGACGACCAGATTTATAATGTTATTGTTACAGCACACGCATTTGTAATAATTTTCTTTATAGTT ATACCAATTATGATTGGAGGTTTTGGAAACTGACTAATTCCGCTAATGATTGGCGCCCCTGATATAGC ATTCCCTCGAATAAACAACATAAGCTTCTGATTGCTCCCTCCATCATTTCTTCTTCTACTCGCCTCCT CAGGGGTTGAGGCCGGGGCTGGCACTGGATGAACAGTCTATCCTCCGCTGGCGGGAAATCTCGCCCAC GCGGGGGCATCCGTTGACCTAACCATTTTTTCTCTTCACTTAGCAGGTATTTCATCAATCCTAGGGGC AATTAATTTTATTACTACAATTATCAATATAAAACCCCCTGCTATTACCCAATATCAAACTCCCTTAT TCGTCTGGGCCGTCCTTATCACCGCCGTTCTCCTTCTTCTCTCTCTCCCAGTCCTTGCTGCAGGGATT ACAATACTACTCACAGACCGTAACCTAAACACCACCTTCTTCGACCCAGCAGGAGGGGGAGACCCA

>HC05

GGCCGGGATAGTCGGGACTGCCTTAAGTCTGCTCATTCGAGCTGAACTTAGCCAACCCGGGGCTCTCC TAGGCGACGACCAGATTTATAATGTTATTGTTACAGCACACGCATTTGTAATAATTTTCTTTATAGTT ATACCAATTATGATTGGAGGTTTTGGAAACTGACTAATTCCGCTAATGATTGGCGCCCCTGATATAGC ATTCCCTCGAATAAACAACATAAGCTTCTGATTGCTCCCTCCATCATTTCTTCTTCTACTCGCCTCCT CAGGGGTTGAGGCCGGGGCTGGCACTGGATGAACAGTCTATCCTCCGCTGGCGGGAAATCTCGCCCAC GCGGGGGCATCCGTTGACCTAACCATTTTTTCTCTTCACTTGGCAGGTATTTCATCAATCCTAGGGGC AATTAATTTTATTACTACAATTATCAATATAAAACCCCCTGCTATTACCCAGTATCAAACTCCCCTAT TCGTCTGGGCCGTCCTTATCACCGCCGTTCTCCTTCTTCTCTCTCTCCCAGTCCTTGCTGCAGGGATT ACAATACTACTCACAGACCGTAACCTAAACACCACCTTCTTCGACCCAGCAGGAGGGGGAGACCCA

>HC06

GGCCGGGATAGTCGGGACTGCCTTAAGTCTGCTCATTCGAGCTGAACTTAGCCAACCCGGGGCTCTCC TAGGCGACGACCAGATTTATAATGTTATTGTTACAGCACACGCATTTGTAATAATTTTCTTTATAGTT ATACCAATTATGATTGGAGGTTTTGGAAACTGACTAATTCCGCTAATGATTGGCGCCCCTGATATAGCATTCCCTCGAATAAACAACATAAGCTTCTGATTGCTCCCTCCCTCATTTCTTCTTCTACTCGCCTCCT CAGGGGTTGAGGCCGGGGCTGGCACTGGATGAACAGTCTATCCTCCGCTGGCGGGAAATCTCGCCCAC GCGGGGGCATCCGTTGACCTAACCATTTTTTCTCTTCACTTAGCAGGTATTTCATCAATCCTAGGGGC AATTAATTTTATTACTACAATTATCAACATAAAACCCCCTGCTATTACCCAGTATCAAACTCCCCTAT TCGTCTGGGCCGTCCTTATCACCGCCGTTCTCCTTCTTCTCTCTCTCCCAGTCCTTGCTGCAGGGATT ACAATACTACTCACAGACCGTAACCTAAACACCACCTTCTTCGACCCAGCAGGAGGGGGAGACCCA

>HC07

GGCCGGGATAGTCGGGACTGCCTTAAGTCTGCTCATTCGAGCTGAACTTAGCCAACCCGGGGCTCTCC TAGGCGACGACCAGATTTATAATGTTATTGTTACAGCACACGCATTTGTAATAATTTTCTTTATAGTT ATACCAATTATGATTGGAGGTTTTGGAAACTGACTAATTCCGCTAATGATTGGCGCCCCTGATATAGC ATTCCCTCGAATAAACAACATAAGCTTCTGATTGCTCCCTCCATCATTTCTTCTTCTACTCGCCTCCT CAGGGGTTGAGGCCGGGGCTGGCACTGGATGAACAGTCTATCCTCCGCTGGCGGGAAATCTCGCCCAC GCGGGGGCATCCGTTGACCTAACCATTTTTTCTCTTCACTTAGCAGGTATTTCATCAATCCTAGGGGC AATTAATTTTATTACTACAATTATCAATATAAAACCCCCTGCTATTACCCAGTATCAAACCCCCCTAT TCGTCTGAGCCGTCCTTATCACCGCCGTTCTCCTTCTTCTCTCTCTCCCAGTCCTTGCTGCAGGGATT ACAATACTACTCACAGACCGTAACCTAAACACCACCTTCTTCGACCCAGCAGGAGGGGGAGACCCA

>HC08

GGCCGGGATAGTCGGGACTGCCTTAAGTCTGCTCATTCGAGCTGAACTTAGCCAACCCGGGGCTCTCC TAGGCGACGACCAGATTTATAATGTTATTGTTACAGCACACGCATTTGTAATAATTTTCTTTATAGTT ATACCAATTATAATTGGAGGTTTTGGAAACTGACTAATTCCGCTAATGATTGGCGCCCCTGATATAGC ATTCCCTCGAATAAACAACATAAGCTTCTGATTGCTCCCTCCATCATTTCTTCTTCTACTCGCCTCCT CAGGGGTTGAGGCCGGGGCTGGCACTGGATGAACAGTCTATCCTCCGCTGGCGGGAAATCTCGCCCAC GCGGGGGCATCCGTTGACCTAACCATTTTTTCTCTTCACTTAGCAGGTATTTCATCAATCCTAGGGGC AATTAATTTTATTACTACAATTATCAATATAAAACCCCCTGCTATTACCCAGTATCAAACTCCCCTAT TCGTCTGGGCCGTCCTTATCACCGCCGTTCTCCTTCTTCTCTCTCTCCCAGTCCTTGCTGCAGGGATT ACAATACTACTCACAGACCGTAACCTAAACACCACCTTCTTCGACCCAGCAGGAGGGGGAGACCCA

>HC09

GGCCGGGATAGTCGGGACTGCCTTAAGTCTGCTCATTCGGGCTGAACTTAGCCAACCCGGGGCTCTCC TAGGCGACGACCAGATTTATAATGTTATTGTTACAGCACACGCATTTGTAATAATTTTCTTTATAGTT ATACCAATTATGATTGGAGGTTTTGGAAACTGACTAATTCCGCTAATGATTGGCGCCCCTGATATAGC ATTCCCTCGAATAAACAACATAAGCTTCTGATTGCTCCCTCCATCATTTCTTCTTCTACTCGCCTCCT CAGGGGTTGAGGCCGGGGCTGGCACTGGATGAACAGTCTATCCTCCGCTGGCGGGAAATCTCGCCCAC GCGGGGGCATCCGTTGACCTAACCATTTTTTCTCTTCACTTAGCAGGTATTTCATCAATCCTAGGGGC AATTAATTTTATTACTACAATTATCAATATAAAACCCCCTGCTATTACCCAGTATCAAACTCCCCTAT TCGTCTGGGCCGTCCTTATCACCGCCGTTCTCCTTCTTCTCTCTCTCCCAGTCCTTGCTGCAGGGATT ACAATACTACTCACAGACCGTAACCTAAACACCACCTTCTTCGACCCAGCAGGAGGGGGAGACCCA

>HC10

GGCCGGGATAGTCGGGACTGCCTTAAGTCTGCTCATTCGAGCTGAACTTAGCCAACCCGGGGCTCTCC TAGGCGACGACCAGATTTATAATGTTATTGTTACAGCACACGCATTTGTAATAATTTTCTTTATAGTT ATACCAATTATGATTGGAGGTTTTGGAAACTGACTAATTCCGCTAATGATCGGCGCCCCTGATATAGC ATTCCCTCGAATAAACAACATAAGCTTCTGATTGCTCCCTCCATCATTTCTTCTTCTACTCGCCTCCT CAGGGGTTGAGGCCGGGGCTGGCACTGGATGAACAGTCTATCCTCCGCTGGCGGGAAATCTCGCCCAC GCGGGGGCATCCGTTGACCTAACCATTTTTTCTCTTCACTTAGCAGGTATTTCATCAATCCTAGGGGC AATTAATTTTATTACTACAATTATCAATATAAAACCCCCTGCTATTACCCAGTATCAAACTCCCCTAT TCGTCTGGGCCGTCCTTATCACCGCCGTTCTCCTTCTTCTCTCTCTCCCAGTCCTTGCTGCAGGGATT ACAATACTACTCACAGACCGTAACCTAAACACCACCTTCTTCGACCCAGCAGGAGGGGGAGACCCA

>HC11

GGCCGGGATAGTCGGGACTGCCTTAAGTCTGCTCATTCGAGCTGAACTTAGCCAACCCGGGGCTCTCC TAGGCGACGACCAGATTTATAATGTTATTGTTACAGCACACGCATTTGTAATAATTTTCTTTATAGTT ATACCAATTATGATTGGAGGTTTTGGAAACTGACTAATTCCGCTAATGATTGGCGCCCCTGATATAGC ATTCCCTCGAATAAACAACATAAGCTTCTGATTGCTCCCTCCATCATTTCTTCTTCTACTCGCCTCCT CAGGGGTTGAGGCCGGGGCTGGCACTGGATGAACAGTCTATCCTCCGCTGGCGGGAAATCTCGCCCAC GCGGGGGCATCCGTTGACCTAACCATTTTTTCTCTTCACTTAGCAGGTATTTCATCAATCCTAGGGGC AATTAATTTTATTACTACAATTATCAATATAAAACCCCCTGCTATTACCCAGTATCAAACTCCCCTAT

TCGTCTGGGCCGTCCTTATCACCGCCGTTCTCCTTCTTCTCTCTCTCCCAGTCCTTGCTGCAGGGATT ACGATACTACTCACAGACCGTAACCTAAACACCACCTTCTTCGACCCAGCAGGAGGGGGAGACCCA

>HC12

GGCCGGGATAGTCGGGACTGCCTTAAGTCTGCTCATTCGAGCTGAACTTAGCCAACCCGGGGCTCTCC TAGGCGACGACCAGATTTATAATGTTATTGTTACAGCACACGCATTTGTAATAATTTTCTTTATAGTT ATACCAATTATGATTGGAGGTTTTGGAAACTGACTAATTCCGCTAATGATTGGCGCCCCTGATATAGC ATTCCCTCGAATAAACAACATAAGCTTCTGATTGCTCCCTCCATCATTTCTTCTTCTACTCGCCTCCT CAGGGGTTGAGGCCGGGGCTGGCACTGGATGAACAGTCTATCCTCCGCTGGCGGGAAATCTCGCCCAC GCGGGGGCATCCGTTGACCTAACCATTTTTTCTCTTCACTTAGCAGGTATTTCATCAATTCTAGGGGC AATTAATTTTATTACTACAATTATCAATATAAAACCCCCTGCTATTACCCAGTATCAAACTCCCCTAT TCGTCTGGGCCGTCCTTATCACCGCCGTTCTCCTTCTTCTCTCTCTCCCAGTCCTTGCTGCAGGGATT ACAATACTACTCACAGACCGTAACCTAAACACCACCTTCTTCGACCCAGCAGGAGGGGGAGACCCA

>HC13

GGCCGGGATAGTCGGGACTGCCTTAAGTCTGCTCATTCGAGCTGAACTTAGCCAACCCGGGGCTCTCC TAGGCGACGACCAGATTTATAATGTTATTGTTACAGCACACGCATTTGTAATAATTTTCTTTATAGTT ATACCAATTATGATTGGAGGTTTTGGAAACTGACTAATTCCGCTAATGATTGGCGCCCCTGATATAGC ATTCCCTCGAATAAACAACATAAGCTTCTGATTGCTCCCTCCATCATTTCTTCTTCTACTCGCCTCCT CAGGGGTTGAGGCCGGGGCTGGCACTGGATGAACAGTCTATCCTCCGCTGGCGGGAAATCTCGCCCAC GCGGGGGCATCCGTTGACCTAACCATTTTTTCTCTTCACTTAGCAGGTATTTCATCAATCCTAGGGGC AATTAATTTTATTACTACAATTATCAATATAAAACCCCCTGCTATTACCCAGTATCAAACTCCCCTAT TCGTCTGGGCCGTCCTCATCACCGCCGTTCTCCTTCTTCTCTCTCTCCCAGTCCTTGCTGCAGGGATT ACAATACTACTCACAGACCGTAACCTAAACACCACCTTCTTCGACCCAGCAGGAGGGGGAGACCCA

>HC14

GGCCGGGATAGTCGGGACTGCCTTAAGTCTGCTCATTCGAGCTGAACTTAGCCAACCCGGGGCTCTCC TAGGCGACGACCAGATTTATAATGTTATTGTTACAGCACACGCATTTGTAATAATTTTCTTTATAGTT ATACCAATTATGATTGGAGGTTTTGGAAACTGACTAATTCCGCTAATGATTGGCGCCCCTGATATGGC ATTCCCTCGAATAAACAACATAAGCTTCTGATTGCTCCCTCCATCATTTCTTCTTCTACTCGCCTCCT CAGGGGTTGAGGCCGGGGCTGGCACTGGATGAACAGTCTATCCTCCGCTGGCGGGAAATCTCGCCCAC GCGGGGGCATCCGTTGACCTAACCATTTTTTCTCTTCACTTAGCAGGTATTTCATCAATCCTAGGGGC AATTAATTTTATTACTACAATTATCAATATAAAACCCCCTGCTATTACCCAGTATCAAACTCCCCTAT TCGTCTGGGCCGTCCTTATCACCGCCGTTCTCCTTCTTCTCTCTCTCCCAGTCCTTGCTGCAGGGATT ACAATACTACTCACAGACCGTAACCTAAACACCACCTTCTTCGACCCAGCAGGAGGGGGAGACCCA

>HC15

GGCCGGGATAGTCGGGACAGCCTTAAGTCTGCTCATTCGAGCTGAACTTAGCCAACCCGGGGCTCTCC TAGGCGACGACCAGATTTATAATGTTATTGTTACAGCACACGCATTTGTAATAATTTTCTTTATAGTT ATACCAATTATGATTGGAGGTTTTGGAAACTGACTAATCCCGCTAATGATTGGCGCCCCTGATATAGC ATTCCCTCGAATAAACAACATAAGCTTCTGATTGCTCCCTCCATCATTTCTTCTTCTACTCGCCTCCT CAGGGGTTGAGGCCGGGGCTGGCACTGGATGAACAGTCTATCCTCCGCTGGCGGGAAATCTCGCCCAC GCGGGGGCATCCGTTGACCTAACCATTTTTTCTCTTCACTTAGCAGGTATTTCATCAATCCTAGGGGC AATTAATTTTATTACTACAATTATCAATATAAAACCCCCTGCTATTACCCAGTATCAAACTCCCCTAT TCGTCTGGGCCGTCCTTATCACCGCCGTTCTCCTTCTTCTCTCTCTCCCAGTCCTTGCTGCAGGGATT ACAATACTACTCACAGACCGTAACCTAAACACCACCTTCTTCGACCCAGCAGGAGGGGGAGACCCA

>HC16

GGCCGGGATAGTCGGGACTGCCTTAAGTCTGCTCATTCGAGCTGAACTTAGCCAACCCGGGGCTCTCC TTGGCGACGACCAGATTTATAATGTTATTGTTACAGCACACGCATTTGTAATAATTTTCTTTATAGTA ATACCAATTATGATTGGAGGTTTTGGAAACTGACTAATTCCGCTAATGATTGGCGCCCCTGATATAGC ATTCCCTCGAATAAACAATATAAGCTTCTGATTGCTCCCTCCATCATTTCTTCTTCTACTCGCCTCCT CAGGGGTTGAGGCCGGGGCTGGCACTGGATGAACAGTCTATCCGCCGCTGGCGGGAAATCTCGCCCAC GCGGGGGCATCCGTTGACCTAACCATTTTTTCTCTTCACTTAGCAGGTATTTCATCAATCCTAGGGGC AATTAATTTTATTACTACAATTATCAATATAAAACCCCCTGCTATTACCCAGTATCAAACTCCCCTAT TCGTCTGGGCCGTCCTTATCACCGCCGTTCTCCTTCTTCTCTCTCTCCCAGTCCTTGCTGCAGGGATT ACAATACTACTCACAGACCGTAACCTAAACACCACCTTCTTCGACCCAGCAGGAGGGGGAGACCCA

>HC17

GGCCGGGATAGTCGGGACTGCCTTAAGTCTGCTCATTCGAGCTGAACTTAGCCAACCCGGGGCTCTCC

TTGGCGACGACCAGATTTATAATGTTATTGTTACAGCACACGCATTTGTAATAATTTTCTTTATAGTT ATACCAATTATGATTGGAGGTTTTGGAAACTGACTAATCCCGCTAATGATTGGCGCCCCTGATATAGC ATTCCCTCGAATAAACAACATAAGCTTCTGATTGCTCCCTCCATCATTTCTTCTTCTACTCGCCTCCT CAGGGGTTGAGGCCGGGGCTGGCACTGGATGAACAGTCTATCCTCCGCTGGCGGGAAATCTCGCCCAC GCGGGGGCATCCGTTGACCTAACCATTTTTTCTCTTCACTTAGCAGGTATTTCATCAATCCTAGGGGC AATTAATTTTATTACTACAATTATCAATATAAAACCCCCTGCTATTACCCAGTATCAAACTCCCCTAT TCGTCTGGGCCGTCCTAATCACCGCCGTTCTCCTTCTTCTCTCTCTCCCAGTCCTTGCTGCAGGGATT ACAATACTACTCACAGACCGTAACCTAAACACCACCTTCTTCGACCCAGCAGGAGGGGGAGACCCA

>HC18

GGCCGGGATAGTCGGGACAGCCTTAAGTCTGCTCATTCGAGCTGAACTTAGCCAACCCGGGGCTCTCC TAGGCGACGACCAGATTTATAATGTTATTGTTACAGCACACGCATTTGTAATAATTTTCTTTATAGTA ATACCAATTATGATTGGAGGTTTTGGAAACTGACTAATTCCGCTAATGATTGGCGCCCCTGATATAGC ATTCCCTCGAATAAACAACATAAGCTTCTGATTGCTCCCTCCATCATTTCTTCTTCTACTCGCCTCCT CAGGAGTTGAGGCCGGGGCTGGCACTGGATGAACAGTCTATCCTCCGCTGGCGGGAAATCTCGCCCAC GCGGGGGCATCCGTTGACCTAACCATTTTTTCTCTTCACTTAGCAGGTATTTCATCAATCCTAGGGGC AATTAATTTTATTACTACAATTATCAATATAAAACCCCCTGCTATTACCCAGTATCAAACTCCCCTAT TCGTCTGGGCCGTCCTTATCACCGCCGTTCTCCTTCTTCTCTCTCTCCCAGTCCTTGCTGCAGGGATT ACAATACTACTCACAGACCGTAACCTAAACACCACCTTCTTCGACCCAGCAGGAGGGGGAGACCCA

>HC19

GGCCGGGATAGTCGGGACTGCCTTAAGTCTGCTCATTCGAGCTGAACTTAGCCAACCCGGGGCTCTCC TAGGCGACGACCAGATTTATAATGTTATTGTTACAGCACACGCATTTGTAATAATTTTCTTTATAGTT ATACCAATTATGATTGGAGGTTTTGGAAACTGACTAATTCCGCTAATGATTGGCGCCCCTGATATAGC ATTCCCTCGAATAAACAACATAAGCTTCTGATTGCTCCCTCCATCATTTCTTCTTCTACTCGCCTCCT CAGGGGTTGAGGCCGGGGCTGGCACTGGATGAACAGTCTATCCTCCGCTGGCGGGAAATCTCGCCCAC GCGGGGGCATCCGTTGACCTAACCATTTTTTCTCTTCACTTAGCAGGTATTTCATCAATCCTAGGGGC AATTAATTTTATTACTACAATTATCAATATAAAACCCCCTGCTATTACCCAGTATCAAACTCCCCTAT TCGTCTGGGCCGTCCTTATCACCGCCGTTCTCCTTCTTCTCTCTCTCCCAGTCCTTGCTGCAGGGATT ACAATACTACTCACCGACCGTAACCTAAACACCACCTTCTTCGACCCAGCAGGAGGGGGAGACCCA

>HC20

GGCCGGGATAGTCGGGACTGCCTTAAGTCTGCTCATTCGAGCTGAACTTAGCCAACCCGGGGCTCTCC TAGGCGACGACCAGATTTATAATGTTATTGTTACAGCACACGCATTTGTAATAATTTTCTTTATAGTT ATACCAATTATGATTGGAGGTTTTGGAAACTGACTAATTCCGCTAATGATTGGCGCCCCTGATATAGC ATTCCCTCGAATAAACAACATAAGCTTCTGATTGCTCCCTCCATCATTTCTTCTTCTACTCGCCTCCT CAGGGGTTGAGGCCGGGGCTGGCACTGGATGAACAGTCTATCCTCCGCTGGCGGGAAATCTCGCCCAC GCGGGGGCATCCGTTGACCTAACCATTTTTTCTCTTCACTTAGCAGGTATTTCATCAATCCTAGGGGC AATTAATTTTATTACTACAATTATCAATATAAAACCCCCTGCTATTACCCAGTATCAAACTCCCCTAT TCGTCTGGGCCGTCCTTATCACCGCCGTTCTCCTTCTTCTCTCTCTCCCAGTCCTTGCTGCAGGGATT ACAATACTAATCACAGACCGTAACCTAAACACCACCTTCTTCGACCCAGCAGGAGGGGGAGACCCA

>HC21

GGCCGGGATAGTAGGGACTGCCTTAAGTCTACTCATTCGAGCTGAACTTAGCCAACCCGGAGCTCTTC TAGGCGACGACCAGATTTATAATGTTATTGTTACAGCACACGCATTTGTAATAATTTTCTTTATAGTT ATGCCAATTATGATCGGGGGATTTGGAAACTGATTAATTCCACTCATGATCGGTGCCCCTGACATAGC ATTTCCCCGAATAAACAACATGAGCTTCTGACTACTCCCTCCGTCATTCCTCCTTCTACTCGCTTCCT CAGGGGTTGAAGCCGGAGCTGGCACTGGATGGACAGTTTACCCGCCACTGGCAGGAAATCTCGCCCAT GCAGGAGCATCAGTCGACCTAACCATTTTTTCTCTTCACTTAGCAGGTATTTCATCAATTCTTGGGGC AATTAATTTTATTACTACCATTATTAACATAAAGCCTCCTGCTATCACCCAGTATCAGACCCCTCTGT TCGTCTGAGCCGTCCTTATTACCGCGGTTCTCCTCCTTCTCTCACTACCAGTTCTTGCTGCGGGAATT ACAATGCTACTTACGGACCGTAACCTAAACACCACCTTCTTCGACCCAGCAGGAGGAGGAGACCCA

>HC22

GGCCGGGATAGTAGGGACTGCCTTAAGTCTACTCATTCGAGCTGAACTTAGCCAACCCGGAGCTCTTC TAGGCGACGACCAGATTTATAATGTTATTGTTACAGCACACGCATTTGTAATAATTTTCTTTATAGTT ATGCCAATTATGATCGGGGGGTTTGGAAACTGATTAATTCCACTCATGATCGGTGCCCCTGACATAGC ATTTCCCCGAATAAACAACATGAGCTTCTGACTACTCCCTCCGTCATTCCTCCTTCTACTCGCTTCCT CAGGGGTTGAAGCCGGAGCTGGCACTGGATGGACAGTTTACCCGCCACTGGCAGGAAATCTCGCCCAT

GCAGGAGCATCAGTCGACCTAACCATTTTTTCTCTTCACTTAGCAGGTATTTCATCAATTCTTGGGGC AATTAATTTTATTACTACCATTATTAACATAAAGCCTCCTGCTATCACCCAGTATCAGACCCCTCTGT TCGTCTGAGCCGTCCTTATTACCGCGGTTCTCCTCCTTCTCTCACTACCAGTTCTTGCTGCGGGAATT ACAATGCTACTTACGGACCGTAACCTAAACACCACCTTCTTCGACCCAGCAGGAGGAGGAGACCCA

>HC23

GGCCGGGATAGTAGGGACTGCCTTAAGTCTACTCATTCGAGCTGAACTTAGCCAACCCGGAGCTCTTC TAGGCGACGACCAGATTTATAATGTTATTGTTACAGCACACGCATTTGTAATAATTTTCTTTATAGTT ATGCCAATTATGATCGGGGGATTTGGAAACTGATTAATTCCACTCATGATCGGTGCCCCTGACATAGC ATTTCCCCGAATAAACAACATGAGCTTCTGACTACTCCCTCCGTCATTCCTCCTTCTACTCGCTTCCT CAGGGGTTGAAGCCGGAGCTGGCACTGGATGGACAGTTTACCCACCACTGGCAGGAAATCTCGCCCAT GCAGGAGCATCAGTCGACCTAACCATTTTTTCTCTTCACTTAGCAGGTATTTCATCAATTCTTGGAGC AATTAATTTTATTACTACCATTATTAACATAAAGCCTCCTGCTATTACCCAGTATCAGACCCCTCTGT TCGTCTGAGCCGTCCTTATTACCGCGGTTCTCCTCCTTCTCTCACTACCAGTTCTTGCTGCGGGAATT ACAATGCTACTTACGGACCGTAACCTAAACACCACCTTCTTCGACCCAGCAGGAGGAGGAGACCCA

>HC24

GGCCGGGATAGTAGGGACTGCCTTAAGTCTACTCATTCGAGCTGAACTTAGCCAACCCGGAGCTCTTC TAGGCGACGACCAGATTTATAATGTTATTGTTACAGCACACGCATTTGTAATAATTTTCTTTATAGTT ATGCCAATTATGATCGGGGGATTTGGAAACTGATTAATTCCACTCATGATCGGTGCCCCTGACATAGC ATTCCCCCGAATAAACAACATGAGCTTCTGACTACTCCCTCCGTCATTCCTCCTTCTACTCGCTTCCT CAGGGGTTGAAGCCGGAGCTGGCACTGGATGGACAGTTTACCCGCCACTGGCAGGAAATCTCGCCCAT GCAGGAGCATCAGTCGACTTAACCATTTTTTCTCTTCACTTAGCAGGTATTTCATCAATTCTTGGGGC AATTAATTTTATTACTACCATTATTAACATAAAGCCTCCTGCTATCACCCAGTATCAGACCCCTCTGT TCGTCTGAGCCGTCCTTATTACCGCGGTTCTCCTCCTTCTCTCACTACCAGTTCTTGCTGCGGGGATT ACAATGCTACTTACGGACCGTAACCTAAACACCACCTTCTTCGACCCAGCAGGAGGAGGAGACCCA

>HC25

GGCCGGGATAGTAGGGACTGCCTTAAGTCTACTCATTCGAGCTGAACTTAGCCAACCCGGAGCTCTTC TAGGCGACGACCAGATTTATAATGTTATTGTTACAGCACACGCATTTGTAATAATTTTCTTTATAGTT ATGCCAATTATGATCGGGGGATTTGGAAACTGATTAATTCCACTCATGATCGGTGCCCCTGACATAGC ATTTCCCCGAATAAACAACATGAGCTTCTGACTACTCCCTCCGTCATTCCTCCTTCTACTCGCTTCCT CAGGGGTTGAAGCCGGAGCTGGCACTGGATGGACAGTTTACCCGCCACTGGCAGGAAATCTCGCCCAT GCAGGAGCATCAGTCGACCTAACCATTTTTTCTCTTCACTTAGCAGGTATTTCATCAATTCTTGGGGC AATTAATTTTATTACTACCATTATTAACATAAAGCCTCCTGCTATTACCCAGTATCAGACCCCTCTGT TCGTCTGAGCCGTCCTTATTACCGCGGTTCTCCTCCTTCTCTCACTACCAGTTCTTGCTGCGGGAATT ACAATGCTACTTACGGACCGTAACCTAAACACCACCTTCTTCGACCCAGCAGGAGGAGGAGACCCA

>HC26

GGCCGGGATAGTAGGGACTGCCCTAAGTCTACTCATTCGAGCTGAACTTAGCCAACCCGGAGCTCTTC TAGGCGACGACCAGATTTATAATGTTATTGTTACAGCACACGCATTTGTAATAATTTTCTTTATAGTT ATGCCAATTATGATCGGGGGATTTGGAAACTGATTAATTCCACTCATGATCGGTGCCCCTGACATAGC ATTTCCCCGAATAAACAACATGAGCTTCTGACTACTCCCTCCGTCATTCCTCCTTCTACTCGCTTCCT CAGGGGTTGAAGCCGGAGCTGGCACTGGATGGACAGTTTACCCGCCACTGGCAGGAAATCTCGCCCAT GCAGGAGCATCAGTCGACCTAACCATTTTTTCTCTTCACTTAGCAGGTATTTCATCAATTCTTGGGGC AATTAATTTTATTACTACCATTATTAACATAAAGCCTCCTGCTATCACCCAGTATCAGACCCCTCTGT TCGTCTGAGCCGTCCTTATTACCGCGGTTCTCCTCCTTCTCTCACTACCAGTTCTTGCTGCGGGAATT ACAATGCTACTTACGGACCGTAACCTAAACACCACCTTCTTCGACCCAGCAGGAGGAGGAGACCCA

>HC27

GGCCGGGATAGTAGGGACTGCCTTAAGTCTACTCATTCGAGCTGAACTTAGCCAACCCGGAGCTCTTC TAGGCGACGACCAGATTTATAATGTTATTGTTACAGCACACGCATTTGTAATAATTTTCTTTATAGTT ATGCCAATTATGATCGGGGGGTTTGGAAACTGATTAATTCCACTCATGATCGGTGCCCCTGACATAGC ATTTCCCCGAATAAACAACATGAGCTTCTGACTACTCCCTCCGTCATTCCTCCTTCTACTCGCTTCCT CAGGGGTTGAAGCCGGAGCTGGCACTGGATGGACAGTTTACCCGCCACTGGCAGGAAATCTCGCCCAT GCAGGAGCATCAGTCGACCTAACCATTTTTTCTCTTCACTTAGCAGGTATTTCATCAATTCTTGGGGC AATTAATTTTATTACTACCATTATTAACATAAAGCCTCCTGCTATCACCCAGTATCAGACCCCTCTGT TCGTCTGAGCCGTCCTTATTACCGCGGTTCTCCTCCTTCTCTCACTACCAGTTCTTGCTGCGGGAATT ACAATGCTACTTACGGACCGCAACCTAAACACCACCTTCTTCGACCCAGCAGGAGGAGGAGACCCA

>HC28

GGCCGGGATAGTAGGAACTGCCCTAAGTCTACTCATTCGAGCTGAACTTAGCCAACCCGGAGCTCTTC TAGGCGACGACCAGATTTATAATGTTATTGTTACAGCACACGCATTTGTAATAATTTTCTTTATAGTT ATACCAATTATAATCGGGGGATTTGGGAACTGATTAATTCCACTTATGATCGGAGCCCCTGACATAGC ATTCCCTCGGATGAACAACATAAGCTTCTGACTGCTCCCTCCGTCATTTCTTCTTCTACTCGCCTCTT CAGGGGTTGAGGCCGGAGCTGGCACTGGATGAACCGTTTACCCGCCGCTGGCGGGGAATCTTGCTCAC GCAGGAGCATCAGTAGACCTGACCATTTTTTCTCTTCACTTAGCAGGTATTTCATCAATTCTCGGAGC AATTAACTTTATTACTACCATTATTAACATAAAACCCCCCGCTATTACTCAATATCAAACTCCTTTAT TCGTTTGAGCCGTCCTTATTACCGCTGTCCTCCTCCTCCTCTCCCTTCCAGTTCTTGCTGCAGGAATT ACCATACTGCTTACGGACCGTAACCTAAACACCACCTTCTTCGACCCGGCAGGGGGTGGAGACCCA

>HC29

GGCCGGGATAGTAGGAACTGCCCTAAGTCTACTCATTCGAGCTGAACTTAGCCAACCCGGAGCTCTTC TAGGCGACGACCAGATTTATAATGTTATTGTTACAGCACACGCATTTGTAATAATTTTCTTTATAGTT ATACCAATTATAATCGGGGGATTTGGGAACTGATTAATTCCACTTATGATCGGAGCCCCTGACATAGC ATTCCCTCGGATGAACAACATAAGCTTCTGACTGCTCCCTCCATCATTTCTTCTTCTACTCGCCTCTT CAGGAGTTGAGGCCGGAGCTGGCACTGGATGAACCGTTTACCCGCCGCTGGCGGGGAATCTTGCTCAC GCAGGAGCATCAGTAGACCTGACCATTTTTTCTCTTCACTTAGCAGGTATTTCATCAATTCTCGGAGC AATTAACTTTATTACTACCATTATTAACATAAAACCCCCCGCTATTACTCAATATCAAACTCCTTTAT TCGTTTGAGCCGTCCTTATTACCGCTGTCCTCCTCCTCCTCTCCCTTCCAGTTCTTGCTGCAGGAATT ACCATGCTGCTTACGGACCGTAACCTAAACACCACCTTCTTCGACCCGGCAGGGGGTGGAGACCCA

>HC30

GGCCGGGATAGTGGGGACAGCCCTAAGCCTGCTCATTCGAGCTGAACTTAGCCAACCCGGTGCTCTCC TAGGCGACGACCAGATTTATAATGTTATTGTTACAGCACATGCATTTGTAATAATTTTCTTTATAGTA ATACCAATTATGATTGGAGGCTTTGGGAATTGATTAATTCCACTTATGATCGGCGCTCCTGATATAGC ATTCCCCCGAATAAACAACATAAGCTTCTGACTGCTTCCCCCCTCATTTCTTCTTCTACTTGCCTCCT CAGGGGTCGAAGCTGGGGCCGGCACTGGATGAACGGTATACCCCCCACTAGCAGGAAATCTTGCCCAC GCAGGAGCATCCGTCGACCTAACCATCTTCTCCCTTCACTTAGCTGGAATTTCATCAATTCTTGGTGC AATTAATTTTATTACTACCATTATTAACATGAAACCTCCCGCTATTACCCAGTATCAAACCCCGCTGT TTGTATGGGCCGTTCTTATTACGGCCGTGCTTCTTCTTTTATCGCTGCCAGTTCTTGCTGCGGGAATT ACAATGCTTCTTACGGACCGTAACCTAAACACCACTTTCTTTGACCCAGCAGGGGGAGGGGACCCT

>HC31

GGCCGGGATAGTAGGGACTGCCCTAAGTCTACTCATTCGAGCGGAACTTAGCCAACCCGGAGCTCTCC TAGGCGACGACCAGATTTATAATGTTATTGTTACAGCACACGCATTTGTAATAATTTTCTTTATAGTT ATACCAATTATAATTGGGGGATTTGGGAACTGATTGATTCCACTAATGATTGGAGCCCCTGACATAGC ATTCCCTCGGATGAACAACATAAGCTTCTGACTGCTCCCTCCGTCATTTCTTCTTTTACTCGCCTCTT CAGGCGTTGAGGCCGGAGCTGGCACTGGATGAACAGTTTACCCGCCGCTGGCAGGAAATCTCGCTCAC GCAGGAGCATCAGTCGACCTAACCATTTTCTCTCTTCACTTAGCAGGTATTTCATCGATTCTTGGGGC AATTAACTTTATCACTACCATTATCAACATGAAACCCCCTGCTATTACTCAGTATCAAACTCCCTTAT TCGTTTGAGCCGTCCTTATTACCGCTGTCCTCCTCCTCCTCTCCCTTCCAGTTCTTGCTGCGGGAATT ACCATGCTGCTCACGGACCGTAACCTAAACACCACCTTCTTCGATCCGGCAGGGGGTGGGGACCCA

>HC32

GGCCGGGATAGTAGGGACTGCCCTAAGTCTACTCATTCGAGCGGAACTTAGCCAACCCGGAGCTCTCC TAGGCGACGACCAGATTTATAATGTTATTGTTACAGCACACGCATTTGTAATAATTTTCTTTATAGTT ATACCAATTATAATTGGGGGATTTGGGAACTGATTGATTCCACTAATGATTGGAGCCCCTGACATAGC ATTCCCTCGGATGAACAACATAAGCTTCTGACTGCTCCCTCCGTCATTTCTTCTTTTACTCGCCTCTT CAGGCGTTGAGGCCGGAGCTGGCACTGGATGAACAGTTTACCCGCCGCTGGCAGGAAATCTCGCTCAC GCAGGAGCATCAGTCGACCTAACCATTTTCTCGCTTCACTTAGCAGGTATTTCATCGATTCTTGGGGC AATTAACTTTATCACTACCATTATCAACATGAAACCCCCTGCTATTACTCAGTATCAAACTCCCTTAT TCGTTTGAGCCGTCCTTATTACCGCTGTCCTCCTCCTCCTCTCCCTTCCAGTTCTTGCTGCGGGAATT ACCATGCTGCTCACGGACCGTAACCTAAACACCACCTTCTTCGATCCGGCAGGGGGTGGGGACCCA

>HC33

GGCCGGGATAGTAGGGACTGCCCTAAGTCTACTCATTCGAGCGGAACTTAGCCAACCCGGAGCTCTCC TAGGCGACGACCAGATTTATAATGTTATTGTTACAGCACACGCATTTGTAATAATTTTCTTTATAGTT ATACCAATTATAATCGGGGGATTTGGGAACTGATTGATTCCACTAATGATCGGAGCCCCTGACATAGC

ATTCCCTCGGATGAACAACATAAGCTTCTGACTGCTCCCTCCATCATTTCTTCTTTTACTCGCCTCTT CAGGCGTTGAGGCCGGAGCTGGCACTGGATGAACAGTTTACCCGCCGCTGGCAGGAAATCTCGCTCAC GCAGGAGCATCAGTCGACCTAACCATTTTCTCTCTTCACTTAGCAGGTATTTCATCGATTCTTGGGGC AATTAACTTTATCACTACCATTATCAACATGAAACCCCCTGCTATTACTCAGTATCAAACTCCCTTAT TCGTTTGAGCCGTCCTTATTACCGCTGTCCTCCTCCTCCTCTCCCTTCCAGTTCTTGCTGCGGGAATT ACCATGCTGCTCACGGACCGTAACCTAAACACCACCTTCTTCGATCCGGCAGGGGGTGGGGACCCA

>HC34

GGCCGGGATAGTAGGGACTGCCCTAAGTCTACTCATTCGAGCGGAACTTAGCCAACCCGGAGCTCTCC TAGGCGACGACCAGATTTATAATGTTATTGTTACAGCACACGCATTTGTAATAATTTTCTTTATAGTT ATACCAATTATAATTGGGGGATTTGGGAACTGATTGATTCCACTAATGATTGGAGCCCCTGACATAGC ATTCCCTCGGATGAACAACATAAGCTTCTGACTGCTCCCTCCGTCATTTCTTCTTTTACTCGCCTCTT CAGGCGTTGAGGCCGGAGCTGGCACTGGGTGAACAGTTTACCCGCCGCTGGCAGGAAATCTCGCTCAC GCAGGAGCATCAGTCGACCTAACCATTTTCTCTCTTCACTTAGCAGGTATTTCATCGATTCTTGGGGC AATTAACTTTATCACTACCATTATCAACATGAAACCCCCTGCTATCACTCAGTATCAAACTCCCTTAT TCGTTTGAGCCGTCCTTATTACCGCTGTCCTCCTCCTCCTCTCCCTTCCAGTTCTTGCTGCGGGAATT ACCATGCTGCTCACGGACCGTAACCTAAACACCACCTTCTTCGATCCGGCAGGGGGTGGGGACCCA

>HC35

GGCCGGAATAGTGGGGACTGCCCTAAGTTTACTTATTCGAGCCGAACTTAGCCAACCCGGAGCTCTTC TAGGCGACGACCAGATTTATAATGTTATTGTTACAGCACACGCATTTGTAATAATTTTCTTTATAGTT ATGCCAATTATGATTGGGGGATTTGGTAACTGATTAATTCCACTTATGATTGGTGCCCCTGACATAGC ATTCCCCCGGATGAATAATATGAGCTTCTGACTACTCCCTCCCTCATTCCTCCTCCTTCTTGCCTCCT CAGGAGTCGAAGCCGGAGCTGGCACTGGGTGGACAGTCTATCCGCCACTGGCAGGAAATCTTGCCCAC GCAGGAGCATCAGTCGACCTAACCATCTTTTCCCTTCACTTAGCAGGTATTTCATCAATTCTTGGAGC AATTAATTTTATTACTACCATTATTAACATGAAGCCCCCTGCTATCTCCCAGTATCAGACCCCCCTGT TCGTCTGAGCTGTCCTTATTACCGCTGTCCTCCTTCTTCTGTCCCTACCAGTTCTTGCTGCAGGGATT ACAATGCTTCTCACAGATCGCAACCTAAACACCACCTTCTTCGACCCAGCAGGGGGAGGAGACCCA

>HC36

GGCCGGA- TAGTGGGGACTGCCCTAAGTTTACTTATTCGAGCCGAACTTAGCCAACCCGGAGCTCTTCTAGGCGAC GACCAGATTTATAATGTTATTGTTACAGCACACGCATTTGTAATAATTTTCTTTATAGTTATGCCAAT TATGATTGGGGGATTTGGTAACTGATTAATTCCACTTATGATTGGTGCCCCTGACATAGCATTCCCCC GGATGAATAATATGAGCTTCTGACTACTCCCTCCCTCATTCCTCCTCCTTCTTGCCTCCTCAGGAGTC GAAGCCGGAGCTGGCACTGGGTGGACAGTCTATCCGCCACTGGCAGGAAATCTTGCCCACGCAGGAGC ATCAGTCGACCTAACCATCTTTTCCCTTCACTTAGCAGGTATTTCATCAATTCTTGGAGCAATTAATT TTATTACTACCATTATTAACATGAAGCCCCCTGCTATCTCCCAGTATCAGACTCCCCTGTTCGTCTGA GCTGTCCTTATTACCGCTGTCCTCCTTCTTCTGTCCCTACCAGTTCTTGCTGCAGGGATTACAATGCT TCTCACAGATCGCAACCTAAACACCACCTTCTTCGACCCAGCAGGGGGAGGAGACCCA

>HC37

GGCCGGA- TAGTGGGGACTGCCCTAAGTTTACTTATTCGAGCCGAACTTAGCCAGCCCGGAGCTCTTCTAGGCGAC GACCAGATTTATAATGTTATTGTTACAGCACACGCATTTGTAATAATTTTCTTTATAGTTATGCCAAT TATGATTGGGGGATTTGGTAACTGATTAATTCCACTTATGATTGGTGCCCCTGACATAGCATTCCCCC GGATGAATAATATGAGCTTCTGACTACTCCCTCCCTCATTCCTCCTCCTTCTTGCCTCCTCAGGAGTC GAAGCCGGAGCTGGCACTGGGTGGACAGTCTATCCGCCACTGGCAGGAAATCTTGCCCACGCAGGAGC ATCAGTCGACCTAACCATCTTTTCCCTTCACTTAGCAGGTATTTCATCAATTCTTGGAGCAATTAATT TTATTACTACCATTATTAACATGAAACCCCCTGCTATCTCCCAGTATCAGACCCCCCTGTTCGTCTGA GCTGTCCTTATTACCGCTGTCCTCCTTCTTCTGTCCCTACCAGTTCTTGCTGCAGGGATTACAATGCT TCTCACAGATCGCAACCTAAACACCACCTTCTTCGACCCAGCAGGGGGAGGAGACCCA

>HC38

GGCCGGAATAGTGGGGACTGCCCTAAGTTTACTTATTCGAGCCGAACTTAGCCAACCCGGAGCTCTTC TAGGCGACGACCAGATTTATAATGTTATTGTTACAGCACACGCATTTGTAATAATTTTCTTTATAGTT ATGCCAATTATGATTGGGGGATTTGGTAACTGATTAATTCCACTTATGATTGGTGCCCCTGACATAGC ATTCCCCCGGATGAATAATATGAGCTTCTGACTACTCCCTCCCTCATTCCTCCTCCTTCTTGCCTCCT CAGGAGTCGAAGCCGGAGCTGGCACTGGGTGGACAGTCTATCCGCCACTGGCAGGAAATCTTGCCCAC

GCAGGAGCATCAGTCGACCTAACCATCTTTTCCCTTCACTTAGCAGGTATTTCATCAATTCTTGGAGC AATTAATTTTATTACTACCATTATTAACATGAAACCCCCTGCCATCTCCCAGTATCAGACCCCCCTGT TCGTCTGAGCTGTCCTTATTACCGCTGTCCTCCTTCTTCTGTCCCTACCAGTTCTTGCTGCAGGGATT ACAATGCTTCTCACAGATCGCAACCTAAACACCACTTTCTTCGACCCAGCAGGGGGAGGAGACCCA

>HC39

GGCCGGAATAGTGGGGACTGCCCTAAGTTTACTTATTCGAGCCGAACTTAGCCAGCCCGGAGCTCTTC TAGGCGACGACCAGATTTATAATGTTATTGTTACAGCACACGCATTTGTAATAATTTTCTTTATAGTT ATGCCAATTATGATTGGTGGATTTGGTAACTGATTAATTCCACTTATGATTGGTGCCCCTGACATAGC ATTCCCCCGGATGAATAATATGAGCTTCTGACTACTCCCTCCCTCATTCCTCCTCCTTCTTGCCTCCT CAGGAGTCGAAGCCGGAGCTGGCACTGGGTGGACAGTCTATCCGCCACTGGCAGGAAATCTTGCCCAC GCAGGAGCATCAGTCGACCTAACCATCTTTTCCCTTCACTTAGCAGGTATTTCATCAATTCTTGGAGC AATTAATTTTATTACTACCATTATTAACATGAAACCCCCTGCTATCTCCCAGTATCAGACCCCCCTGT TCGTCTGAGCTGTCCTTATTACCGCTGTCCTCCTTCTTCTGTCCCTACCAGTTCTTGCTGCAGGGATT ACAATGCTTCTCACAGATCGCAACCTAAACACCACCTTCTTCGACCCAGCAGGGGGAGGAGACCCA

>HC40

GGCCGGAATAGTGGGGACTGCCCTAAGTTTACTTATTCGAGCCGAACTTAGCCAGCCCGGAGCTCTTC TAGGCGACGACCAGATTTATAATGTTATTGTTACAGCACACGCATTTGTAATAATTTTCTTTATAGTT ATGCCAATTATGATTGGGGGATTTGGTAACTGATTAATTCCACTTATGATTGGTGCCCCTGACATAGC ATTCCCCCGGATGAATAATATGAGCTTCTGACTACTCCCTCCCTCATTCCTCCTCCTTCTTGCCTCCT CAGGAGTCGAAGCCGGAGCTGGCACTGGGTGGACAGTCTATCCGCCACTGGCAGGAAATCTTGCCCAC GCAGGAGCATCAGTCGACCTAACCATCTTTTCCCTTCACTTAGCAGGTATTTCATCAATTCTTGGGGC AATTAATTTTATTACTACCATTATTAACATGAAACCCCCTGCTATCTCCCAGTATCAGACCCCCCTGT TCGTCTGAGCTGTCCTTATTACCGCTGTCCTCCTTCTTCTGTCCCTACCAGTTCTTGCTGCAGGGATT ACAATGCTTCTCACAGATCGCAACCTAAACACCACCTTCTTCGACCCAGCAGGGGGAGGAGACCCA

>HC41

GGCCGGAATAGTGGGGACTGCCCTAAGTTTACTTATTCGAGCCGAACTTAGCCAGCCCGGAGCTCTTC TAGGCGACGACCAGATTTATAATGTTATTGTTACAGCACACGCATTTGTAATAATTTTCTTTATAGTT ATGCCAATTATGATTGGGGGATTTGGTAACTGATTAATTCCACTTATGATTGGTGCCCCTGACATAGC ATTCCCCCGGATGAATAATATGAGCTTCTGACTACTCCCTCCCTCATTCCTCCTCCTTCTTGCCTCCT CAGGAGTCGAAGCCGGAGCTGGCACTGGGTGGACAGTCTATCCGCCACTGGCAGGAAATCTTGCCCAC GCAGGAGCATCAGTCGACCTAACCATCTTTTCCCTTCACTTATCAGGTATTTCATCAATTCTTGGAGC AATTAATTTTATTACTACCATTATTAACATGAAACCCCCTGCTATCTCCCAGTATCAGACCCCCCTGT TCGTCTGAGCTGTCCTTATTACCGCTGTCCTCCTTCTTCTGTCCCTACCAGTTCTTGCTGCAGGGATT ACAATGCTTCTCACAGATCGCAACCTAAACACCACCTTCTTCGACCCAGCAGGGGGAGGAGACCCA

>HC42

GGCCGGAATAGTGGGGACTGCCCTAAGTTTGCTTATTCGAGCCGAACTTAGCCAGCCCGGAGCTCTTC TAGGCGACGACCAGATTTATAATGTTATTGTTACAGCACACGCATTTGTAATAATTTTCTTTATAGTT ATGCCAATTATGATTGGGGGATTTGGTAACTGATTAATTCCACTTATGATTGGTGCCCCTGACATAGC ATTCCCCCGGATGAATAATATGAGCTTCTGACTACTCCCTCCCTCATTCCTCCTCCTTCTTGCCTCCT CAGGAGTCGAAGCCGGAGCTGGCACTGGGTGGACAGTCTATCCGCCACTGGCAGGAAATCTTGCCCAC GCAGGAGCATCAGTCGACCTAACCATCTTTTCCCTTCACTTAGCAGGTATTTCATCAATTCTTGGAGC AATTAATTTTATTACTACCATTATTAACATGAAACCCCCTGCTATCTCCCAGTATCAGACCCCCCTGT TCGTCTGAGCTGTCCTTATTACCGCTGTCCTCCTTCTTCTGTCCCTACCAGTTCTTGCTGCAGGGATT ACAATGCTTCTCACAGATCGCAACCTAAACACCACCTTCTTCGACCCAGCAGGGGGAGGAGACCCA

>HC43

GGCCGGAATAGTGGGGACTGCCCTAAGTTTACTTATTCGAGCCGAACTTAGCCAGCCCGGAGCTCTTC TAGGCGACGACCAGATTTATAATGTTATTGTTACAGCACACGCATTTGTAATAATTTTCTTTATAGTT ATGCCAATTATGATTGGGGGATTTGGTAACTGATTAATTCCACTTATGATTGGTGCCCCTGACATAGC ATTCCCCCGGATGAATAATATGAGCTTCTGACTACTCCCTCCCTCATTCCTCCTCCTTCTTGCCTCCT CAGGAGTCGAAGCCGGAGCTGGCACTGGGTGGACAGTCTATCCGCCACTGGCAGGAAATCTTGCCCAT GCAGGAGCATCAGTCGACCTAACCATCTTTTCCCTTCACTTAGCAGGTATTTCATCAATTCTTGGAGC AATTAATTTTATTACTACCATTATTAACATGAAACCCCCTGCTATCTCCCAGTATCAGACCCCCCTGT TCGTCTGAGCTGTCCTTATTACCGCTGTCCTCCTTCTTCTGTCCCTACCAGTTCTTGCTGCAGGGATT ACAATGCTTCTCACAGATCGCAACCTAAACACCACCTTCTTCGACCCAGCAGGGGGAGGAGACCCA

>HC44

GGCCGGAATAGTGGGGACTGCCCTAAGTTTACTTATTCGAGCCGAACTTAGCCAGCCCGGAGCTCTTC TAGGCGACGACCAGATTTATAATGTTATTGTTACAGCACACGCATTTGTAATAATTTTCTTTATAGTT ATGCCAATTATGATTGGGGGATTTGGTAACTGATTAATTCCACTTATGATTGGTGCCCCTGACATAGC ATTCCCCCGGATGAACAATATGAGCTTCTGACTACTCCCTCCCTCATTCCTCCTCCTTCTTGCCTCCT CAGGAGTCGAAGCCGGAGCTGGCACTGGGTGGACAGTCTATCCGCCACTGGCAGGAAATCTTGCCCAC GCAGGAGCATCAGTCGACCTAACCATCTTTTCCCTTCACTTAGCAGGTATTTCATCAATTCTTGGAGC AATTAATTTTATTACTACCATTATTAACATGAAACCCCCTGCTATCTCCCAGTATCAGACCCCCCTGT TCGTCTGAGCTGTCCTTATTACCGCTGTCCTCCTTCTTCTGTCCCTACCAGTTCTTGCTGCAGGGATT ACAATGCTTCTCACAGATCGCAACCTAAACACCACCTTCTTCGACCCAGCAGGGGGAGGAGACCCA

>HC45

GGCCGGGATAGTGGGGACAGCCCTAAGCCTGCTCATTCGAGCTGAACTTAGCCAGCCCGGTGCTCTCC TAGGCGACGACCAGATTTATAATGTTATTGTTACAGCACATGCATTTGTAATAATTTTCTTTATAGTG ATACCAATTATGATTGGAGGCTTTGGGAATTGACTAATTCCACTTATGATCGGCGCTCCTGATATAGC ATTCCCCCGAATAAACAATATGAGCTTCTGACTGCTTCCCCCCTCATTCCTTCTTCTACTTGCTTCCT CAGGAGTTGAAGCTGGGGCCGGCACTGGATGAACGGTCTACCCCCCACTAGCAGGAAATCTTGCCCAC GCAGGAGCATCCGTCGACCTAACCATCTTCTCCCTTCACTTGGCCGGGATTTCATCAATTCTTGGTGC AATTAATTTTATTACTACCATCATTAACATGAAACCTCCCGCTATTTCCCAATATCAAACCCCACTGT TTGTATGGGCCGTTCTTATTACGGCCGTGCTTCTTCTTCTGTCGCTGCCAGTTCTTGCTGCGGGAATT ACTATGCTGCTTACGGACCGTAACCTAAACACCACCTTCTTTGACCCAGCCGGGGGAGGGGACCCT

>HC46

GGCCGGGATAGTGGGGACAGCCCTAAGCCTGCTCATTCGAGCTGAACTTAGCCAGCCCGGTGCTCTCC TAGGCGACGACCAGATTTATAATGTTATTGTTACAGCACATGCATTTGTAATAATTTTCTTTATAGTA ATACCAATTATGATTGGAGGCTTTGGGAATTGACTAATTCCACTTATGATCGGCGCTCCTGATATAGC ATTCCCCCGAATAAACAATATGAGCTTCTGACTGCTTCCCCCCTCATTCCTTCTTCTACTTGCTTCCT CAGGAGTTGAAGCTGGGGCCGGCACTGGATGAACGGTCTACCCCCCACTAGCAGGAAATCTTGCCCAC GCAGGAGCATCCGTCGACCTAACCATCTTCTCCCTTCACTTGGCCGGGATTTCATCAATTCTTGGTGC AATTAATTTTATTACTACCATCATTAACATGAAACCTCCCGCTATTTCCCAATATCAAACCCCACTGT TTGTATGGGCCGTTCTTATTACGGCCGTGCTTCTTCTTCTGTCGCTGCCAGTTCTTGCTGCGGGAATT ACTATGCTGCTTACGGACCGTAACCTAAACACCACCTTCTTTGACCCAGCCGGGGGAGGGGACCCT

>HC47

GGCCGGAATAGTGGGGACTGCCCTAAGCCTGCTCATTCGAGCTGAGCTTAGCCAGCCCGGCGCTCTCC TAGGCGACGACCAGATTTACAATGTTATTGTTACAGCACATGCATTTGTAATAATTTTTTTTATAGTA ATACCAATTATGATTGGAGGCTTTGGGAATTGGCTAATTCCACTTATGATCGGTGCCCCCGATATGGC ATTCCCCCGGATAAACAATATGAGCTTCTGATTGCTTCCCCCCTCATTTCTCCTCCTACTTGCTTCGT CAGGGGTTGAAGCCGGGGCTGGCACCGGGTGAACAGTCTACCCACCTCTAGCAGGAAATCTTGCCCAC GCAGGAGCATCCGTCGATCTAACCATCTTCTCCCTCCACTTAGCCGGGATTTCATCAATTCTTGGTGC AATTAATTTTATTACAACTATTATCAACATAAAACCCCCCGCGATCACCCAGTATCAAACACCCCTGT TTGTGTGGGCCGTCCTTATTACGGCTGTCCTTCTTCTTCTGTCACTACCAGTCCTCGCTGCGGGAATT ACAATGCTTCTCACAGACCGTAATCTTAACACCACCTTCTTCGACCCGGCTGGGGGAGGAGATCCT

>HC48

GGCCGGAATAGTGGGGACTGCCCTAAGCCTGCTCATTCGAGCTGAGCTTAGCCAGCCCGGCGCTCTCC TAGGCGACGACCAGATTTACAATGTTATTGTTACAGCACATGCATTTGTAATAATTTTTTTTATAGTA ATACCAATTATGATTGGAGGCTTTGGGAATTGACTAATTCCACTTATGATCGGTGCCCCCGATATGGC ATTCCCCCGGATAAACAATATGAGCTTCTGATTGCTTCCCCCCTCATTTCTCCTCCTACTTGCTTCGT CAGGGGTTGAAGCCGGGGCTGGCACCGGGTGAACAGTCTACCCACCTCTAGCAGGAAATCTTGCCCAC GCAGGAGCATCCGTCGATCTAACCATCTTCTCCCTCCACTTAGCCGGGATTTCATCAATTCTTGGTGC AATTAATTTTATTACAACTATTATCAACATAAAACCCCCCGCGATCACCCAGTATCAAACACCCCTGT TTGTGTGGGCCGTCCTTATTACGGCTGTCCTTCTTCTTCTGTCACTACCAGTCCTCGCTGCGGGAATT ACAATGCTTCTCACAGACCGTAACCTTAACACCACCTTCTTCGACCCGGCTGGGGGAGGAGATCCT

>HC49

GGCCGGAATAGTGGGGACTGCCCTAAGCCTGCTCATTCGAGCTGAGCTTAGCCAGCCCGGCGCTCTCC TAGGCGACGACCAGATTTACAATGTTATTGTTACAGCACATGCATTTGTAATAATTTTTTTTATAGTA ATACCAATTATGATTGGAGGCTTTGGGAATTGGCTAATTCCACTTATGATCGGTGCCCCCGATATGGC

ATTCCCCCGGATAAACAATATGAGCTTCTGATTGCTTCCCCCCTCATTTCTCCTCCTACTTGCTTCGT CAGGGGTTGAAGCCGGGGCTGGCACCGGGTGAACAGTCTACCCACCTCTAGCAGGAAATCTTGCCCAC GCAGGAGCATCCGTCGATCTAACCATCTTCTCCCTCCACTTAGCCGGGATTTCATCAATTCTTGGTGC AATTAATTTTATTACAACTATTATTAACATAAAACCCCCCGCGATCACCCAGTATCAAACACCCCTGT TTGTATGGGCCGTCCTTATTACGGCTGTCCTTCTTCTTCTGTCACTACCAGTCCTCGCTGCGGGAATT ACAATGCTTCTCACAGACCGTAACCTTAACACCACCTTCTTCGACCCGGCTGGGGGAGGAGATCCT

>HC50

GGCCGGGATAGTCGGGACAGCCCTAAGCCTGCTCATTCGAGCTGAACTTAGCCAGCCCGGTGCTCTCC TAGGCGACGACCAGATTTATAATGTTATTGTTACAGCACATGCATTTGTAATAATTTTCTTTATAGTA ATACCAATTATGATTGGAGGCTTTGGGAATTGACTAATTCCACTTATGATCGGCGCTCCTGATATAGC ATTCCCCCGAATAAACAATATGAGCTTCTGACTGCTTCCCCCCTCATTCCTTCTTCTACTTGCTTCCT CAGGAGTTGAAGCTGGGGCCGGCACTGGATGAACGGTCTACCCCCCACTAGCAGGAAATCTTGCCCAC GCAGGAGCATCCGTCGACCTAACCATCTTCTCCCTTCACTTGGCCGGGATTTCATCAATTCTTGGTGC AATTAATTTTATTACTACCATCATTAACATGAAACCTCCCGCTATTTCCCAATATCAAACCCCACTGT TTGTATGGGCCGTTCTTATTACGGCCGTGCTTCTTCTTCTGTCGCTGCCAGTTCTTGCTGCGGGAATT ACTATGCTGCTTACGGACCGTAACCTAAACACCACCTTCTTTGACCCAGCCGGGGGAGGGGACCCT

>HC51

GGCCGGGATAGTAGGGACTGCCTTAAGCCTGCTCATTCGAGCTGAACTTAGCCAACCCGGAGCTCTTC TAGGCGACGACCAGATTTATAATGTTATTGTTACAGCACACGCATTTGTAATAATTTTCTTTATAGTA ATACCAATTATGATCGGTGGATTTGGGAACTGATTAATTCCACTCATGATCGGTGCCCCTGACATAGC ATTTCCTCGGATAAACAACATGAGCTTCTGACTGCTCCCTCCGTCATTTCTTCTTCTACTTGCCTCCT CAGGGGTTGAAGCCGGGGCCGGCACTGGATGAACAGTCTACCCACCACTGGCAGGTAATCTTGCCCAC GCGGGAGCATCAGTCGACCTAACCATCTTTTCTCTTCACTTAGCAGGTATTTCGTCAATTCTTGGTGC AATCAATTTTATTACTACCATTATTAACATAAAACCCCCTGCTATTTCCCAGTATCAGACCCCCCTGT TCGTCTGGGCCGTTCTTATTACGGCCGTTCTCCTTCTTCTGTCCCTACCAGTTCTTGCTGCGGGTATC ACAATGCTCCTCACCGACCGTAACCTAAATACTACCTTCTTCGACCCAGCTGGGGGAGGAGACCCA

>SAUR1

GGCCGGAATAGTAGGAACTGCCCTAAGCCTGCTCATTCGAGCTGAGCTTAGCCAGCCTGGCGCTCTCC TTGGAGACGACCAGATTTATAATGTAATTGTTACAGCACATGCGTTTGTAATAATTTTCTTTATAGTT ATACCAATTATGATCGGTGGCTTTGGGAACTGATTAATTCCACTTATGATCGGTGCCCCTGACATAGC ATTCCCCCGAATAAATAACATGAGCTTCTGACTTCTTCCTCCCTCGTTCCTCCTTCTATTGGCCTCTT CTGGAGTTGAAGCCGGAGCCGGCACCGGATGAACAGTTTACCCCCCGCTGGCAGGAAACCTTGCCCAC GCAGGTGCATCAGTTGATTTAACAATCTTTTCCCTTCATTTAGCTGGAATTTCATCTATTCTTGGCGC TATTAATTTTATCACTACCATTATTAACATAAAACCCCCAGCCATTTCACAATACCAAACACCACTAT TCGTTTGAGCCGTTTTAATTACTGCTGTCCTACTTCTTCTGTCCCTTCCTGTTCTTGCCGCCGGGATT ACAATGCTCCTTACAGACCGAAACCTAAACACTACCTTCTTTGACCCAGCAGGGGGAGGAGACCCA

>SAUR2

AGCCGGAATAGTAGGAACTGCCCTAAGCCTGCTCATTCGAGCTGAGCTAAGCCAGCCTGGCGCTCTCC TTGGAGACGACCAGATTTATAATGTAATTGTTACAGCACATGCGTTTGTAATAATTTTCTTTATAGTT ATACCAATTATGATCGGGGGCTTTGGGAACTGATTAATTCCACTTATGATCGGTGCCCCTGACATAGC ATTCCCCCGAATAAATAACATGAGCTTCTGACTTCTTCCTCCCTCGTTCCTCCTTCTATTGGCCTCTT CCGGAGTTGAAGCCGGAGCCGGCACCGGATGAACAGTTTACCCCCCGCTGGCAGGAAACCTTGCCCAC GCAGGTGCATCAGTTGATTTAACAATCTTTTCCCTTCATTTAGCTGGAATTTCATCTATTCTTGGCGC TATTAATTTTATCACTACCATTATTAACATAAAACCCCCAGCCATTTCACAATACCAAACACCACTAT TCGTTTGAGCCGTTTTAATTACTGCTGTCCTACTTCTTCTGTCCCTTCCTGTTCTTGCCGCCGGGATT ACAATGCTCCTTACAGACCGAAACCTAAACACTACCTTCTTTGACCCAGCAGGGGGAGGAGACCCA

>DPUN1

GGCCGGAATAGTAGGAACTGCCTTAAGCCTGCTCATTCGAGCTGAACTAAGCCAGCCTGGTGCTCTCC TGGGAGACGACCAGATTTATAATGTAATTGTTACAGCACATGCATTTGTAATAATTTTCTTTATAGTA ATACCAATCATGATTGGAGGCTTTGGAAACTGATTAATCCCACTTATAATTGGTGCCCCTGACATAGC ATTCCCCCGAATAAATAACATGAGCTTCTGACTTCTACCCCCGTCATTCCTCCTCCTCCTGGCTTCGT CCGGAGTCGAAGCTGGAGCCGGTACTGGGTGAACAGTTTATCCGCCCCTGGCAGGAAACCTTGCTCAC GCAGGTGCATCAGTTGACTTAACCATCTTTTCTCTCCATCTTGCCGGAATTTCATCTATTCTTGGTGC CATTAATTTCATCACCACAATTATTAACATGAAACCTCCAGCTATTTCGCAATATCAAACACCATTAT

TTGTATGAGCCGTCTTAATTACCGCCGTATTACTCCTCCTATCCCTCCCAGTTCTTGCTGCCGGAATT ACAATGCTCCTTACAGACCGAAACCTAAACACCACTTTCTTTGACCCAGCAGGGGGAGGAGACCCA

>DPUN2

GGGCGGAATAGTAGGAACTGCCTTAAGCCTGCTCATTCGAGCTGCACTAAGCCAGCCTGGTGCTCTCC TTCGACACGACCAGATTTATAATGTAATTGTTACAGCACATGCACTTGTAATAATTTTCTTTATAGTA ATACCAATCATGATTGGAGGCTTTGGAAACTGATTAATTCCACTTATAATTGGTGCCCCTGACATAGC ATTCCCCCGAATAAATAACATAAGCTTCTGACTTCTCCCCCCATCATTCCTCCTCCTGCTAGCTTCGT CCGGAGTTGAAGCTGGGGCCGGTACTGGGTGAACAGTTTATCCGCCCCTGGCAGGAAACCTTGCTCAC GCAGGTGCATCAGTTGACTTAACCATCTTTTCTCTCCACCTGGCCGGAATTTCATCTATTCTTGGTGC CATTAATTTCATCACCACAATTATTAACATGAAACCCCCAGCTATCTCGCAATATCAGACACCATTAT TTGTATGAGCTGTCTTAATTACCGCCGTACTACTTCTTCTATCTCTCCCAGTTCTTGCTGCCGGAATT ACAATGCTCCTTACAGATCGAAACCTAAACACTACTTTCTTTGACCCTGCAGGGGGAGGAGACCCA

**S4 Table.**

**Second part: D-loop sequences included in mtDNA analyses.**

>DT4

TTGCAATAAAACACCCCAATTATTGTCAGACCTCCCACCCACCCGGCGATACTAAATGCACATTAGTA CATTAATACAT-------- AATATTTAATATATATAAAAAACGTGCTTCATGCATATTTTTTATATATGTATGCACGTAATACATAA TATTTATGATCTAATGACATATATGTATTATCAGCATTAAAACAAAGCACACCATACAAGTGATACAA AAATTAACTGCCTAGTCCCTAAAAAATGCTCCAAGAGTTTAATTGAAATATATAATGGCTGAGATCTA GGACCTAGTTATACGATTCAATAACACATTATACCAAGTACCAGCATCTCTTCTGTCGAAAATCAATT GCAGTAAGAACCGACCAACCTGTGATTTCTTAAAGCATACTGTTAATGAGgGTCAGGGACAGAAATCG TGGGGGTCGTACAACTGAACTATTACTGG

>DT6

TTGCAATAAAACACCCCAATTATTGTCAGACCTCCTACCCGCCCGGCGATACTAAATGCACATTAGTA CATTAATACAT- AATACATAATATTTAATATATATAAAAAACGTGCTTCATGCATATTTTTTATATATGTATGCACGTAA TACATAATATTTATGATCTAATGACATGTATGTAATATCAGCATTAAAATAAAGCACACCATACAAGT GATACAAAAATTAGCTGCCTAGTCCCTAAAAAATGCTCCAAGAGTTTAATTGAAATATATAATGGCTG AGATCTAGGACCTAGTTATACGATTCAATAACACATTATACCAAGTACCAGCATCTCTCCTGTCGAGA ATCAATTGCAGTAAGAACCGACCAACCTGTGATTTCTTAAAGCATACTGTTAATGAGGGTCAgGGACA GAAATCGTGGGGGTCGTACAACTGAACTATTACTGG

>DT7

TTGCAATAAAACACCCCAATTATTGTCAGACCTCCCACCCACCCGGCGATACTAAATGCACATTAGTA CATTAATACAT-------- AATATTTAATATATATAAAAAACGTGCTTCATGCATATTTTTTATATATGTATGCACGTAATACATAA TATTTATGATCTAATGACATATATGTATTATCAGCATTAAAACAAAGCACACCATACAAGTGATACAA AAATTAACTGCCTAGTCCCTAAAAAATGCTCCAAGAGTTTAATTGAAATATATAATGGCTGAGATCTA GGACCTAGTTATACGATTCAATAACACATTATACCAAGTACCAGCATCTCTTCTGTCGAAAATCAATT GCAGTAAGAACCGACCAACCTGTGATTTCTTAAAGCATACTGTTAATGAGGGTCAGGGACAGAAATCG TGGGGGTCGTACAACTGAACTATTACTGG

>DT11

TTGCAATAAAACACCCCAATTATTGTCAGACCTCCCACCCACCCGGCGATACTAAATGCACATTAGTA CATTAATACAT-------- AATATTTAATATATATAAAAAACGTGCTTCATGCATATTTTTTATATATGTATGCACGTAATACATAA TATTTATGATCTAATGACATATATGTATTATCAGCATTAAAACAAAGCACACCATACAAGTGATACAA AAATTAACTGCCTAGTCCCTAAAAAATGCTCCAAGAGTTTAATTGAAATATATAATGGCTGACATCTA GGACCTAGTTATACGATTCAATAACACATTATACCAAGTACCAGCATCTCTTCTGTCGAAAATCAATT GCAGTAAGAACCGACCAACCTGTGATTTCTTAAAGCATACTGTTAATGAGGGTCAgGGACAGAAATCG TGGGGGTCGTACAACTGAACTATTACTGG

>DT13

TTGCAATAAAACACCCCAATTATTGTCAGACCTCCCACCCGCCCGGCGATACTAAATGCACATTAGTA CATTAATACAT-------- AATATTTAATATATATAAAAAACGTGCTTCATGCATATTTTTTATATATGTATGCACGTAATACATAA TATTTATGATCTAATGACATATATGTAATATCAGCATTAAAATAAAGCACACCATACAAGTGATACAA AAATTAACTGCCTAGTCCCTAAAAAACGCTCCAAGAGTTTAATTGAAATATATAATGGCTGAAATCTA GGACCTAGTTATACGATTCAATAACACATTATACCAAGTACCAGCATCTCTTCTGTCGAAAATCAATT GCAGTAAGAACCGACCAACCTGTGATTTCTTAAAGCATACTGTTAATGAGGGTCAGgGACAGAAATCG TGGGGGTCGTACAACTGAACTATTACTGG

>DT31

TTGCAATAAAACACCCCAATTATTGTCAGACCTCCCGCCCG---- CCGATACTAAATGCACATTAGTACATTAATACAT-------- AATATTTAATATATATAAAAAACGTGCTTCATGCATATTTTTTATATATGTATGCACGTAATACATAA TATTCATGATCTAATGACATATATGTAATATCAGCATTAAAATAAAGCACACCATACAAGTGATACAA AAATTAACTGCCTAGTCCCTAAAAAATGCTCCAAGAGTTTAATTGAAATATATAATGGCTGAAATCTA GGACCTAGTTATACGATTCAATAACACATTATACCAAGTACCAGCATCTCTTCTGTCGAAAATCAATT GCAGTAAGAACCGACCAACCTGTGATTTCTTAAAGCATACTGTTAATGAGGGTCAgGGACAGAAATCG TgGGGGTCGTACAACTGAACTATTACTGG

>DT32

TTGCAATAAAACACCCCAATTATTGTCAGACCTCCCACCCG---- GCGATACTAAATGCACATTAGTACATTAATACAT-------- AATATTTAATATATATAAAAAACGTGCTTCATGCATATTTTTTATATATGTATGCACGTAATACATAA TATTTATGATCTAATGACATATATGTAATACCAGCATTAAAATAAAGCACACCATACAAGTGATACAA AAATTAGCTGCCTAGTCCCTAAAAAATGCCCCAAGAGTTTAATTGAAATATATAATGGCTGAGATCTA GGACCTAGTTATACGATTCAATAACACATTATACCAAGTACCAGCATCTCTTCTGTCGAAAATCAATT GCAGTAAGAACCGACCAACCTGTGATTTCTTAAAGCATACTGTTAATGAGGGTCAgGGACAGAAATCG TGGGGGTCGTACAACTGAACTATTACTGG

>DT288

TTGCAATAAAACACCCCAATTATTGTCAGACCTCCCACCCACCCGGCGATACTAAATGCACATTAGTA CATTAATACAT-------- AATATTTAATATATATAAAAAACGTGCTTCATGCATATTTTTTATATATGTATGCACGTAATACATAA CATTCATGATCTAATGACATATATGTATTATCAGCATTAAAATAAAGCACACCATACAAGTGATACAA AAATTAACTGCCTAATCCCTAAAAAATGCTCCAAGAGTTTAATTGAAATATATAATGGCTGAAATCTA GGACCTAGTTATACGATTCAATAACACATTATACCAAGTACCAGCATCTCTTCTGTCGAAAATCAATT GCAGTAAGAACCGACCAACCTGTGATTTCTTAAAGCATACTGTTAATGAGGGTCAGGGACAGAAATCG TGGGGGTCGTACAACTGAACTATTACTGG

>DT294

TTGCAATAAAACACCCCAATTATTGTCAGACCTCCCACCCACCCGACGATACTAAATGCACATTAGTA CATTAATACAT-------- AATATTTAATATATATAAAAAACGTGCTTCATGCATATTTTTTATATATGTATGCACGTAATACATAA TATTCATGATCTAATGACATATATGTATTATCAGCATTAAAATAAAGCACACCATACAAGTGATACAA AAATTAACTGCCTAATCCCTAAAAAATGCCCCAAGAGTTTAATTGAAATATATAATGGCTAAGATCTA GGACCTAGTTATACGATTCAATAACACATTATACCAAGTACCAGCATCTCTTCTGTCGAAAATCAATT GCAGTAAGAACCGATCAACCTGTGATTTCTTAAAGCATACTGTTAATGAGGGTCAgGGACAGAAATCG TGGGGGTCGTACAACTGAACTATTACTGG

>DT346

TTGCAATAAAACACCCCAATTATTGTCAGACCTCCCACCCACCCGGCGATACTAAATGCACATTAGTA CATTAATACAT-------- AATATTTAATATATATAAAAAACGTGCTTCATGCATATTTTTTATATATGTATGCACGTAATACATAA CATTCATGATCTAATGACATATATGTATTATCAGCATTAAAATAAAGCACACCATACAAGTAATACAA AA- TTAACTGCCTAATCCCTAAAAAATGCTCCAAGAGTTTAATTGAAATATATAATGGCTGAAATCTAGGA CCTAGTTATACGATTCAATAACACATTATACCAAGTACCAGCATCTCTTCTGTCGAAAATCAATTGCA GTAAGAACCGACCAACCTGTGATTTCTTAAAGCATACTGTTAATGAGGGTCAgGGACAGAAATCGTGG GGGTCGTACAACTGAACTATTACTGG

>DT1

TTgCAaTaAAACACCCCAATTATTGTCAGACCTCCCACCCGCCCGACGATACTAAATGCACATTAGTA CATTAATACAT-------- AATATTTAATATATATAAAAAACGTGCTTCATGCATATTTTTTATATATGTATGCACGTAATACATAA TATTTATGATCTAATGACATATATGTAATATCAGCATTGAAATAAAGCACACCATACAAGTGATACAA AAATTAACTGCCTAGTCCTTAAAAAATGTTCCAAGAGTTTAATTGAAATATATAATGGCTGAGATCTA GGACCTAGTTATACGATTCAATAACACATTATACCAAGTACCAGCATCTCTTCTGTCGAAAATCAATT GCAGTAAGAACCGACCAACCTGTGATTTCTTAAAGCATACTGTTAATGAGGGTCAGGGACAGAAATCG TGGGGGTCGTACAACTGAACTATTACTGG

>DT33

TTGCAATAAAACACCCCAATTATTGTCAGACCTCCCACCCACCCGGCGATACTAAATGCACATTAGTA CATTAATACAT-------- AATATTTAATATATATAAAAAACGTGCTTCATGCATATTTTTTATATATGTATGCACGTAATACATAA CATTCATGATCTAATGACATATATGTATTATCAGCATTAAAATAAAGCACACCATACAAGTGATACAA AAATTAACTGCCTAATCCCTAAAAAATGCTCCAAGAGTTTAATTGAAATATATAATGGCTAAGATCTA GGACCTAGTTATACAATTCAATAACACATTATACCAAGTACCAGCATCTCTTCTGTCGAAAATCAATT GCAGTAAGAACCGACCAACCTGTGATTTCTTAAAGCATACTGTTAATGAGGGTCAgGGACAGAAATCG

TGGGGGTCGTACAACTGAACTATTACTGG

>DT35

TTGCAATAAAACACCCCAATTATTGTCAGACCTCCCACCCG---- GCGATACTAAATGCACATTAGTACATTAATACAT-------- AATATTTAATATATATAAAAAACGTGCTTCATGCATATTTTTTATATATGTATGCACGTAATACATAA TATTTATGATCTAATGACATATATGTAATACCAGCATTAAAATAAAGCACACCACACAAGTGATACAA AAATTAGCTGCCTAGTCCCTAAAAAATGCCCCAAGAGTTTAATTGAAATATATAATGGCTGAGATCTA GGACCTAGTTATACGATTCAATAACACATTATACCAAGTACCAGCATCTCTTCTGTCGAAAATCAATT GCAGTAAGAACCGACCAACCTGTGATTTCTTAAAGCATACTGTTAATGAGGGTCAGGGACAAAAATCG TGGggGTCGTACAACTGAACTATTACTGG

>DT36

TTGCAATAAAACACCCCAATTATTGTCAGACCTCCCACCCGCCCGGCAATACTAAATGCACATTAGTA CATTAATACAT-------- AATATTTAATATATATAAAAAACGTGCTTCATGCATATTTTTTATATATGTATGCACGTAATACATAA TATTCATGATCTAATGACATATATGTAATATCAGCATTAAAATAAAGCACACCATACAAGTGATACAA AAATTAACTGCCTAATCCCTAAAAAATGCTCCAAGAGTTTAATTGAAATATATAATGGCTGAAATCTA GGACCTAGTTATACGATTCAATAACACATTATACCAAGTACCAGCATCTCTTCTGTCGAAAATCAATT GCAGTAAGAACCGACCAACCTGTGATTTCTTAAAGCATACTGTTAATGAGGGTCAgGGACAGAAATCG TGGGGGTCGTACAACTGAACTATTACTGG

>DT38

TTGCAATAAAACACCCCAATTATTGTCAGACCTCCCACCCACCCGACGATACTAAATGTACATTAGCA CATTAATACAT-------- AATATTTAATATATATAAAAAACGTGCTTCATGCATATTTTTTATATATGTATGCACGTAATACATAA TATTCATGATCTAATGACATATATGTAATATCAGCATTAAAATAAAGCACACCATACAAGTAATACAA AAATTAACTGTCTAATCCCTAAAAAATGTTCCAAGAGCTTAATTGAAATATATAATGGCTGAAATCTA GGACCTAGTTATACGATCCAATAACACATTATACCAAGTACCAGCATCTCTTCTGTCGAAAATCAATT GCAGTAAGAACCGACCAACCTGTGATTTCTTAAAGCATACTGTTAATGAGGATCAgGGACAGAAATCG TGGGGGTCGTACAACTGAACTATTACTGG

>DT145

TTGCAATAAAACACCCCAATTATTGTCAGACCTCCCACCCACCCGGCGATACTAAATGCACATTAGTA CATTAATACAT-------- AATATTTAATATATATAAAAAACGTGCTTCATGCATATTTTTTATATATGTATGCACGTAATACATAA TATTTATGATCTAATGACATATATGTAATATCAGCATTAAAATAAAGCACACCATACAAGTGATACAA AAATTAACTGCTTAGTCCCTAAAAAATGCTCCAAGAGTTTAATTGAAATATATAATGGCTGAGATCTA GGACCTAGTTATACGATTCAATAACACATTATACCAAGTACCAGCATCTCTTCTGTCGAAAATCAATT GCAGTAAGAACCGACCAACCTGTGATTTCTTAAAGCATACTGTTAATGAGGGTCAgGGACAGAAATCG TGGGGGTCGTACAACTGAACTATTACTGG

>DT147

TTGCAATAAAACACCCCAATTATTGTCAGACCTCCCACCCGCCCGGCGATACTAAATGCACATTAGTA CATTAATACAT-------- AATATTTAATATATATAAAAAACGTGCTTCATGCATATTTTTTATATATGTATGCACGTAATACATAA TATTTATGATCTAATGACATATATGTAATATCAGCATTAAAATAAAGCACACCATACAAGTGATACAA AAATTAACTGCCTAGTCCCTAAAAAATGCTCCAAGAGTTTAATTGAAATATATAATGGCTGAAATCTA GGACCTAGTTATACGATTCAATAACACATTATACCAAGTACCAGCATCTCTTCTGTCGAAAATCAATT GCAGTAAGAACCGACCAACCTGTGATTTCTTAAAGCATACTGTTAATGAGGGTCAgGGACAGAAATCG TGgGGGTCGTACAACTGAACTATTACTGG

>DT149

TTGCAATAAAACACCCCAATTATTGTCAGACCTCCTACCCGCCCGGCGATACTAAATGCACATTAGTA CATTAATACAT-------- AATATTTAATATATATAAAAAACGTGCTTCATGCATATTTTTTATATATGTATGCACGTAATACATAA TATTTATGATCTAATGACATGTATGTAATATCAGCATTAAAATAAAGCACACCATACAAGTGATACAA AAATTAGCTGCCTAGTCCCTAAAAAATGCTCCAAGAGTTTAATTGAAATATATAATGGCTGAGATCTA GGACCTAGTTATACGATTCAATAACACATTATACCAAGTACCAGCATCTCTCCTGTCGAGAATCAATT GCAGTAAGAACCGACCAACCTGTGATTTCTTAAAGCATACTGTTAATGAGGGTCAgGGACAGAAATCG

TGGGGGTCGTACAACTGAACTATTACTGG

>DT150

TTGCAATAAAACACCCCAATTATTGTCAGACCTCCCACCCACCCGACGATACTAAATGTACATTAGCA CATTAATACAT-------- AATATTTAATATATATAAAAAACGTGCTTCATGCATATTTTTTATATATGTATGCACGTAATACATAA TATTCATGATCTAATGACATATATGTAATATCAGCATTAAAATAAAGCACACCATACAAGTAATACAA AAATTAGCTGTCTAGTCCCTAAAAAATGTTCCAAGAGCTTAATTGAAATATATAATGGCTGAAATCTA GGACCTAGTTATACGATCCAATAACACATTATACCAAGTACCAGCATCTCTTCTGTCGAAAATCAATT GCAGTAAGAACcGACCAACCTGTGATTTCTTAAAGCATACTGTTAATGAGGATCAgGGACAGAAATCG TGGGGGTCGTACAACTGAACTATTACTGG

>DT153

TTGCAATAAAACACCCCAATTATTGTCAGACCTCCCACCCACCCGACGATACTAAATGTACATTAGCA CATTAATACAT-------- AATATTTAATATATATAAAAAACGTGCTTCATGCATATTTTTTATATATGTATGCACGTAATACATAA TATTCATGATCTAATGACATATATGTAATATCAGCATTAAAATAAAGCACACCATACAAGTAATACAA AAATTAGCTGTCTAGTCCCTAAAAAATGTTCCAAGAGCTTAATTGAAATATATAATGGCTGAAATCTA GGACCTAGTTATACGATCCAATAACACATTATACCAAGTACCAGCATCTCTTCTGTCGAAAATCAATT GCAGTAAGAACCGACCAACCTGTGATTTCTTAAAGCATACTGTTAATGAGGATCAgGGACAGAAATCG TGGGGgTCGTACAaCTGAaCTAtTACTGg

>DT117

TTGCAATAAAACACCCCAATTATTGTCAGACCTCCTACCCGCCCGGCGATACTAAATGCACATTAGTA CATTAATACAT-------- AATATTTAATATATATAAAAAACGTGCTTCATGCATATTTTTTATATATGTATGCACGTAATACATAA TATTTATGATCTAATGACATGTATGTAATATCAGCATTAAAATAAAGCACACCATACAAGTGATACAA AAATTAGCTGCCTAGTCCCTAAAAAATGCTCCAAGAGTTTAATTGAAATATATAATGGCTGAGATCTA GGACCTAGTTATACGATTCAATAACACATTATACCAAGTACCAGCATCTCTCCTGTCGAGAATCAATT GCAGTAAGAACCGACCAACCTGTGATTTCTTAAAGCATACTGTTAATGAGGGTCAgGGACAGAAATCG TGGGGGTCGTACAACTGAACTATTACTGG

>DT120

TTGCAATAAAACACCCCAATTATTGTCAGACCTCCCACCCACCCGGCGATACTAAATGCACATTAGTA CATTAATACAT-------- AATATTTAATATATATAAAAAACGTGCTTCATGCATATTTTTTATATATGTATGCACGTAATACATAA TATTTATGATCTAATGACATATATGTATTATCAGCATTAAAACAAAGCACACCATACAAGTGATACAA AAATTAACTGCCTAGTCCCTAAAAAATGCTCCAAGAGTTTAATTGAAATATATAATGGCTGAGATCTA GGACCTAGTTATACGATTCAATAACACATTATACCAAGTACCAGCATCTCTTCTGTCGAAAATCAATT GCAGTAAGAACCGACCAACCTGTGATTTCTTAAAGCATACTGTTAATGAGGGTCAGGGACAGAAATCG TGGGGGTCGTACAACTGAATTATTACTGG

>DT122

TTGCAATAAAACACCCCAATTATTGTCAGACCTCCCACCCACCCGGCGATACTAAATGCACATTAGTA CATTAATACAT-------- AATATTTAATATATATAAAAAACGTGCTTCATGCATATTTTTTATATATGTATGCACGTAATACATAA TATTTATGATCTAATGACATATATGTATTATCAGCATTAAAACAAAGCACACCATACAAGTGATACAA AAATTAACTGCCTAGTCCCTAAAAAATGCTCCAAGAGTTTAATTGAAATATATAATGGCTGAGATCTA GGACCTAGTTATACGATTCAATAACACATTATACCAAGTACCAGCATCTCTTCTGTCGAAAATCAATT GCAGTAAGAACCGACCAACCTGTGATtTCTTAAAGCATACTGTTAATGAGGGTCAGGGACAGAAATCG tGGGGGTCGTACAACTGAACTATTACTGG

>DT124

TTGCAATAAAACACCCCAATTATTGTCAGACCTCCCACCCGCCCGGCGATACTAAATGCACATTAGTA CATTAATACAT-------- AATATTTAATATATATAAAAAACGTGCTTCATGCATATTTTTTATATATGTATGCACGTAATACATAA TATTTATGATCTAATGACATATATGTAATATCAGCATTAAAATAAAGCACACCATACAAGTGATACAA AAATTAACTGCCCAATCCCTAAAAAATGCtcCAAGAGTTTAATTGAAATATATAATGGCTGAGATCTA GGACCTAGTTATACGATTCAATAACACATTATACCAAGTACCAGCATCTCTCCTGTCGAAAATCAATT GCAGTAAGAACCGACCAACCTGTGATTTCTTAAAGCATACTGTTAATGAGGGTCAGGGACAGAAATCG

TGGGGGTCGTACAACTGAACTATTACTGG

>DT125

TTGCAATAAAACACCCCAACTATTGTCAGACCTCCCACCCGCCCGGCGATACTAAATGCACATTAGTA CATTAATACAT-------- AATATTTAATATATATAAAAAACGTGCTTCATGCATATTTTTTATATATGTATGCACGTAATACATAA TATTTATGATCTAATGACATATATGTAATATCAGCATTAAAATAAAGCACACCATACAAGTGATACAA AAATTAACTGCCTAGTCCCTAAAAAATGCTCCAAGGGTTTAATTGAAATATATAATGGCTGAAATCTA GGACCTAGTTATACGATTCAATAACACATTATACCAAGTACCAGCATCTCTTCTGTCGAAAATCAATT GCAGTAAGAACcGACCAACCTGTGATTTCTTAAAGCATACTGTTAATGAGGGTCAGGGACAGAAATCG TGGGGGTCGTACAACTGAACTATTACTGG

>DT129

TTGCAATAAAACACCCCAATTATTGTCAGACCTCCCACCCGCCCGACGATACTAAATGCACATTAGTA CATTAATACAT-------- AATATTTAATATATATAAAAAACGTGCTTCATGCATATTTTTTATATATGTATGCACGTAATACATAA TATTTATGATCTAATGACATATATGTAATATCAGCATTGAAATAAAGCACACCATACAAGTGATACAA AAATTAACTGCCTAGTCCCTAAAAAATGTTCCAAGAGTTTAATTGAAATATATAATGGCTGAGATCTA GGACCTAGTTATACGATTCAATAACACATTATACCAAGTACCAGCATCTCTTCTGTCGAAAATCAATT GCAGTAAGAACCGACCAACCTGTGATTTCTTAAAGCATACTGTTAATGAGGGTCAgGGACAGAAATCG TGGGGGTCGTACAACTGAACTATTACTGG

>DT168

TTGCAATAAAACACCCCAATTATTGTCAGACCTCCCACCCGCCCGGCGATACTAAATGCACATTAGTA CATTAATACAT-------- AATATTTAATATATATAAAAAACGTGCTTCATGCATATTTTTTATATATGTATGCACGTAATACATAA TATTCATGATCTAATGACATATATGTAATATCAGCATTAAAATAAAGCACACCATACAAGTGATACAA AAATTAACTGCCTAGTCCCTAAAAAATGCTCCAAGAGTTTAATTGAAATATATAATGGCTGAAATCTA GGACCTAGTTATACGATTCAATAACACATTATACCAAGTACCAGCATCTCTTCTGTCGAAAATCAATT GCAGTAAGAACCGACCAACCTGTGATTTCTTAAAGCATACTGTTAATGAGGGTCAgGGACAGAAATCG TGGGGGTTGTACAACTGAATTATTACTGG

>DT169

TTGCAATAAAACACCCCAATTATTGTCAGACCTCCCACCCGCCCGGCAATACTAAATGCATATTAGTA CATTAATACAT-------- AATATTTAATATATATAAAAAACGTGCTTCATGCATATTTTTTATATATGTATGCACGTAATACATAA TATTCATGATCTAATGACATATATGTAATATCAGCATTAAAATAAAGCACACCATACAAGTGATACAA AAATTAACTGCCTAATCCCTAAAAAATGCTCCAAGAGTTTAATTGAAATATATAATGGCTGAAATCTA GGACCTAGTTATACGATTCAATAACACATTATACCAAGTACCAGCATCTCTTCTGTCGAAAATCGATT GCAGTAAGAACCGACCAACCTGTGATTTCTTAAAGCATACTGTTAATGAGGGTCAgGGACAGAAATCG TGGGGGTCGTACAACTGAACTATTACTGG

>DT171

TTGCAATAAAACACCCCAATTATTGTCAGACCTCCCACCCGCCCGGCGATACTAAATGCACATTAGTA CATTAATACAT-------- AATATTTAATATATATAAAAAACGTGCTTCATGCATATTTTTTATATATGTATGCACGTAATACATAA TATTTATGATCTAATGACATATATGTAATATCAGCATTAAAATAAAGCACACCATACAAGTGATACAA AAATTAGCTGCCTAGTCCCTAAAAAATGCTCCAAGAGTTTAATTGAAATATATAATGGCTAAGATCTA GGACCTAGTTATACGATTCAATAACACATTATACCAAGTACCAGCATCTCTCCTGTCGAAAATCAATT GCAGTAAGAACCGACCAACCTGTGATTTCTTAAAGCATACTGTTAATGAGGGTCAgGGACAGAAATCG TGGGGGTCGTACAACTGAACTATTACTGG

>DT172

TTGCAATAAAACACCCCAATTATTGTCAGACCTCCCACCCGCCCGGCAATACTAAATGCACATTAGTA CATTAATACAT-------- AATATTTAATATATATAAAAAACGTGCTTCATGCATATTTTTTATATATGTATGCACGTAATACATAA TATTCATGATCTAATGACATATATGTAATATCAGCATTAAAATAAAGCACACCATACAAGTGATACAA AAATTAACTGCCTAATCCCTAAAAAATGCTCCAAGAGTTTAATTGAAATATATAATGGCTGAAATCTA GGACCTAGTTATACGATTCAATAACACATTATACCAAGTACCAGCATCTCTTCTGTCGAAAATCAATT GCAGTAAGAACCGACCAACCTGTGATTTCTTAAAGCATACTGTTAATGAGGGTCAgGGACAGAAATCG

TGGGGGTCGTACAACTGAACTATTACTGG

>DT180

TTGCAATAAAACACCCCAATTATTGTCAGACCTCCCACCCG---- GCGATACTAAATGCACATTAGTACATTAATACAT-------- AATATTTAATATATATAAAAAACGTGCTTCATGCATATTTTTTATATATGTATGCACGTAATACATAA TATTTATGATCTGATGACATATATGTAATACCAGCATTAAAATAAAGCACACCATACAAGTGATACAA AAATTAGCTGCCTAGTCCCTAAAAAATGCCCCAAGAGTTTAATTGAAATATATAATGGCTAAAATCTA GGACCTAGTTATACGATTCAATAACACATTATACCAAGTACCAGCATCTCTTCTATCGAAAATCAATT GCAGTAAGAACCGACCAACCTGTGATTTCTTAAAGCATACTGTTAATGAGGGTCAgGGACAGAAATCG TGGGGGTCGTACAACTGAACTATTACTGG

>DT431

TTGCAATAAAACACCCCAATTATTGTCAGACCTCCCACCCG---- GCGATACTAAATGCACATTAGTACATTAATACAT-------- AATATTTAATATATATAAAAAACGTGCTTCATGCATATTTTTTATATATGTATGCACGTAATACATAA TATTTATGATCTAATGACATATATGTAATATCAGCATTAAAATAAAGCACACCATACAAGTGATACAA AAATTAGCTGCCTAGTCCCTAAAAAATGCCCCAAGAGTTTAATTGAAATATATAATGGCTGAGATCTA GGACCTAGTTATACGATTCAATAACACATTATACCAAGTACCAGCATCTCTTCTGTCGAAAATCAATT GCAGTAAGAACCGACCAACCTGTGATTTCTTAAAGCATACTGTTAATGAGGGTCAGGGACAGAAATCG TGGGGGTCGTACAACTGAACTATTACTGG

>DT433

TTGCAATAAAACACCCCAATTATTGTCAGACCTCCCACCCACCCGGCGATACTAAATGCACATTAGTA CATTAATACAT-------- AATATTTAATATATATAAAAAACGTGCTTCATGCATATTTTTTATATATGTATGCACGTAATACATAA CATTCATGATCTAATGACATATATGTATTATCAGCATTAAAATAAAGCACACCATACAAGTGATACAA AAATTAACTGCCTAATCCCTAAAAAATGCCCCAAGAGTTTAATTGAAATATATAATGGCTAAAATCTA GGACCTAGTTATACGATTCAATAACACATTATACCAAGTACCAGCATCTCTTCTGTCGAAAATCAATT GCAGTAAGAACCGACCAACCTGTGATTTCTTAAAGCATACTGTTAATGAGGGTCAgGGACAGAAATCG TGgGGGTCGTACAACTGAACTATTACTGG

>DT437

TTGCAATAAAACACCCCAATTATTGTCAGACCTCCCACCCG---- GCGATACTAAATGCACATTAGTACATTAATACAT-------- AATATTTAATATATATAAAAAACGTGCTTCATGCATATTTTTTATATATGTATGCACGTAATACATAA TATTTATGATCTAATGACATATATGTAATATCAGCATTAAAATAAAGCACACCATACAAGTGATACAA AAATTAACTGCCTAGTCCCTAAAAAATGCCCCAAGAGTTTAATTGAAATATATAATGGCTGAGATCTA GGACCTAGTTATACGATTCAATAACACATTATACCAAGTACCAGCATCTCTTCTGTCGAAAATCAATT GCAGTAAGAACCGACCAACCTGTGATTTCTTAAAGCATACTGTTAATGAGGGTCAGGGACAGAAATCG TGGGGGTCGTACAACTGAACTATTACTGG

>DT439

TTGCAATAAAACACCCCAATTATTGTCAGACATCCCACCCACCCGGCGATACTAAATGCACATTAGTA CATTAATACAT-------- AATATTTAATATATATAAAAAACGTGCTTCATGCATATTTTTTATATATGTATGCACGTAATACATAA TATTTATGATCTAATGACATATATGTAATATCAGCATTAAAACAAAGCACACCATACAAGTAATACAA AAACTAACTGCTTAGTCCCTAAAAAATGCTCCAAGAGTTTAATTGAAATATATAATGGCTGAGATCTA GGACCTAGTTATACGATTCAATAACACATTATACCAAGTACCAGCATCTCTTCTGTCGAAAATCAATT GCAGTAAGAACCGATCAACCTGTGATTTCTTAAAGCATACTGTTAATGAGGGTCAgGGACAGAAATCG TGGGGGTCGTACAACTGAACTATTACTGG

>DT445

TTGCAGTaAAACACCCCAATTATTGTCAGACCTCCCACCCGCCCGGCGATACTAAATGCACATTAGTA CATTAATACAT-------- AATATTTAATATATATAAAAAACGTGCTTCATGCATATTTTTTATATATGTATGCACGTAATACATAA TATTTATGATCTAATGACATATATGTAATATCAGCATTAAAATAAAGCACACCATACAAGTGATACAA AAATTAACTGCCTAGTCCCTAAAAAATGCTCCAAGAGTTTAATTGAAATATATAATGGCTGAAATCTA GGACCTAGTTATACGATTCAATAACACATTATACCAAGTACCAGCATCTCTTCTCTCGAAAATCAATT GCAGTAAGAACCGACCAACCTGTGATTTCTTAAAGCATACTGTTAATGAGGGTCAGGGACAGAAATCG

TGGGGGTCGTACAACTGAACTATTACTGG

>DT392

TTGCAATAAAACACCCCAATTATTGTCAGACCTCCCACCCGCCCGGCGATACTAAATGCACATTAGTA CATTAATACAT-------- AATATTTAATATATATAAAAAACGTGCTTCATGCATATTTTTTATATATGTATGCACGTAATACATAA TATTTATGATCTAATGACATATATGTAATATCAGCATTAAAATAAAGCACACCATACAAGTGATACAA AAATTAGCTGCCTAGTCCCTAAAAAATGCTCCAAGAGTTTAATTGAAATATATAATGGCTAAAATCTA GGACCTAGTTATACGATTCAATAACACATTATACCAAGTACCAGCATCTCTCCTGTCGAAAATCAATT GCAGTAAGAACCGACCAACCTGTGATTTCTTAAAGCATACTGTTAATGAGGGTCAgGGACAGAAATCG TGGGGGTCGTACAACTGAACTATTACTGG

>DT394

TTGCAATAAAACACCCCAATTATTGTCAGACCTCCCACCCGCCCGGCGATACTAAATGCACATTAGTA CATTAATACAT-------- AATATTTAATATATATAAAAAACGTGCTTCATGCATATTTTTTATATATGTATGCACGTAATACATAA TATTTATGATCTAATGACATATATGTAATATCAGCATTAAAATAAAGCACACCATACAAGTGATACAA AAACTAACTGCCTAGTCCCTAAAAAACGCTCCAAGAGTTTAATTGAAATATATAATGGCTCAGATCTA GGACCTAGTTATACGATTCAATAACACATTCTACCAAGTACCAGCATCTCTCCTGTCGAAAATCAATT GCAGTAAGAACCGACCAACCTGTGATTTCTTAAAGCATACTGTTAATGAGGGTCAgGGACAGAAATCG TGGGGGTCGCACAACTGAACTATTACTGG

>DT395

TTGCAATAAAACACCCCAATTATTGTCAGACCTCCCACCCGCCCGGCAATACTAAATGCACATTAGTA CATTAATACAT-------- AATATTTAATATATATAAAAAACGTGCTTCATGCATATTTTTTATATATGTATGCACGTAATACATAA TATTCATGATCTAATGACATATATGTAATATCAGCATTAAAATAAAGCACACCATACAAGTGATACAA AAATTAACTGCCTAATCCCTAAAAAATGCTCCAAGAGTTTAATTGAAATATATAATGGCTGAAATCTA GGACCTAGTTATACGATTCAATAACACATTATACCAAGTACCAGCATCTCTTCTGTCGAAAATCAATT GCAGTAAGAACCGACCAACCTGTGATtTCTTAAAGCATACTGtTAATGAGGGTCAGGGACAGAAATCG TGGGGGTCGTACAACTGAACTATTACTGG

>DT397

TTGCAATAAAACACCCCAATTATTGTCAGACCTCCCACCCGCCCGGCGATACTAAATGCACATTAGTA CATTAATACAT-------- AATATTTAATATATATAAAAAACGTGCTTCATGCATATTTTTTATATATGTATGCACGTAATACATAA TATCTATGATCTAATGACATATATGTAATATCAGCATTAAGATAAAGCACACCATACAAGTGATACAA AAATTAACTGCCTAGTCCcTAAAAAATGCTCCAAGAGTTTAATTGAAATATATAATGGCTGAGATCTA GGACCTAGTTATACGATTCAGTAACACATTATACCAAGTACCAGCATCTCtTcTGTCGAAAATCAATT GCAGTAAGAACCGATCAACCTGTGATTTCTTAAAGCATACTGTTAATGAGgGTCAgGGACAGAAATCG TGGGGGTCGTACAACTGAACTATTACTGG

>DT398

TTGCAATAAAACACCCCAATTATTGTCAGACCTCCCACCCGCCCGGCAATACTAAATGCACATTAGTA CATTAATACAT-------- AATATTTAATATATATAAAAAACGTGCTTCATGCATATTTTTTATATATGTATGCACGTAATACATAA TATTCATGATCTAATGACATATATGTAATATCAGCATTAAAATAAAGCACACCATACAAGTGATACAA AAATTAACTGCCTAATCCCTAAAAAATGCTCCAAGAGTTTAATTGAAATATATAATGGCTGAAATCTA GGACCTAGTTATACGATTCAATAACACATTATACCAAGTACCAGCATCTCTTCTGTCGAAAATCAATT GCAGTAAGAACCGACCAACCTGTGATTTCTTAAAGCATACTGTTAATGAGGGTCAGGGACAGAAATCG TGGGGGTCGTACAACTGAACTATTACTGG

>DT69

TTGCaATAAAACACCCCAATTATTGTCAGACCTCCCACCCACCCGGCGATACTAAATGCACATTAGTA CATTAATACAT-------- AATATTTAATATATATAAAAAACGTGCTTCATGCATATTTTTTATATATGTATGCACGTAATACATAA CATTCATGATCTAATGACATATATGTATTATCAGCATTAAAATAAAGCACACCATACAAGTGATACAA AAATTAACTGCCTAATCCCTAAAAAATGCTCCAAGAGTTTAATTGAAATATATAATGGCTAAGATCTA GGACCTAGTTATACGATTCAATAACACATTATACCAAGTACCAGCATCTCTTCTGTCGAAAATCAATT GCAGTAAGAACCGACCAACCTGTGATTTCTTAAAGCATACTGTTAATGAGGGTCAGGGACAGAAATCG

TGGGGGTCGTACAACTGAATTATTACTGG

>DT70

TTGCAaTAAAACACCCCAATTATTGTCAGACCTCCCACCCGCCCGGCGATACTAAATGCACATTAGTA CATTAATACAT-------- AATATTTAATATATATAAAAAACGTGCTTCATGCATATTTTTTATATATGTATGCACGTAATACATAA TATTTATGATCTAATGACATATATGTAATATCAGCATTAAAATAAAGCACACCATACAAGTGATACAA AAATTAGCTGCCTAGTCCCTAAAAAATGCTCCAAGAGTTTAATTGAAATATATAATGGCTAAGATCTA GGACCTAGTTATACGATTCAATAACACATTATACCAAGTACCAGCATCTCTCCTGTCGAAAATCAATT GCAGTAAGAACCGACCAACCTGTGATTTCTTAAAGCATACTGTTAATGAGGGTCAGGGACAGAAATCG TGGGGGTCGTACAACTGAACTATTACTGG

>DT73

TtGCaATAAAACACCCCAATTATTGTCAGACCTCCCACCCGCCCGGCGATACTAAATGCACATTAGTA CATTAATACAT-------- AATATTTAATATATATAAAAAACGTGCTTCATGCATATTTTTTATATATGTATGCACGTAATACATAA TATTTATGATCTAATGACATATATGTAATATCAGCATTAAAATAAAGCACACCATACAAGTGATACAA AAATTAACTGCCTAGTCCCTAAAAAATGCTCCAAGAGTTTAGTTGAAATATATAATGGCTGAAATCTA GGACCTAGTTATACGATTCAATAACACATTATACCAAGTACCAGCATCTCTTCTATCGAAAATCAATT GCAGTAAGAACCGACCAACCTGTGATTTCTTAAAGCATACTGTTAATGAGGGTCAGGGACAGAAATCG TGGGGGTCGTACAACTGAATTATTACTGG

>DT55

TTGCaATaAAACACCCCAATTATTGTCAGACCTCCCACCCACCCGACGATACTAAATGTACATTAGTA CATTAGTACATTAATACATAATATTTAATATATATAAAAAACGTGCTTCATGCATATTTTTTATATAT GTATGCACGTAATACATAATATTCATGATCTAATGACATATATGTAATATCAGCATTAAAATAA- GCACACCATACAAGTAATACAAAAATTAACTGCCTAGTCCCTAAAAAATGTTCCAAGAGCCTAATTGA AATATATAATGGCTGAAATCTAGGACCTAGTTATACGATCCAATAACACATTATACCAAGTACCAGCA TCTCTTCTGTCGAAAATCAATTGCAGTAAGAACCGACCAACCTGTGATTTCTTAAAGCATACTGTTAA TGAGGGTCAGGGACAGAAATCGTGGGGGTCGTACAACTGAACTATTACTGG

>DT58

TTGCAaTAaAACACCCCAATTATTGTCAGACCTCCCACCCGCCCGGCGATACTAAATGCACATTAGTA CATTAATACAT-------- AATATTTAATATATATAAAAAACGTGCTTCATGCATATTTTTTATATATGTATGCACGTAATACATAA CATTTATGATCTAATGACATATATGTAATATCAGCATTAAAATAAAGCACACCATACAAGTAATACAA AAACTAGCTGCCTAGTCCCTAAAAAATGCTACAAGAGTTTAATTGAAATATATAATGGCTAAGATCTA GGACCTAGTTATACGATTCAATAACACATTATACCAAGTACCAGCATCTCTTCTGTCGAAAATCAATT GCAGTAAGAACCGACCAACCTGTGATTTCTTAAAGCATACTGTTAATGAGGGTCAGGGACAGAAATCG TGGGGGTCGTACAACTGAACTATTACTGG

>DT59

TTGCaATAAAACACCCCAATTATTGTCAGACCTCCCACCCGCCCGGCAATACTAAATGCACATTAGTA CATTAATACAT-------- AATATTTAATATATATAAAAAACGTGCTTCATGCATATTTTTTATATATGTATGCACGTAATACATAA TATTCATGATCTAATGACATATATGTAATATCAGCATTAAAATAAAGCACACCATACAAGTGATACAA AAATTAACTGCCTAATCCCTAAAAAATGCTCCAAGAGTTTAATTGAAATATATAATGGCTGAAATCTA GGACCTAGTTATACGATTCAATAACACATTATACCAAGTACCAGCATCTCTTCTGTCGAAAATCAATT GCAGTAAGAACCGACCAACCTGTGATTTCTTAAAGCATACTGTTAATGAGGGTCAGGGACAGAAATCG TGGGGGTCGTACAACTGAACTATTACTGG

>DT193

TTGCAATAAAACACCCCAATTATTGTCAGACCTCCCACCCGCCCGGCGATACTAAATGCACATTAGTA CATTAATACAT-------- AATATTTAATATATATAAAAAACGTGCTTCATGCATATTTTTTATATATGTATGCACGTAATACATAA TATTTATGATCTAATGACATATATGTAATATCAGCATTAAAATAAAGCACACCATACAAGTGATACAA AAATTAGCTGCCTAGTCCCTAAAAAATGCTCCAAGAGTTTAATTGAAATATATGATGGCTAAAATCTA GGACCTAGTTATACGATTCAATAACACATTATACCAAGTACCAGCATCTCTCCTGTCGAAAATCAATT GCAGTAAGAACCGACCAACCTGTGATTTCTTAAAGCATACTGTTAATGAGGGTCAGGGACAGAAATCG TGGGGGTCGTACAACTGAACTATTACTGG

>DT194

TTGCAATAAAACGCCcCAATTATTGTCAGACCTCCCACCCG---- GCGATACTAAATGCACATTAGTACATTAATACAT-------- AATATTTAATATATATAAAAAACGTGCTTCATGCATATTTTTTATATATGTATGCACGTAATACATAA TATTTATGATCTAATGACATATATGTAATATCAGCATTAAAATAAAGCGCACCATACAAGTGATACAA AAATTAGCTGCCTAGTCCCTAAAAAATGCCCCAAGAGTTTAATTGAAATATATAATGGCTGAGATCTA GGACCTAGTTATACGATTCAATAACACATTATACCAAGTACCAGCATCTCTTCTGTCGAAAATCAATT GCAGTAAGAACCGACCAACCTGTGATTTCTTAAAGCATACTGTTAATGAGGGTCAGGGACAGAAATCG TGGGGGTCGTACAACTGAACTATTACTGG

>DT196

TTGCAATAAAACACCCCAATTATTGTCAGACCTCCCACCCACCCGGCGATACTAAATGCACATTAGTA CATTAATACAT-------- AATATTTAATATATATAAAAAACGTGCTTCATGCATATTTTTTATATATGTATGCACGTAATACATAA TATTTATGATCTAATGACATATATGTATTATCAGCATTAAAACAAAGCACACCATACAAGTGATACAA AAATTAACTGCCTAGTCCCTAAAAAATGCTCCAAGAGTTTAATTGAAATATATAATGGCTGAGATCTA GGACCTAGTTATACGATTCAATAACACATTATACCAAGTACCAGCATCTCTTCTGTCGAAAATCAATT GCAGTAAGAACCGACCAACCTGTGATTTCTTAAAGCATACTGTTAATGAGGGTCAgGGACAGAAATCG TGGGGGTCGTACAACTGAACTATTACTGG

>DT197

TTGCAATAAAACACCCCAATTATTGTCAGACCTCCCACCCGCCCGACGATACTAAATGCACATTAGTA CATTAATACAT-------- AATATTTAATATATATAAAAAACGTGCTTCATGCATATTTTTTATATATGTATGCACGTAATACATAA TATTTATGATCTAATGACATATATGTAATATCAGCATTGAAATAAAGCACACCATACAAGTGATACAA AAATTAACTGCCTAGTCCTTAAAAAATGTTCCAAGAGTTTAATTGAAATATATAATGGCTGAGATCTA GGACCTAGTTATACGATTCAATAACACATTATACCAAGTACCAGCATCTCTTCTGTCGAAAATCAATT GCAGTAAGAACCGACCAACCTGTGATTTCTtAAAGCATACTGTTAATGAGGGTCAGgGACAGAAATCG TGGGGGTCGTACAACTGAACTATTACTGG

>DT199

TTGCAATAAAACACCCCAATTATTGTCAGACCTCCCACCCACCCGGCGATACTAAATGCACATTAGTA CATTAATACAT-------- AATATTTAATATATATAAAAAACGTGCTTCATGCATATTTTTTATATATGTATGCACGTAATACATAA TATTTATGATCTAATGACATATATGTATTATCAGCATTAAAACAAAGCACACCATACAAGTGATACAA AAATTAACTGCCTAGTCCCTAAAAAATGCTCCAAGAGTTTAATTGAAATATATAATGGCTGAGATCTA GGACCTAGTTATACGATTCAATAACACATTATACCAAGTACCAGCATCTCTTCTGTCGAAAATCAATT GCAGTAAGAACCGACCAACCTGTGATTTCTTAAAGCATACTGTTAATGAGGGTCAGGGACAGAAATCG TGGGGGTCGTACAACTGAACTATTACTGG

>DT200 tTGCaATAAAaCACccCAatTATtGTCAGACcTcCCAcCCGCccgGCaATACTaaATGCATAtTAGTA CAtTaaTACAT-------- AaTATtTAATATATATAaAaAACGTGCtTCATGCATATtTtTTATATATGTATGCACGTAATACATAA TATTCATGATCTAATGACATATATGTAATATCAGCATTAAAATAAAGCACACCATACAAGTGATACAA AAATTAACTGCCTAATCCCTAAAAAATGCTCCAAGAGTTTAATTGAAATATATAATGGCTGAAATCTA GGACCTAGTTATACGATTCAATAACACATTATACCAAGTACCAGCATCTCTTCTGTCGAAAATCGATT GCAGTAAGAACCGACCAACCTGTGATtTCTTAAAGCATACTGtTAATGAGgGTCAGgGACAGAAATCG TGGGGGTCGTACAACTGAACTATTACTGG

>DT456

TTGCAATAAAACACCCCAATTATTGTCAGACCTCCCACCCG---- GCGATACTAAATGCACATTAGTACATTAATACAT-------- AATATTTAATATATATAAAAAACGTGCTTCATGCATATTTTTTATATATGTATGCACGTAATACATAA TATTTATGATCTAATGACATATATGTAATATCAGCATTAAAATAAAGCACACCATACAAGTGATACAA AAATTAGCTGCCTAGTCCCTAAAAAATGCCCCAAGAGTTTAATTGAAATATATAATGGCTGAGATCTA GGACCTAGTTATACGATTCAATAACACATTATACCAAGTACCAGCATCTCTTCTGTCGAAAATCAATT GCAGTAAGAACCGACCAACCTGTGATTTCTTAAAGCATACTGTTAATGAGGGTCAgGGACAGAAATCG TGGGGGTCGTACAACTGAACTATTACTGG

>DT457

TTGCAATAAAACACCCCAATTATTGCCAGACCTCCCACCCGCCCGGCGATACTAAATGCACATTAGTA CATTAATACAT-------- AATATTTAATATATATAAAAAACGTGCTTCATGCATATTTTTTATATATGTATGCACGTAATACATAA TATTCATGATCTAATGACATATATGTAATATCAGCATTAAAATAAAGCACACCATACAAGTGATACAA AAACTAACTGCCTAGTCCCTAAAAAATGCTCCAAGAGTTTAATTGAAATATATAATGGCTGAGATCTA GGACCTAGTTATACGATTCAATAACACATTATACCAAGTACCAGCATCTCTTCTGTCGAAAATCAATT GCAGTAAGAACCGACCAACCTGTGATTTCTTAAAGCATACTGTTAATGAGGGTCAgGGACAGAAATCG TGGGGGTTGTACAACTGAATTATTACTGG

>DT458

TTGCAATAAAACACCCCAATTATTGTCAGACCTCCCACCCGCCCGGCGATACTAAATGCACATTAGTA CATTAATACAT-------- AATATTTAATATATATAAAAAACGTGCTTCATGCATATTTTTTATATATGTATGCACGTAATACATAA TATTTATGATCTAATGACATATATGTAATATCAGCATTAAAATAAAGCACACCATACAAGTGATACAA AAATTAGCTGCCTAGTCCCTAAAAAATGCTCCAAGAGTTTAATTGAAATATATAATGGCTAAAATCTA GGACCTAGTTATACGATTCAATAACACATTATACCAAGTACCAGCATCTCTCCTGTCGAAAATCAATT GCAGTAAGAACCGACCAACCTGTGATtTCTTAAAGCATACTGTTAATGAGGGTCAgGGACAGAAATCG TgGGGGTCGTACAACTGAACTATTACTGG

>DT459

TTGCAATAAAACACCCCAATTATTGTCAGATCTCCCACCCGCCCGGCGATACCAAATGCACATTAGTA CATTAATACAT-------- AATATTTAATATATATAAAAAACGTGCTTCATGCATATTTTTTATATATGTATGCACGTAATACATAA TATTTATGATCTAATGACATATATGTAATATCAGCATTAAAATAAAGCACACCATACAAGTGATACAA AAATTAACTGCCTAGTCCCTAAAAAATGCTCCAAGAGTTTAATTGAAATATATAATGGCTGAAATCTA GGACCTAGTTATACGATTCAATAACACATTATACCAAGTACCAGCATCTCTTCTGTCGAAAATCAATT GCAGTAAGAACCGACCAACCTGTGATtTCTTAAAGCATACTGTTAATGAGGGTCAgGGACAAAAATCG TGGGGGTTGTACAACTGAATTATTACTGG

>DT460

TTGCAATAAAACACCCCAATTATTGTCAGACCTCCCACCCGCCCGGCGATACTAAATGTACATTAGTA CATTAATACAT-------- AATATTTAATATATATAAAAAACGTGCTTCATGCATATTTTTTATATATGTATGCACGTAATACATAA TATTTATGATCTAATGACATATATGTAATATCAGCATTAAAATAAAGCACACCATACAAGTGATACAA AAATTAACTGCCTAGTCCCTAAAAAATGCTCCAAGAGTTTAATTGAAATATATAATGGCTGAGATCTA GGACCTAGTTATACGATTCAATAACACATTATACCAAGTACCAGCATCTCTTCTGTCGAAAATCAATT GCAGTAAGAACCGACCAACCTGTGATTTCTTAAAGCATACTGTTAATGAGGGTCAGGgACAGAAATCG TGGGGGTCGTACAACTGAACTATTACTGG

>DT476

TTGCAATAAAACACCCCAATTATTGTCAGACCTCCCACCCGCCCGACGATACTAAATGCACATTAGTA CATTAATACAT-------- AATATTTAATATATATAAAAAACGTGCTTCATGCATATTTTTTATATATGTATGCACGTAATACATAA TATTTATGATCTAATGACATATATGTAATATCAGCATTGAAATAAAGCACACCATACAAGTGATACAA AAATTAACTGCCTAGTCCTTAAAAAATGTTCCAAGAGTTTAATTGAAATATATAATGGCTGAGATCTA GGACCTAGTTATACGATTCAATAACACATTATACCAAGTACCAGCATCTCTTCTGTCGAAAATCAATT GCAGTAAGAACCGACCAACCTGTGATTTCTTAAAGCATACTGTTAATGAGGGTCAgGGACAGAAATCG TGGGGGTCGTACAACTGAACTATTACTGG

>DT480

TtGCaATaaAACAcCcCaAtTatTGTCAGAcCTcCcAcCcg---- GCGaTACTaAatGCACAtTAGTACAtTaATACAT-------- aATATtTaATATATATAaAaAACGTGCtTCATGCATAtTtTtTATATATGTATGCACGTAATACATAA TATtTATGATCTAATGACATATATGTAATATCAGCAtTAAAATAAAGCACACCATACAAGTGATACAA AAATTAGCTGCCTAGTCCCTAAAAAATGCCCCAAGAGTTTAATTGAAATATATAATGGCTGAGATCTA GGACCTAGTTATACGATTCAATAACACATTATACCAAGTACCAGCATCTCTTCTGTCGAAAATCAAtT GCAGTAAGAACCGACCAACCTGTGATtTCTTAAAGCATACTGtTAATGAGGgTCAgGGACAGAAATCG TGggGGTCGTACAaCTGAaCTAtTACTGg

>DT490

TTGCAGTaAAACACCCCAATTATTGTCAGACCTCCCACCCGCCCGGCGATACTAAATGCACATTAGTA CATTAATACAT-------- AATATTTAATATATATAAAAAACGTGCTTCATGCATATTTTTTATATATGTATGCACGTAATACATAA TATTTATGATCTAATGACATATATGTAATATCAGCATTAAAATAAAGCACACCATACAAGTGATACAA AAATTAACTGCCTAGTCCCTAAAAAACGCTCCAGGAGTTTAATTGAAATATATAATGGCTGAAATCTA GGACCTAGTTATACGATTCAATAACACATTATACCAAGTACCAGCATCTCTTCTCTCGAAAATCAATT GCAGTAAGAACCGACCAACCTGTGATTTCTTAAAGCATACTGTTAATGAGGGTCAGGGACAGAAATCG TGGGGGTCGTACAACTGAACTATTACTGG

>DT491

TTGCAATAAAaCACCCCAaTTATTGTCAGACCTCCCACCCGCCCGGCGATACTAAATGCACATTAGTA CATTAATACAT-------- AATATTTAATATATATAAAAAACGTGCTTCATGCATATTTTTTATATATGTATGCACGTAATACATAA TATTTATGATCTAATGACATATATGTAATATCAGCATTGAGATAAAGCACACCATACAAGTAATACAA AAATTAACTGCCTAGTCCTTAAAAAATGCCCCAAGAGTTTAATTGAAATATATAATGGCTAAAATCTA gGACCTAGTTATACGATTCAATAACACATTATACCAAGTACCAGCATCTCTTCTGTCGAAAATCAATT GCAGTAAGAACAGACCAACCTGTGATTTCTTAAAGCATACTGTTAATGAGGGTCAGGGACAGAAATCG TGGGGGTCGTACAACTGAACTATTACTGG

>DT279

TTGCAATAAAACACCCCAATTATTGTCAGACCTCCCACCCGCCCGGCGATACTAAATGCACATTAGTA CATTAATACAT-------- AATATTTAATATATATAAAAAACGTGCTTCATGCATATTTTTTATATATGTATGCACGTAATACATAA TATTTATGATCCAATGACATATATGTAATATCAGCATTAAAATAAAGCACACCATACAAGTGATACAA AAATTAACTGCCTAGTCCCTAAAAAATGCTCCAAGAGTTTAATTGAAATATATAATGGCTGAGATCTA GGACCTAGTTATACGATTCAATAACACATTATACCAAGTACCAGCATCTCTTCTGTCGAAAATCAATT GCAGTAAGAACcGACCAACCTGTGATTTCTTAAAGCATACTGTTAATGAGGGTCAGgGACAGAAATCG TGGGGGTCGTACAACTGAACTATTACTGG

>DT280

TTGCAATAAAACACCCCAATTATTGTCAGACCTCCCACCCGCCCGGCAATACTAAATGCACATTAGTA CATTAATACAT-------- AATATTTAATATATATAAAAAACGTGCTTCATGCATATTTTTTATATATGTATGCACGTAATACATAA TATTCATGATCTAATGACATATATGTAATATCAGCATTAAAATAAAGCACACCATACAAGTGATACAA AAATTAACTGCCTAATCCCTAAAAAATGCTCCAAGAGTTTAATTGAAATATATAATGGCTGAAATCTA GGACCTAGTTATACGATTCAATAACACATTATACCAAGTACCAGCATCTCTTCTGTCGAAAATCAATT GCAGTAAGAACcGACCAACCTGTGATtTCTTAAAGCATACTGTTAATGAGGGTCAGGGACAGAAATCG TGGGGGTCGTACAACTGAACTATTACTGG

>DT283

TTGCAATAAAACACCCCAATTATTGTCAGACCCCCCACCCG---- GCGATACTAAATGCACATTAGTACATTAATACAT-------- AATATTTAATATATATAAAAAACGTGCTTTATGCATATTTTTTATATATGTATGCACGTAATACATAA TATTTATGATCTAATGACATATATGTAATACCAGCATTAAAATAAAGCACACCATACAAGTGATACAA AAATTAGCTGCCTAGTCCCTAAAAAATGCCCCAAGAGTTTAATTGAAATATATAATGGCTGAGATCTA GGACCTAGTTATACGATTCAATAACACATTATACCAAGTACCAGCATCTCTTCTGTCGAAAATCAATT GCAGTAAGAACCGACCAACCTGTGATTTCTTAAAGCATACTGTTAATGAGGGTCAgGGACAGAAATCG TGGGGGTCGTACAACTGAACTATTACTGG

>DT285

TTGCAATAAAACACCCCAATTATTGTCAGACCTCCCACCCG---- GCGATACTAAATGCACATTAGTACATTAATACAT-------- AATATTTAATATATATAAAAAACGTGCTTCATGCATATTTTTTATATATGTATGCACGTAATACATAA TATTTATGATCTAATGACATATATGTAATATCAGCATTAAAATAAAGCACACCATACAAGTGATACAA AAATTAACTGCCTAGTCCCTAAAAAATGCCCCAAGAGTTTAATTGAAATATATAATGGCTGAGATCTA GGACCTAGTTATACGATTCAATAACACATTATACCAAGTACCAGCATCTCTTCTGTCGAAAATCAATT GCAGTAAGAACCGACCAACCTGTGATTTCTTAAAGCATACTGTTAATGAGGGTCAgGGACAGAAATCG TGGGGGTCGTACAACTGAACTATTACTGG

>DT217

TTGCAATAAAACACCCCAATTATTGTCAGACCTCCCACCCG---- GCGATACTAAATGCACATTAGTACATTAATACAT-------- AATATTTAATATATATAAAAAACGTGCTTCATGCATATTTTTTATATATGTATGCACGTAATACATAA TATTTATGATCTAATGACATATATGTAATACCAGCATTGAAATAAAGCACACCATACAAGTGATACAA AAATTAGCTGCCTAGTCCCTAAAAAATGCCCCAAGAGTTTAATTGAAATATATAATGGCTGAGATCTA GGACCTAGTTATACGATTCAATAACACATTATACCAAGTACCAGCATCTCTTCTGTCGAAAATCAATT GCAGTAAGAACCGACCAACCTGTGATTTCTTAAAGCATACTGTTAATGAGGGTCAGgGACAGAAATCG TGGGGGTCGTACAACTGAACTATTACTGG

>DT225

TTGCAATAAAACACCCCAATTATTGTCAGACCTCCCACCCGCCCGGCGATACTAAATGCACATTAGTA CATTAATACAT-------- AATATTTAATATATATAAAAAACGTGCTTCATGCATATTTTTTATATATGTATGCACGTAATACATAA TATTTATGATCTAATGACATATATGTAATATCAGCATTAAAATAAAGCACACCATACAAGTGATACAA AAATTAGCTGCCTAGTCCCTAAAAAATGCTCCAAGAGTTTAATTGAAATATATAATGGCTAAGATCTA GGACCTAGTTATACGATTCAATAACACATTATACCAAGTACCAGCATCTCTCCTGTCGAAAATCAATT GCAGTAAGAACCGACCAACCTGTGATTTCTTAAAGCATACTGTTAATGAGGGTCAgGGACAGAAATCG TGGGGGTCGTACAACTGAACTATTACTGG

>DT281

TTGCAATAAAACA- CCCAATTATTGTCAGACCTCCCACCCACCCGGCGATACTAAATGCACATTAGTACATTAATACAT---

----- AATATTTAATATATATAAAAAACGTGCTTCATGCATATTTTTTATATATGTATGCACGTAATACATAA TATTTATGATCTAATGACATATATGTAATATCAGCATTAAAATAAAGCACACCATACAAGTAATACAA AAATTAGCTGCCTAGTCCCTAAAAAATGCTCCAAGAGCTTAATTGAAATATATAATGGCTGAAATCTA GGACCTAGTTATACGATTCAATAACACATTATACCAAGTACCAGCATCTCTTCTGTCGAAAATCAATT GCAGTAAGAACCGACCAACCTGTGATTTCTTAAAGCATACTATTAATGAGGGTCAGgGACAAAAATCG TGGGGGTCGTACAACTGAACTATTACTGG

>DT492

TTGCAATAAAACACCCCAATTATTGTCAGACCTCCCACCCACCCGGCGATACTAAATGCACATTAGTA CATTAATACAT-------- AATATTTAATATATATAAAAAACGTGCTTTATGCATATTTTTTATATATGTATGCACGTAATACATAA CATTTATGATCTAATGACATATATGTAATATCAGCATTAAGATAAAGCACACCATACAAGTGATACAA AAATTAACTGCCTAGTCCCTAAAAAACGCTCCAAGAGTTTAATTGAAATATATAATGGCTGAGATCTA GGACCTAGTTATACGATTCAATAACACATTATACCAAGTACCAGCATCTCTTCTGTCGAAAATCAATT GCAGTAAGAACCGACCAACCTGTGATTTCTTAAAGCATACTGTTAATGAGGGTCAgGGACAGAAATCG TgGGGGTCGTACAACTGAATTATTACTGG

>DT278

TTGCAATAAAACACCCCAATTATTGTCAGACCTCCCACCCACCCGGCGATACTAAATGCACATTAGTA CATTAATACAT-------- AATATTTAATATATATAAAAAACGTGCTTCATGCATATTTTTTATATATGTATGCACGTAATACATAA TATTTATGATCTAATGACATATATGTATTATCAGCATTGAAACAAAGCACACCATACAAGCGATACAA AAATTAACTGCCTAGTCCCTAAAAAATGCTCCAAGAGTTTAATTGAAATATATAATGGCTGAGATCTA GGACCTAGTTATACGATTCAATAACACATTATACCAAGTACCAGCATCTCTTCTGTCGAAAATCAATT GCAGTAAGAACCGACCAACCTGTGATTTCTTAAAGCATACTGTTAATGAGGGTCAgGGACAGAAAtTG TGGGGGTCGTACAACTGAACTATTACTGG

>ATL01

TTGCAATAAAACACCCCAATTATTGTCAGACCTCCCACCCACCCGACGATACTAAATGTACATTAGTA CATTAATACAT-------- AATATTTAATATATATAAAAAACGTGCTTCATGCATATTTTTTATATATGTATGCACGTAATACATAA TATTCATGATCTAATGACATATATGTAATATCAGCATTAAAATAA- GCACACCATACAAGTAATACAAAAATTAACTGCCTAGTCCCTAAAAAATGTTCCAAGAGCCTAATTGA AATATATAATGGCTGAAATCTAGGACCTAGTTATACGATCCAATAACACATTATACCAAGTACCAGCA TCTCTTCTGTCGAAAATCAATTGCAGTAAGAACCGACCAACCTGTGATTTCTTAAAGCATACTGTTAA

TGAGGGTCAGGGACAGAAATCGTGGGGGTCGTACAACTGAACTATTACTGG

>ATL02

TTGCAATAAAACACCCCAATTATTGTCAGACCTCCCACCCACCCGGCGATACTAAATGCACATTAGTA CATTAATACAT-------- AATATTTAATATATATAAAAAACGTGCTTCATGCATATTTTTTATATATGTATGCACGTAATACATAA TATTTATGATCTAATGACATATATGTATTATCAGCATTAAAACAAAGCACACCATACAAGTGATACAA AAATTAACTGCCTAGTCCCTAAAAAATGCTCCAAGAGTTTAATTGAAATATATAATGGCTGAGATCTA GGACCTAGTTATACGATTCAATAACACATTATACCAAGTACCAGCATCTCTTCTGTCGAAAATCAATT GCAGTAAGAACCGACCAACCTGTGATTTCTTAAAGCATACTGTTAATGAGGGTCAGGGACAGAAATCG TGGGGGTCGTACAACTGAACTATTACTGG

>ATL03

TTGCAGTAaAACACCCCAATTATTGTCAGACCTCCCACCCACCCGGCGATACTAAATGCACATTAGTA CATTAATACAT-------- AATATTTAATATATATAAAAAACGTGCTTCATGCATATTTTTTATATATGTATGCACGTAATACATAA CATTCATGATCTAATGACATATATGTATTATCAGCATTAAAATAAAGCACACCATACAAGTGATACAA AAATTAACTGCCTAATCCCTAAAAAATGCTCCAAGAGTTTAATTGAAATATATAATGGCTAAGATCTA GGACCTAGTTATACGATTCAATAACACATTATACCAAGTACCAGCATCTCTTCTGTCGAAAATCAATT GCAGTAAGAACCGACCAACCTGTGATTTCTTAAAGCATACTGTTAATGAGGGTCAGGGACAGAAATCG TGGGGGTCGTACAACTGAACTATTACTGG

>ATL04

TTGCAATAAAACACCCCAATTATTGTCAGACCTCCCACCCACCCGGCGATACTAAATGCACATTAGTA CATTAATACAT-------- AATATTTAATATATATAAAAAACGTGCTTCATGCATATTTTTTATATATGTATGCACGTAATACATAA CATTCATGATCTAATGACATATATGTATTATCAGCATTAAAATAAAGCACACCATACAAGTGATACAA AAATTAACTGCCTAATCCCTAAAAAATGCTCCAAGAGTTTAATTGAAATATATAATGGCTAAGATCTA GGACCTAGTTATACGATTCAATAACACATTATACCAAGTACCAGCATCTCTTCTGTCGAAAATCAATT GCAGTAAGAACCGACCAACCTGTGATTTCTTAAAGCATACTGTTAATGAGGGTCAGGGACAGAAATCG TGGGGGTCGTACAACTGAACTATTACTGG

>ATL05

TTGCAATAAAACACCCCAaTTATTGTCAGACCTCCCACCCGCCCGGCGATACTAAATGCACATTAGTA CATTAATACAT-------- AATATTTAATATATATAAAAAACGTGCTTCATGCATATTTTTTATATATGTATGCACGTAATACATAA TATCTATGATCTAATGACATATATGTAATATCAGCATTAAAATAAAGCACACCATACAAGTGATACAA AAATTAACTGCCTAGTCCCTAAAAAATGCTCCAAGAGTTTAATTGAAATATATAATGGCTGAGATCTA GGACCTAGTTATACGATTCAATAACACATTATACCAAGTACCAGCATCTCTTCTGTCGAAAATCAATT GCAGTAAGAACCGATCAACCTGTGATTTCTTAAAGCATACTGTTAATGAGGGTCAGGGACAGAAATCG TGGGGGTCGTACAACTGAACTATTACTGG

>ATL06

TTGCaATAAAACACCCCAATTATTGTCAGACCTCCCACCCGCCCGGCGATACTAAATGCACATTAGTA CATTAATACAT-------- AATATTTAATATATATAAAAAACGTGCTTCATGCATATTTTTTATATATGTATGCACGTAATACATAA TATTTATGATCTAATGACATATATGTAATATCAGCATTGAGATAAAGCACACCATACAAGTAATACAA AAATTAACTGCCTAGTCCTTAAAAAATGCTCCAAGAGTTTAATTGAAATATATAATGGCTAAAATCTA GGACCTAGTTATACGATTCAATAACACATTATACCAAGTACCAGCATCTCTTCTGTCGAAAATCAATT GCAGTAAGAACCGACCAACCTGTGATTTCTTAAAGCATACTGTTAATGAGGGTCAGGGACAGAAATCG TGGGGGTCGTACAACTGAACTATTACTGG

>ATL07

TTGCAATAAAACACCCCAATTATTGTCAGACCTCCCACCCACCCGACGATACTAAATGTACATTAGTA CATTAATACAT-------- AATATTTAATATATATAAAAAACGTGCTTCATGCATATTTTTTATATATGTATGCACGTAATACATAA TATTCATGATCTAATGACATATATGTAATATCAGCATTAAAATAA- GCACACCATACAAGTAATACAAAAATTAACTGCCTAGTCCCTAAAAAATGTTCCAAGAGCCTAATTGA AATATATAATGGCTGAAATCTAGGACCTAGTTATACGATCCAATAACACATTATACCAAGTACCAGCA TCTCTTCTGTCGAAAATCAATTGCAGTAAGAACCGACCAACCTGTGATTTCTTAAAGCATACTGTTAA

TGAGGGTCAGGGACAGAAATCGTGGGGGTCGTACAACTGAACTATTACTGG

>ATL08

TTGCAATAAAACACCCCAATTATTGTCAGACCTCCCACCCACCCGGCGATACTAAATGCACATTAGTA CATTAATACAT-------- AATATTTAATATATATAAAAAACGTGCTTCATGCATATTTTTTATATATGTATGCACGTAATACATAA TATTTATGATCTAATGACATATATGTATTATCAGCATTAAAACAAAGCACACCATACAAGTGATACAA AAATTAACTGCCTAGTCCCTAAAAAATGCTCCAAGAGTTTAATTGAAATATATAATGGCTGACATCTA GGACCTAGTTATACGATTCAATAACACATTATACCAAGTACCAGCATCTCTTCTGTCGAAAATCAATT GCAGTAAGAACCGATCAACCTGTGATTTCTTAAAGCATACTGTTAATGAGGGTCAGGGACAGAAATCG TGGGGGTCGTACAACTGAACTATTACTGG
