# Supplementary material for: Absence of spatial genetic structure in common dentex (Dentex dentex Linnaeus, 1758) in the Mediterranean Sea as evidenced by nuclear and mitochondrial molecular markers
Source: PLoS One. 2018 Sep 12;13(9):e0203866. doi: 10.1371/journal.pone.0203866 (PMC6135516; doi:10.1371/journal.pone.0203866)
Supplement: S5 Table — (DOCX) [file pone.0203866.s005.docx]

**Supportive information**

**S5 Table. *Dentex dentex* genotypes at 8 microsatellites. (0 = no amplification).**

| **Locality** | **CL168** | **CL1014** | **Ds33** | **Dxd16** | **SaGT41b** | **Sai19** | **SauE82** | **SauI41INRA** |
| --- | --- | --- | --- | --- | --- | --- | --- | --- |
| **Heraklion (North Crete, Greece)** | 173173 | 163185 | 206206 | 116124 | 137137 | 248264 | 128130 | 082094 |
|  | 173176 | 181183 | 204206 | 114128 | 133133 | 232274 | 128130 | 096098 |
|  | 173173 | 183187 | 206208 | 116116 | 135141 | 242258 | 128128 | 086088 |
|  | 173173 | 181183 | 204208 | 118120 | 133137 | 246284 | 130130 | 084090 |
|  | 173173 | 183185 | 204206 | 114120 | 133133 | 264266 | 130140 | 086104 |
|  | 0 | 187187 | 206206 | 124124 | 133137 | 0 | 128150 | 082084 |
|  | 173173 | 183187 | 204210 | 112128 | 133135 | 232290 | 130130 | 080094 |
|  | 173176 | 183187 | 204204 | 116124 | 127133 | 232236 | 130130 | 096098 |
|  | 173182 | 179183 | 204206 | 120122 | 133135 | 232232 | 128130 | 090092 |
|  | 170173 | 183189 | 204208 | 126132 | 135135 | 246258 | 118142 | 092102 |
|  | 173173 | 179183 | 204206 | 120124 | 133133 | 232236 | 130130 | 092098 |
|  | 173173 | 181183 | 208210 | 112126 | 133137 | 252262 | 132138 | 088090 |
|  | 173173 | 181197 | 208208 | 116122 | 133133 | 232236 | 130150 | 084100 |
|  | 173173 | 179181 | 206210 | 120122 | 133133 | 244266 | 130130 | 088106 |
|  | 173173 | 181183 | 204204 | 112120 | 135137 | 232278 | 128132 | 088102 |
|  | 173173 | 177181 | 208212 | 118122 | 133133 | 260262 | 128130 | 086098 |
|  | 173173 | 177183 | 204204 | 112124 | 127133 | 260282 | 128136 | 090096 |
|  | 173173 | 183187 | 198206 | 128132 | 133135 | 232264 | 142150 | 084090 |
|  | 173173 | 185193 | 204206 | 112118 | 127133 | 236252 | 128150 | 088098 |
|  | 173173 | 177181 | 204210 | 122126 | 133133 | 232258 | 128130 | 084112 |
|  | 173173 | 181181 | 204208 | 116118 | 133135 | 264268 | 130130 | 092096 |
|  | 173173 | 181181 | 204208 | 116122 | 133135 | 232262 | 128136 | 082100 |
|  | 173173 | 181187 | 198204 | 124124 | 127135 | 232252 | 132158 | 082110 |
|  | 173173 | 179183 | 206206 | 120126 | 131135 | 236262 | 128142 | 082086 |
| **North Aegean Sea (Greece)** | 173173 | 177181 | 204204 | 118120 | 133135 | 236236 | 128130 | 082102 |
|  | 164173 | 179181 | 204204 | 126128 | 135135 | 248266 | 128130 | 088094 |
|  | 173173 | 181181 | 206210 | 120134 | 133133 | 232232 | 130146 | 082082 |
|  | 173176 | 181189 | 204206 | 116116 | 135135 | 232254 | 130140 | 090108 |
|  | 173173 | 177183 | 206206 | 122122 | 133135 | 232232 | 130130 | 094110 |
|  | 167173 | 183183 | 206206 | 120124 | 133133 | 0 | 130134 | 082082 |
|  | 173173 | 181183 | 204206 | 120120 | 133133 | 252282 | 128128 | 076086 |
|  | 176176 | 181187 | 204208 | 120122 | 133137 | 232236 | 130130 | 080088 |
|  | 173173 | 163181 | 206206 | 116120 | 133133 | 0 | 128128 | 086092 |
|  | 173173 | 183183 | 204204 | 124124 | 135137 | 0 | 130142 | 090112 |
|  | 173176 | 177185 | 204206 | 126126 | 133137 | 0 | 128128 | 084084 |
|  | 176176 | 185185 | 204204 | 116120 | 133135 | 0 | 128128 | 090102 |
|  | 173173 | 177177 | 208208 | 116120 | 133133 | 0 | 128128 | 076094 |
| **Otranto (South Adriatic, Italy)** | 173173 | 181183 | 204206 | 120120 | 133137 | 234264 | 124128 | 082092 |
|  | 173173 | 177181 | 202206 | 120122 | 133135 | 266274 | 130130 | 080084 |
|  | 173173 | 181199 | 206210 | 116120 | 133137 | 270288 | 128128 | 092108 |
|  | 173173 | 183183 | 206212 | 116124 | 133135 | 232268 | 134142 | 092118 |
|  | 173173 | 181181 | 198206 | 120122 | 135137 | 236236 | 128130 | 084098 |
|  | 173173 | 177181 | 206206 | 116122 | 133133 | 252278 | 128132 | 082106 |
|  | 173173 | 185185 | 198206 | 120120 | 133133 | 258266 | 128130 | 082084 |
|  | 173173 | 181187 | 206208 | 118122 | 133135 | 258288 | 130138 | 082090 |
|  | 173173 | 181183 | 204204 | 118120 | 133133 | 232272 | 130144 | 082088 |
|  | 173176 | 185193 | 206212 | 110110 | 133135 | 252278 | 128130 | 082110 |
|  | 173173 | 177193 | 206210 | 116132 | 133133 | 240252 | 128130 | 098100 |
|  | 167173 | 181181 | 204206 | 116120 | 137137 | 232268 | 130130 | 082098 |
|  | 173173 | 181183 | 204208 | 120122 | 133133 | 232260 | 128134 | 084090 |
|  | 173173 | 181187 | 204208 | 122122 | 133135 | 268268 | 130130 | 082090 |
|  | 173173 | 179183 | 204206 | 120122 | 135135 | 232264 | 128152 | 092108 |
|  | 164173 | 183191 | 204206 | 122122 | 133133 | 258278 | 128128 | 096096 |
|  | 173173 | 181181 | 204210 | 118118 | 133133 | 232232 | 118124 | 088092 |
|  | 173173 | 181183 | 204204 | 112116 | 133133 | 266286 | 118130 | 076086 |
|  | 173173 | 183183 | 204204 | 116122 | 133133 | 232272 | 130130 | 090100 |
|  | 173173 | 181191 | 210210 | 124124 | 133133 | 264266 | 124130 | 090100 |
|  | 173173 | 181187 | 210212 | 122122 | 133133 | 232234 | 130150 | 088098 |
|  | 173173 | 181185 | 204206 | 116122 | 133133 | 270290 | 128150 | 092100 |
|  | 173173 | 187187 | 206206 | 120120 | 133135 | 264268 | 128144 | 090092 |
| **Sicily (Italy)** | 173173 | 181191 | 206208 | 116120 | 127133 | 260260 | 130152 | 084086 |
|  | 173173 | 181191 | 206208 | 116120 | 127133 | 260260 | 130152 | 084086 |
|  | 173173 | 181191 | 206208 | 116120 | 127133 | 260260 | 130152 | 084086 |
|  | 173173 | 187187 | 206206 | 118120 | 133133 | 234234 | 128134 | 084084 |
|  | 173173 | 187187 | 206210 | 118120 | 133133 | 232232 | 128134 | 084084 |
|  | 173173 | 183183 | 204204 | 122126 | 127133 | 0 | 130160 | 084104 |
|  | 173173 | 177181 | 206206 | 122126 | 133139 | 258258 | 130150 | 084090 |
|  | 173173 | 181181 | 206212 | 114116 | 133135 | 232232 | 128130 | 092108 |
|  | 173173 | 181181 | 206212 | 114116 | 133135 | 232268 | 128130 | 092108 |
|  | 173173 | 181181 | 204204 | 120122 | 133137 | 234234 | 130134 | 090092 |
|  | 173176 | 183185 | 204210 | 116120 | 133135 | 264276 | 128130 | 084084 |
|  | 173173 | 177183 | 206206 | 118120 | 133139 | 234266 | 130152 | 090098 |
|  | 173173 | 179179 | 204204 | 122122 | 133135 | 0 | 128130 | 098102 |
| **Lampedusa (Italy)** | 173173 | 177183 | 204206 | 116120 | 133139 | 232252 | 134150 | 084090 |
|  | 173173 | 187191 | 206208 | 126132 | 133135 | 232258 | 128130 | 084090 |
|  | 167173 | 183183 | 206208 | 114116 | 133135 | 232272 | 128130 | 084086 |
|  | 173173 | 181187 | 204210 | 116120 | 127133 | 232252 | 128130 | 088102 |
|  | 173173 | 191191 | 204206 | 120120 | 135135 | 256264 | 128146 | 080082 |
|  | 173173 | 179185 | 202204 | 118122 | 133133 | 254266 | 128130 | 108112 |
|  | 173173 | 181181 | 206206 | 120122 | 133135 | 234260 | 128150 | 086090 |
|  | 173173 | 179185 | 206210 | 122124 | 133135 | 232270 | 128128 | 092096 |
|  | 173179 | 183183 | 212212 | 120124 | 133133 | 266272 | 128150 | 080082 |
|  | 173173 | 177181 | 206206 | 114122 | 127135 | 232232 | 128128 | 080092 |
|  | 167173 | 179181 | 204206 | 120122 | 133133 | 232252 | 128128 | 090112 |
|  | 173173 | 187187 | 198206 | 116120 | 133133 | 236272 | 128130 | 092092 |
|  | 173173 | 181185 | 204210 | 116120 | 133135 | 232278 | 130162 | 082106 |
| **North Tunisia** | 173173 | 181181 | 0 | 116118 | 133135 | 232232 | 128130 | 090092 |
|  | 173173 | 179179 | 0 | 116120 | 137137 | 0 | 128130 | 092106 |
|  | 173173 | 183185 | 204204 | 116118 | 133133 | 232232 | 130130 | 094098 |
|  | 173173 | 181181 | 198208 | 106126 | 133133 | 236264 | 130134 | 090104 |
|  | 173173 | 177181 | 204206 | 122122 | 133133 | 232266 | 130130 | 094102 |
|  | 173173 | 183183 | 210210 | 124132 | 133139 | 0 | 128128 | 092110 |
|  | 173173 | 183185 | 204204 | 120124 | 137137 | 246252 | 134160 | 084088 |
|  | 173173 | 181181 | 206206 | 112112 | 133133 | 0 | 130130 | 098104 |
|  | 173173 | 181181 | 204204 | 120120 | 137137 | 0 | 130142 | 084088 |
|  | 173173 | 0 | 0 | 120120 | 137137 | 232232 | 128128 | 086090 |
|  | 170173 | 181183 | 204206 | 116132 | 133133 | 232232 | 130130 | 082090 |
|  | 173173 | 177177 | 0 | 116118 | 135135 | 0 | 130142 | 078078 |
|  | 173173 | 181183 | 206206 | 120126 | 133135 | 260260 | 128160 | 088092 |
|  | 173173 | 183185 | 204206 | 116122 | 133133 | 260268 | 128150 | 090096 |
| **South Tunisia** | 0 | 187187 | 206206 | 116120 | 133133 | 0 | 130130 | 080094 |
|  | 173173 | 179187 | 206206 | 120122 | 133133 | 0 | 130130 | 084084 |
|  | 173173 | 179181 | 208208 | 120120 | 133135 | 258266 | 128130 | 080092 |
|  | 173173 | 183183 | 204206 | 114126 | 133135 | 250250 | 128130 | 096098 |
|  | 179179 | 177185 | 204204 | 122124 | 133135 | 234252 | 128134 | 088088 |
|  | 173173 | 183183 | 206206 | 116120 | 135137 | 0 | 130140 | 086088 |
|  | 0 | 181181 | 204206 | 118124 | 133135 | 240260 | 128142 | 104106 |
|  | 173173 | 173183 | 206206 | 116120 | 133133 | 0 | 128152 | 084088 |
|  | 173173 | 181185 | 206206 | 128132 | 133133 | 0 | 128138 | 084084 |
|  | 173173 | 181181 | 204210 | 128128 | 133133 | 234278 | 128128 | 092102 |
|  | 164173 | 177181 | 206210 | 118128 | 133133 | 232268 | 150158 | 082098 |
|  | 173173 | 183183 | 204204 | 110122 | 133133 | 260260 | 128152 | 098102 |
|  | 173173 | 183183 | 204210 | 118118 | 133135 | 232260 | 142152 | 092104 |
|  | 173173 | 183187 | 204206 | 120132 | 133133 | 234234 | 130142 | 102114 |
| **Sardinia (Italy)** | 173176 | 177183 | 204206 | 116124 | 135139 | 234270 | 128130 | 082110 |
|  | 173173 | 181185 | 204208 | 122122 | 133135 | 258278 | 128130 | 080090 |
|  | 173173 | 177181 | 204206 | 120124 | 137139 | 266266 | 130130 | 082082 |
|  | 173176 | 181185 | 204208 | 120122 | 133133 | 232268 | 128146 | 082104 |
|  | 173173 | 181183 | 206206 | 122124 | 133133 | 264274 | 128130 | 082094 |
|  | 173176 | 181181 | 206208 | 116122 | 133135 | 232232 | 130130 | 082096 |
|  | 173176 | 183183 | 204206 | 124126 | 133137 | 232236 | 130146 | 090090 |
|  | 173176 | 181181 | 206208 | 116122 | 133135 | 232232 | 130130 | 082096 |
|  | 173173 | 181187 | 206210 | 120126 | 135135 | 232232 | 130130 | 084102 |
|  | 173173 | 181183 | 206208 | 116128 | 133133 | 272272 | 128128 | 084106 |
|  | 173173 | 181183 | 206208 | 128128 | 133133 | 232236 | 152158 | 094096 |
|  | 173173 | 181181 | 206208 | 120122 | 133137 | 232260 | 128140 | 086088 |
|  | 173173 | 177185 | 206208 | 114126 | 133135 | 232232 | 128128 | 094108 |
|  | 173173 | 183183 | 204208 | 114124 | 127133 | 232234 | 128160 | 080082 |
|  | 170173 | 181183 | 204206 | 114132 | 133137 | 232232 | 130130 | 080084 |
|  | 173176 | 179181 | 204204 | 116122 | 133137 | 232232 | 128130 | 082094 |
|  | 173173 | 181183 | 204206 | 112114 | 133133 | 264268 | 128130 | 084090 |
|  | 173173 | 181185 | 206208 | 120124 | 133137 | 232236 | 128130 | 082086 |
|  | 173173 | 181183 | 204206 | 120122 | 127133 | 232234 | 130130 | 080086 |
|  | 173173 | 0 | 204204 | 114132 | 133133 | 232232 | 130130 | 0 |
|  | 173173 | 179183 | 206208 | 120120 | 135137 | 232232 | 128130 | 084084 |
|  | 173173 | 181183 | 204206 | 112114 | 133133 | 264268 | 128130 | 084090 |
|  | 173173 | 179191 | 204204 | 120122 | 133135 | 260270 | 128152 | 086090 |
| **Giraglia (Corsica, France)** | 173173 | 181181 | 206206 | 120122 | 123133 | 232238 | 130146 | 082082 |
|  | 173173 | 179181 | 206208 | 120132 | 133135 | 232260 | 128128 | 084086 |
|  | 167179 | 179181 | 204206 | 120126 | 133137 | 232258 | 130140 | 082090 |
|  | 173173 | 185197 | 206206 | 108120 | 133133 | 232234 | 128142 | 080084 |
|  | 173173 | 181183 | 204210 | 122128 | 133137 | 232278 | 130130 | 082086 |
|  | 173173 | 181183 | 204208 | 116120 | 133133 | 232248 | 130130 | 082084 |
|  | 167173 | 185185 | 208210 | 122126 | 133135 | 232264 | 128130 | 080080 |
|  | 173173 | 177181 | 206208 | 122132 | 133135 | 264270 | 128128 | 084086 |
|  | 173173 | 183183 | 198206 | 118122 | 135137 | 234262 | 130130 | 096108 |
|  | 173179 | 177183 | 204206 | 122122 | 127133 | 232264 | 128130 | 088094 |
|  | 173173 | 185193 | 206206 | 114122 | 127137 | 232264 | 128152 | 088094 |
|  | 173173 | 181181 | 204212 | 120126 | 133137 | 272274 | 128130 | 086090 |
|  | 173173 | 181183 | 204204 | 110118 | 133133 | 232282 | 134146 | 086104 |
|  | 173173 | 179181 | 206206 | 114126 | 133133 | 232236 | 128134 | 084088 |
|  | 173173 | 173187 | 206206 | 120124 | 135139 | 234258 | 128128 | 088106 |
|  | 173173 | 181181 | 204204 | 120120 | 133135 | 254262 | 128128 | 098112 |
|  | 173173 | 177183 | 206208 | 120124 | 133137 | 232260 | 130140 | 094094 |
|  | 173173 | 181181 | 204204 | 118118 | 133133 | 234266 | 128130 | 082092 |
|  | 173176 | 177181 | 206208 | 122124 | 133135 | 234276 | 118134 | 088088 |
|  | 173173 | 181183 | 204210 | 122128 | 133137 | 232278 | 130130 | 082086 |
|  | 173173 | 179185 | 198206 | 120120 | 133133 | 234246 | 128150 | 084112 |
|  | 173173 | 181183 | 204204 | 116124 | 133133 | 232264 | 136140 | 094106 |
|  | 173173 | 181187 | 204204 | 124132 | 127137 | 232260 | 130130 | 084108 |
|  | 173176 | 181181 | 206210 | 120120 | 133137 | 234234 | 130130 | 090098 |
|  | 173173 | 181197 | 204206 | 116122 | 133135 | 232246 | 128128 | 088090 |
| **St Florent (Corsica, France)** | 173173 | 183187 | 204204 | 122122 | 133135 | 232234 | 128130 | 086108 |
|  | 173173 | 183185 | 202204 | 118122 | 135141 | 232270 | 128130 | 082092 |
|  | 173173 | 181183 | 198212 | 112124 | 133135 | 234264 | 130146 | 094100 |
|  | 173173 | 163177 | 204204 | 122124 | 133133 | 246266 | 130130 | 086092 |
|  | 173173 | 181181 | 204206 | 106116 | 133135 | 236254 | 128130 | 088100 |
|  | 173179 | 181183 | 198206 | 120124 | 133141 | 254258 | 130158 | 090090 |
|  | 173173 | 179181 | 206206 | 120120 | 133133 | 246260 | 128150 | 080088 |
|  | 173173 | 181183 | 204206 | 116128 | 133135 | 236236 | 128128 | 080106 |
|  | 173173 | 181181 | 206206 | 118120 | 133137 | 260260 | 130146 | 082116 |
|  | 173173 | 187187 | 206210 | 120120 | 135137 | 234234 | 128130 | 088090 |
|  | 173173 | 179185 | 206206 | 122126 | 133133 | 236260 | 128142 | 084114 |
|  | 173173 | 181181 | 182204 | 112122 | 135137 | 0 | 130130 | 084098 |
|  | 173173 | 179179 | 180180 | 112126 | 0 | 0 | 130130 | 084088 |
|  | 173173 | 177185 | 204206 | 126128 | 133135 | 234234 | 130130 | 092092 |
|  | 173173 | 0 | 0 | 114114 | 133133 | 234234 | 128134 | 084090 |
|  | 173173 | 0 | 206206 | 118120 | 133133 | 0 | 128142 | 078078 |
|  | 173173 | 179183 | 204204 | 120120 | 133133 | 232268 | 130134 | 088092 |
|  | 173173 | 183183 | 212212 | 114118 | 133137 | 0 | 128130 | 082090 |
| **Galeria (Corsica, France)** | 173173 | 183185 | 206210 | 122122 | 135137 | 0 | 128130 | 098100 |
|  | 173173 | 189191 | 204206 | 122128 | 135137 | 270274 | 128130 | 088088 |
|  | 173173 | 177177 | 206206 | 122122 | 133133 | 236260 | 128132 | 088098 |
|  | 173173 | 179183 | 204206 | 120120 | 133137 | 254260 | 132134 | 086110 |
|  | 173173 | 177185 | 204206 | 120120 | 133135 | 236266 | 130130 | 078102 |
|  | 173173 | 183185 | 206208 | 120120 | 133139 | 232234 | 128128 | 082086 |
|  | 173173 | 177183 | 206206 | 120120 | 133133 | 232266 | 130140 | 090102 |
|  | 173173 | 181183 | 198212 | 112124 | 133135 | 234264 | 130146 | 094100 |
|  | 173173 | 183189 | 198204 | 120124 | 133135 | 0 | 128130 | 090090 |
|  | 173173 | 181181 | 206208 | 112116 | 133133 | 232264 | 130150 | 082102 |
|  | 173173 | 181181 | 198204 | 120126 | 133141 | 260270 | 130142 | 088092 |
|  | 173173 | 181185 | 202206 | 110118 | 133133 | 232268 | 156160 | 084088 |
|  | 173173 | 183183 | 212212 | 122128 | 133137 | 230260 | 130138 | 080086 |
|  | 173173 | 177183 | 206206 | 120126 | 133135 | 234258 | 130140 | 086090 |
|  | 173173 | 181183 | 206206 | 120120 | 133133 | 258260 | 130142 | 086098 |
|  | 173173 | 177183 | 206206 | 120126 | 133135 | 258258 | 130142 | 082082 |
|  | 173173 | 173181 | 206206 | 112120 | 133139 | 232232 | 128134 | 082090 |
|  | 173173 | 173179 | 204206 | 120124 | 133139 | 232232 | 128158 | 088100 |
|  | 0 | 191191 | 206206 | 122132 | 135135 | 0 | 142150 | 084092 |
|  | 173173 | 177185 | 210212 | 118120 | 133135 | 232232 | 128128 | 092092 |
|  | 173173 | 181181 | 206208 | 116124 | 133133 | 236270 | 130142 | 090108 |
|  | 173173 | 179181 | 204204 | 116116 | 127133 | 0 | 128134 | 080110 |
|  | 173173 | 183183 | 206208 | 118120 | 135135 | 0 | 118134 | 0 |
|  | 173173 | 177183 | 206206 | 116116 | 133135 | 0 | 128128 | 096106 |
|  | 173173 | 165181 | 206206 | 122122 | 133133 | 260270 | 128150 | 086090 |
|  | 173173 | 181181 | 208208 | 114116 | 133133 | 234240 | 130130 | 086086 |
|  | 173173 | 163179 | 204206 | 112120 | 133133 | 232232 | 128130 | 090108 |
|  | 173176 | 181183 | 204210 | 112116 | 131133 | 258264 | 128130 | 098100 |
|  | 173173 | 183183 | 204206 | 116132 | 133137 | 234236 | 128142 | 090106 |
|  | 173173 | 181187 | 204212 | 122126 | 133137 | 0 | 130154 | 084098 |
|  | 173173 | 181181 | 206212 | 122122 | 133133 | 232274 | 130150 | 094114 |
| **Ajaccio (Corsica, France)** | 173176 | 181181 | 206206 | 118122 | 133137 | 232236 | 128156 | 082106 |
|  | 173173 | 183183 | 206206 | 116124 | 133135 | 0 | 128142 | 092116 |
|  | 176176 | 163179 | 204204 | 120122 | 133139 | 232232 | 128130 | 090108 |
|  | 173173 | 183183 | 206206 | 120120 | 133133 | 232232 | 128136 | 080090 |
|  | 173173 | 163185 | 206206 | 120120 | 135135 | 232270 | 130130 | 082098 |
|  | 173173 | 179183 | 204204 | 122126 | 135135 | 0 | 128130 | 090098 |
|  | 173173 | 179193 | 212212 | 118118 | 127133 | 0 | 128128 | 104106 |
|  | 0 | 181181 | 182228 | 120120 | 133135 | 0 | 130134 | 084102 |
|  | 173173 | 185191 | 204206 | 122124 | 133135 | 232232 | 130142 | 092092 |
|  | 173173 | 181187 | 206206 | 120132 | 133133 | 0 | 128128 | 082106 |
|  | 173173 | 181187 | 204204 | 120120 | 127133 | 262264 | 118130 | 098100 |
|  | 173173 | 177185 | 204208 | 120122 | 133133 | 266274 | 130130 | 082082 |
|  | 164173 | 183185 | 204206 | 114126 | 133133 | 232266 | 130140 | 088094 |
|  | 173173 | 189189 | 210210 | 114122 | 133133 | 232232 | 130152 | 084086 |
|  | 173173 | 181185 | 206212 | 112132 | 127135 | 260264 | 128130 | 088100 |
| **Bonifacio (Corsica, France)** | 173173 | 181193 | 206206 | 116120 | 133133 | 0 | 130134 | 082102 |
|  | 173173 | 177181 | 204206 | 116122 | 133135 | 266266 | 128128 | 078084 |
|  | 0 | 181183 | 204204 | 116124 | 133133 | 0 | 130130 | 088110 |
|  | 173173 | 181187 | 204212 | 116120 | 133135 | 0 | 130142 | 082082 |
|  | 173173 | 181183 | 198204 | 112128 | 127135 | 0 | 128136 | 084088 |
|  | 170173 | 177183 | 204208 | 116120 | 133135 | 260270 | 128130 | 080090 |
|  | 173173 | 181181 | 204204 | 126126 | 133137 | 234236 | 130136 | 084084 |
|  | 173173 | 179183 | 206206 | 120132 | 133133 | 232234 | 130150 | 084114 |
|  | 173176 | 181185 | 204210 | 120124 | 127133 | 236236 | 128130 | 102110 |
|  | 173173 | 181187 | 208210 | 122132 | 133133 | 232236 | 118130 | 092092 |
|  | 173173 | 177185 | 198198 | 120126 | 127135 | 232232 | 128130 | 084096 |
|  | 173173 | 181187 | 204204 | 120140 | 133133 | 0 | 130130 | 082092 |
|  | 173173 | 183183 | 206210 | 120132 | 133135 | 236236 | 128128 | 096098 |
|  | 173173 | 177181 | 204206 | 120120 | 133133 | 232272 | 128128 | 090092 |
|  | 173173 | 181181 | 204206 | 122126 | 133133 | 234262 | 128150 | 084092 |
|  | 173173 | 181183 | 204206 | 116124 | 137139 | 232254 | 130134 | 082084 |
|  | 173173 | 161181 | 204206 | 116122 | 135135 | 234278 | 130130 | 094094 |
|  | 173173 | 181181 | 204206 | 114116 | 127133 | 0 | 128128 | 104110 |
|  | 173173 | 181199 | 206206 | 120126 | 135141 | 232236 | 128128 | 082084 |
|  | 173173 | 181181 | 206206 | 108120 | 133135 | 234274 | 150152 | 084094 |
|  | 173173 | 181183 | 204208 | 120122 | 133137 | 234260 | 128130 | 084102 |
|  | 173173 | 183183 | 198206 | 112112 | 137137 | 236236 | 128130 | 086090 |
|  | 173173 | 177179 | 208208 | 128132 | 133133 | 0 | 130130 | 102106 |
|  | 173173 | 181183 | 204204 | 120124 | 133133 | 0 | 130146 | 088088 |
| **St Raphael (France)** | 173173 | 181181 | 204206 | 120122 | 133133 | 232248 | 130130 | 082098 |
|  | 173173 | 187187 | 204206 | 116126 | 127133 | 232232 | 130142 | 090094 |
|  | 173173 | 181181 | 198204 | 114124 | 133133 | 234260 | 128128 | 082098 |
|  | 173173 | 183183 | 206206 | 116124 | 133139 | 260262 | 128130 | 084090 |
|  | 167173 | 181183 | 204210 | 120120 | 127135 | 248266 | 130160 | 084090 |
|  | 173173 | 165187 | 204210 | 120124 | 133135 | 236278 | 128160 | 084098 |
|  | 173173 | 183197 | 204210 | 116120 | 133133 | 236266 | 128130 | 090098 |
|  | 173173 | 183185 | 206210 | 116118 | 133137 | 236286 | 128130 | 094098 |
|  | 173176 | 181183 | 206206 | 116122 | 133139 | 232258 | 134142 | 092098 |
|  | 173173 | 179181 | 204206 | 116124 | 133135 | 232264 | 130138 | 084102 |
|  | 173173 | 181183 | 198208 | 120122 | 133135 | 260270 | 128130 | 092094 |
|  | 173176 | 163183 | 198206 | 116118 | 133137 | 236236 | 130154 | 086088 |
|  | 173173 | 187187 | 208208 | 114116 | 133137 | 252270 | 130134 | 098102 |
|  | 173173 | 183187 | 206206 | 118122 | 133133 | 240260 | 128150 | 088094 |
| **Baleares (Spain)** | 173173 | 0 | 204204 | 120122 | 133135 | 0 | 130130 | 096098 |
|  | 173173 | 181187 | 204204 | 120120 | 133135 | 234234 | 128150 | 084102 |
|  | 173173 | 161181 | 206208 | 120122 | 135135 | 232256 | 128134 | 098104 |
|  | 173173 | 181183 | 204206 | 116120 | 133133 | 260264 | 128150 | 088090 |
|  | 173173 | 177177 | 0 | 122122 | 135135 | 0 | 128130 | 088102 |
|  | 173173 | 161181 | 206208 | 116122 | 133133 | 0 | 128128 | 098102 |
|  | 173173 | 177187 | 204206 | 114122 | 133137 | 232248 | 128130 | 094098 |
|  | 173173 | 163191 | 206206 | 120124 | 133135 | 0 | 128130 | 094102 |
|  | 173173 | 179199 | 204206 | 120126 | 135137 | 258260 | 126150 | 082090 |
|  | 173173 | 181187 | 204206 | 110132 | 133133 | 232260 | 124128 | 092098 |
|  | 173173 | 177187 | 208208 | 122132 | 133137 | 0 | 128130 | 096102 |
|  | 173173 | 181181 | 198204 | 116122 | 137137 | 0 | 130130 | 076076 |
|  | 173173 | 181183 | 206206 | 120124 | 127133 | 0 | 142150 | 084086 |
|  | 173173 | 181183 | 206206 | 112122 | 133133 | 232260 | 118130 | 082090 |
|  | 173173 | 181181 | 202204 | 122122 | 133133 | 236268 | 128130 | 090096 |
|  | 173173 | 181183 | 212212 | 112120 | 133133 | 0 | 130134 | 088088 |
|  | 173173 | 181185 | 204210 | 118124 | 133133 | 0 | 130130 | 102106 |
|  | 173173 | 179183 | 206210 | 116122 | 133133 | 262284 | 128134 | 078090 |
|  | 173179 | 177179 | 204204 | 126128 | 133135 | 232232 | 128130 | 088088 |
| **Alicante (Spain)** | 173173 | 183187 | 204210 | 118120 | 133137 | 236256 | 136142 | 088098 |
|  | 173176 | 177181 | 206212 | 114118 | 133137 | 234266 | 130160 | 086096 |
|  | 173173 | 177183 | 204206 | 118120 | 133133 | 232232 | 130156 | 086088 |
|  | 173173 | 181181 | 206206 | 112132 | 133135 | 232234 | 130150 | 090100 |
|  | 173173 | 181181 | 210210 | 110118 | 133137 | 260264 | 150150 | 080082 |
|  | 173173 | 183189 | 204206 | 118120 | 133133 | 232258 | 130136 | 088106 |
|  | 173173 | 161181 | 206210 | 126126 | 133133 | 232232 | 128142 | 084088 |
|  | 173173 | 181183 | 206206 | 120122 | 133133 | 262262 | 130142 | 080082 |
|  | 173173 | 179187 | 210210 | 0 | 127139 | 232266 | 128130 | 084102 |
|  | 173173 | 181185 | 206208 | 122122 | 133133 | 236236 | 134146 | 084086 |
|  | 173176 | 183183 | 206206 | 126132 | 133135 | 232262 | 128130 | 082082 |
|  | 173173 | 181193 | 206206 | 116122 | 127133 | 232232 | 128134 | 084090 |
|  | 173179 | 163193 | 198206 | 118120 | 133135 | 260262 | 128152 | 082094 |
|  | 173173 | 181183 | 204204 | 112112 | 133133 | 232236 | 128130 | 080098 |
|  | 173173 | 181181 | 206212 | 126132 | 133137 | 236270 | 130162 | 090090 |
|  | 173173 | 181199 | 202204 | 116120 | 133133 | 260270 | 130134 | 082084 |
|  | 167173 | 179187 | 202206 | 118122 | 133137 | 232292 | 130142 | 104106 |
|  | 173173 | 183189 | 204212 | 120120 | 133135 | 232236 | 128156 | 082092 |
|  | 173173 | 179181 | 204208 | 124124 | 133133 | 232232 | 128160 | 088088 |
|  | 173173 | 181191 | 204206 | 116120 | 133135 | 232236 | 128130 | 082086 |
|  | 173173 | 177181 | 204204 | 120122 | 133133 | 232260 | 128134 | 088090 |
| **Faro (Portugal)** | 170170 | 185187 | 228232 | 114154 | 143143 | 240266 | 128130 | 080108 |
|  | 170173 | 187187 | 224226 | 116116 | 143143 | 242278 | 142164 | 106106 |
|  | 176176 | 185185 | 222222 | 116146 | 145151 | 244258 | 116124 | 116116 |
|  | 0 | 185185 | 186204 | 114128 | 133133 | 234234 | 128134 | 106106 |
|  | 176176 | 181185 | 182186 | 126126 | 133149 | 234234 | 122134 | 102102 |
|  | 170176 | 185187 | 220252 | 120122 | 143143 | 242242 | 138144 | 080108 |
|  | 170170 | 185187 | 216242 | 114116 | 143143 | 248248 | 122150 | 094094 |
|  | 170170 | 187187 | 218240 | 0 | 143143 | 250268 | 138148 | 110110 |
|  | 170170 | 185185 | 218234 | 116116 | 143143 | 254266 | 126136 | 0 |
|  | 170170 | 185187 | 226228 | 0 | 143143 | 250292 | 136140 | 092096 |
|  | 170170 | 185185 | 228232 | 116128 | 143143 | 238272 | 128136 | 094094 |
|  | 170176 | 185189 | 206218 | 120138 | 143143 | 238268 | 144146 | 112116 |
|  | 170170 | 185187 | 228234 | 0 | 143143 | 248268 | 136136 | 104108 |
|  | 170170 | 187187 | 230240 | 0 | 143143 | 242248 | 142158 | 100100 |
|  | 170170 | 185187 | 232240 | 0 | 143143 | 238268 | 118138 | 092098 |
|  | 170170 | 187187 | 226230 | 0 | 143143 | 242270 | 126128 | 0 |
|  | 170170 | 187187 | 216234 | 0 | 143143 | 268270 | 132138 | 096104 |
|  | 170170 | 187187 | 230232 | 116116 | 143143 | 238246 | 126140 | 108108 |
|  | 170176 | 185187 | 238242 | 0 | 143143 | 244244 | 146158 | 080100 |
|  | 170170 | 185187 | 222232 | 116116 | 143143 | 248258 | 136136 | 104116 |
|  | 170170 | 187187 | 204240 | 116128 | 143143 | 238248 | 132140 | 102108 |
|  | 170170 | 185185 | 224242 | 0 | 143143 | 238244 | 132136 | 094094 |
|  | 170170 | 185187 | 226240 | 116122 | 143143 | 266268 | 134140 | 080090 |
|  | 170170 | 185187 | 220228 | 116142 | 143143 | 254266 | 138140 | 104112 |
| **Bay of Biscay (France)** | 173173 | 183191 | 204204 | 120132 | 133133 | 234240 | 128128 | 082084 |
|  | 173173 | 181181 | 204206 | 120132 | 133141 | 236260 | 130130 | 090104 |
|  | 173184 | 183185 | 204206 | 116132 | 135135 | 266280 | 130132 | 096100 |
|  | 173173 | 183193 | 204212 | 122124 | 127133 | 234260 | 128128 | 082092 |
|  | 173173 | 181183 | 204204 | 116120 | 131131 | 234260 | 128132 | 082096 |
|  | 173173 | 181183 | 204206 | 116120 | 127133 | 272278 | 128134 | 082086 |
|  | 173173 | 183187 | 204206 | 122132 | 133143 | 0 | 128142 | 088104 |
|  | 173173 | 181181 | 204206 | 116122 | 133143 | 232238 | 130142 | 082098 |
