# Supplementary figures and images for: Absence of spatial genetic structure in common dentex (Dentex dentex Linnaeus, 1758) in the Mediterranean Sea as evidenced by nuclear and mitochondrial molecular markers
Source: PLoS One. 2018 Sep 12;13(9):e0203866. doi: 10.1371/journal.pone.0203866 (PMC6135516; doi:10.1371/journal.pone.0203866)

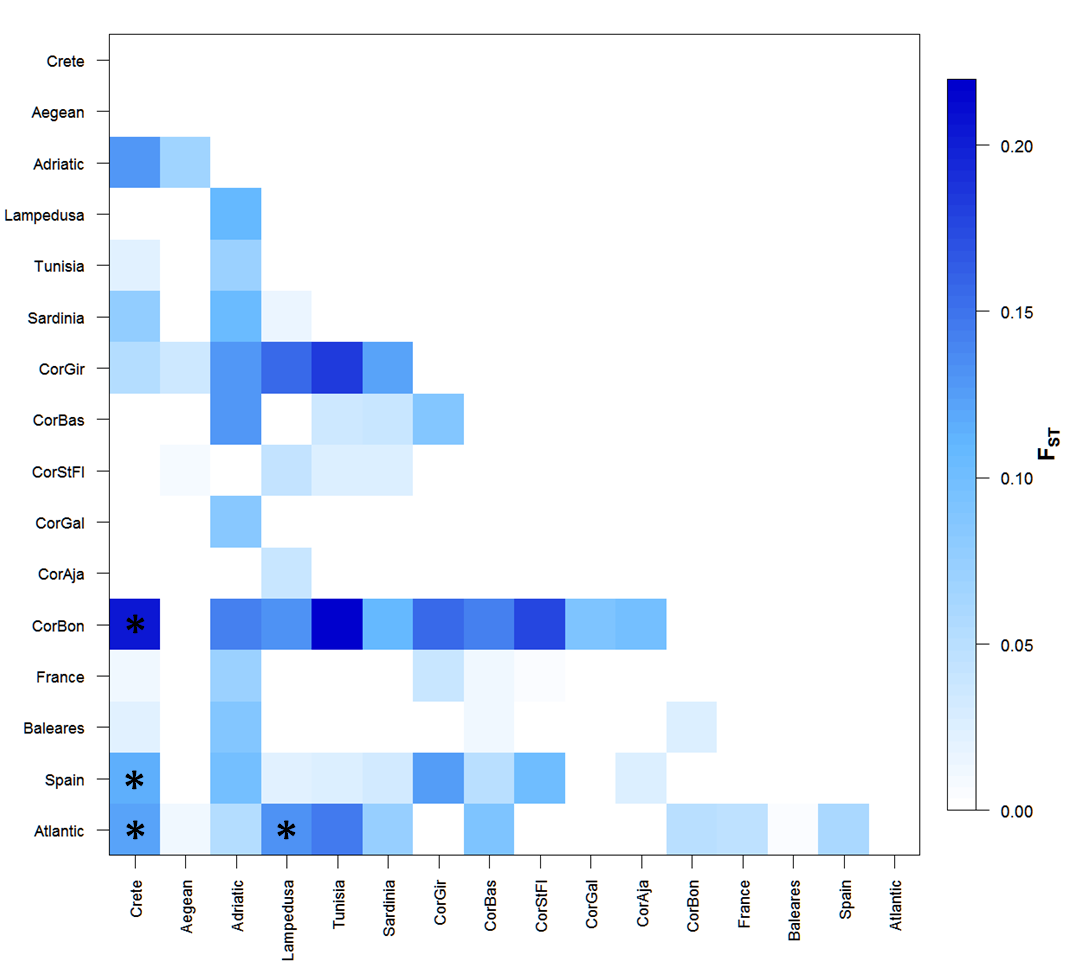

Supplement: S1 Fig — Asterisk indicates P≤ 0.05 in terms of statistical significance. (TIFF) [file pone.0203866.s006.tiff]

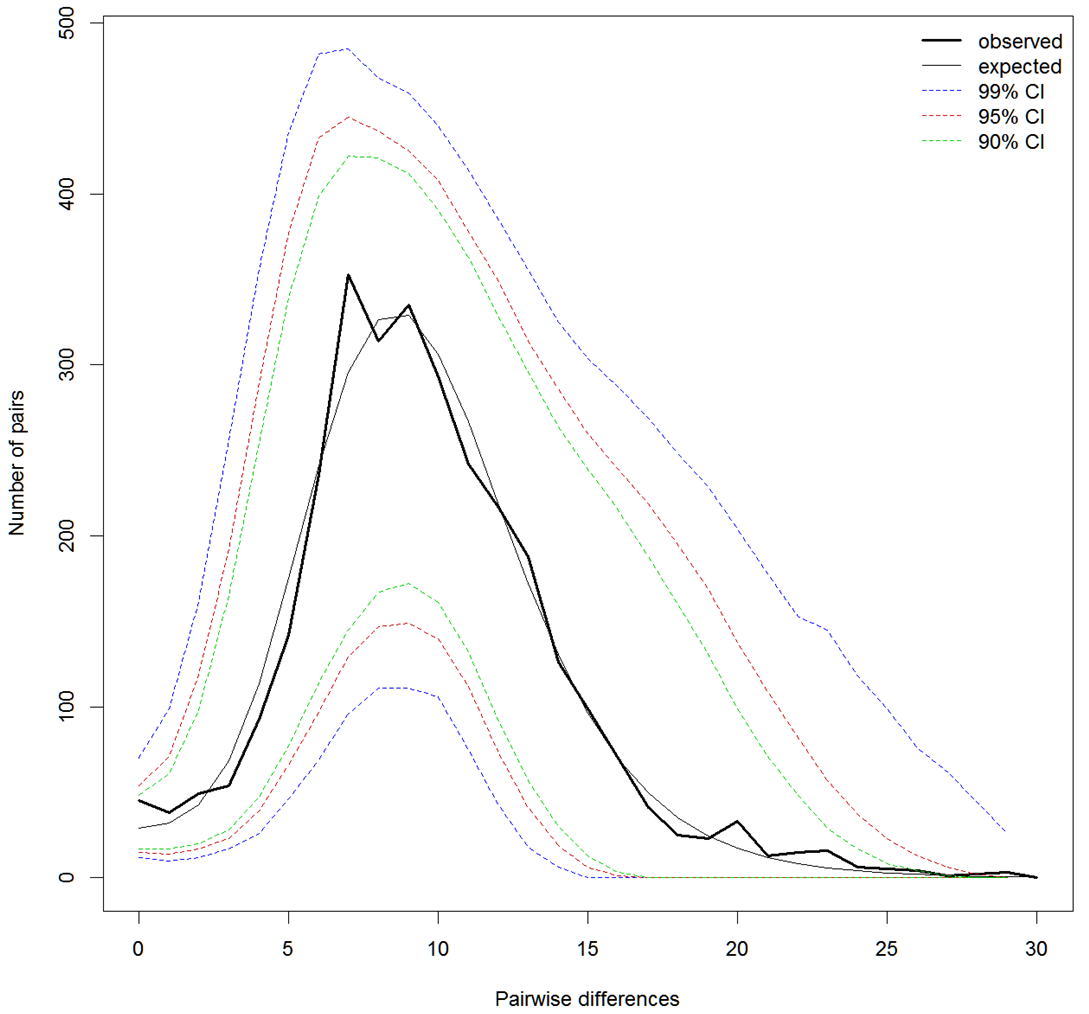

Supplement: S2 Fig — (TIFF) [file pone.0203866.s007.tiff]

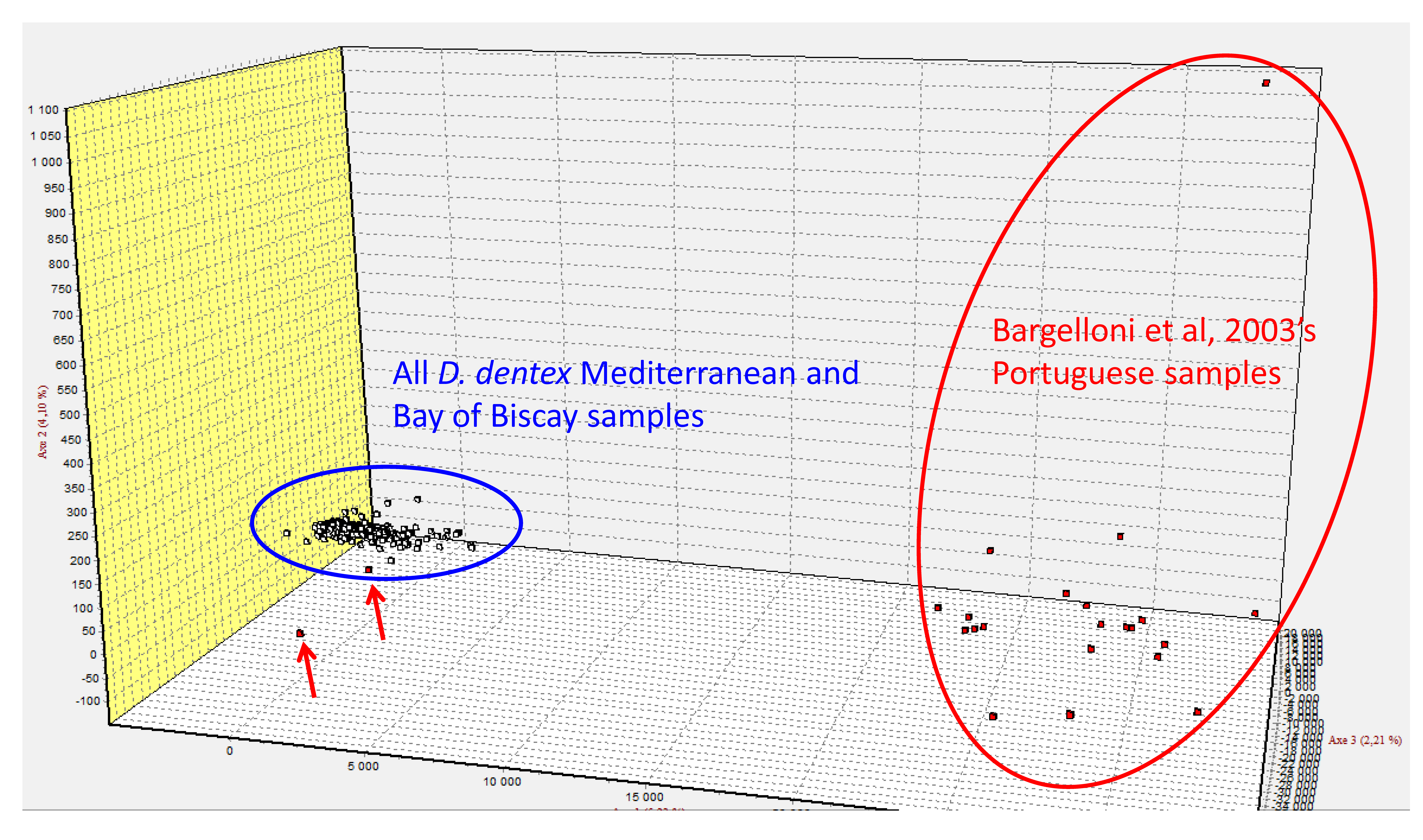

Supplement: S3 Fig — In the blue envelope are the very homogeneous samples of Mediterranean and Bay of Biscay origin. The red envelope gathers the Bargelloni et al. 2003 Portugal samples. Clearly, Bargelloni's Portuguese fish are heterogeneous (at least three clouds) and do not belong to D. dentex (except two individuals: see red arrows). Consequently, the Bay of Biscay samples are common dentex similar to those of Mediterranean populations. (TIFF) [file pone.0203866.s008.tiff]

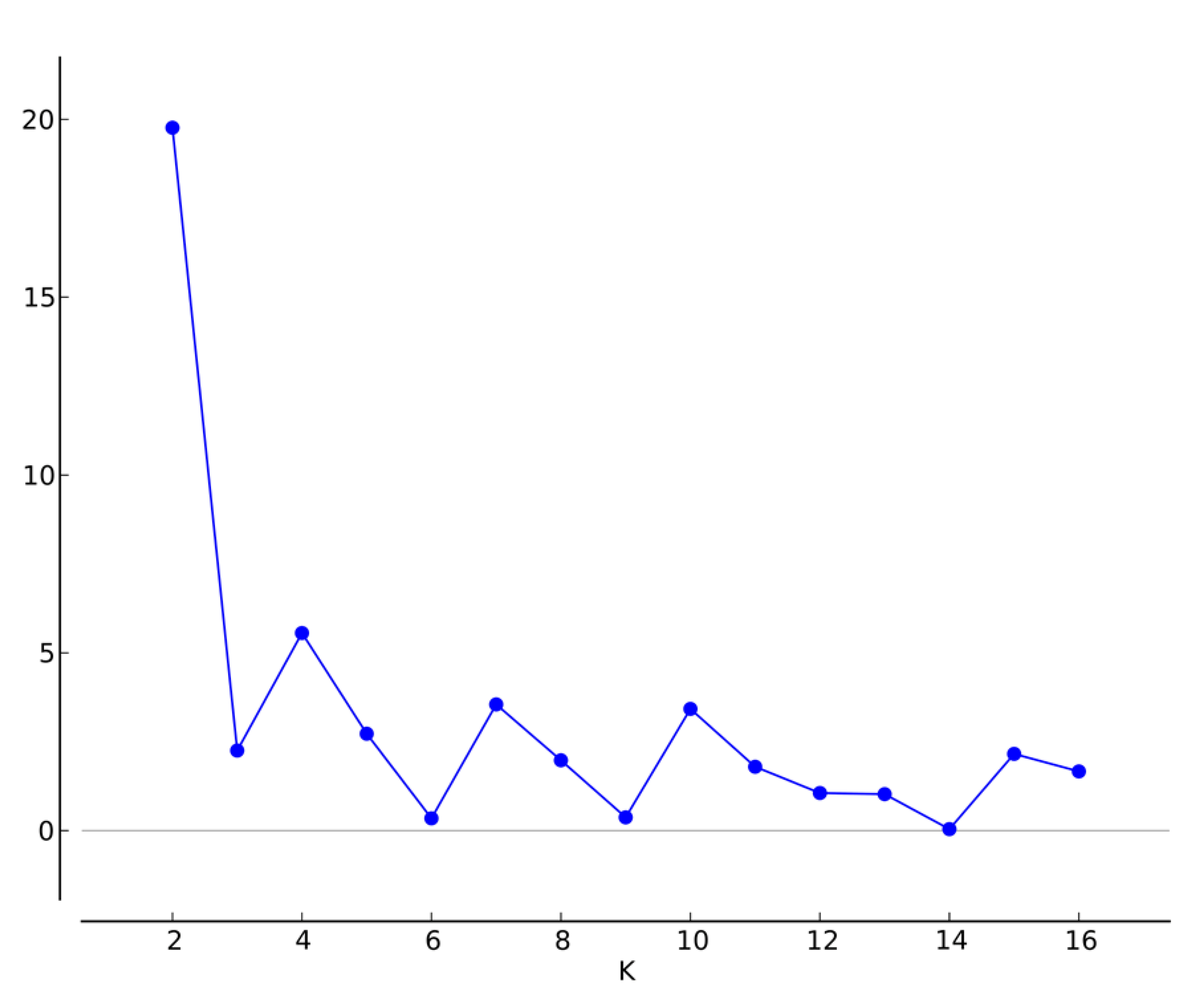

Supplement: S4 Fig — (TIFF) [file pone.0203866.s009.tiff]

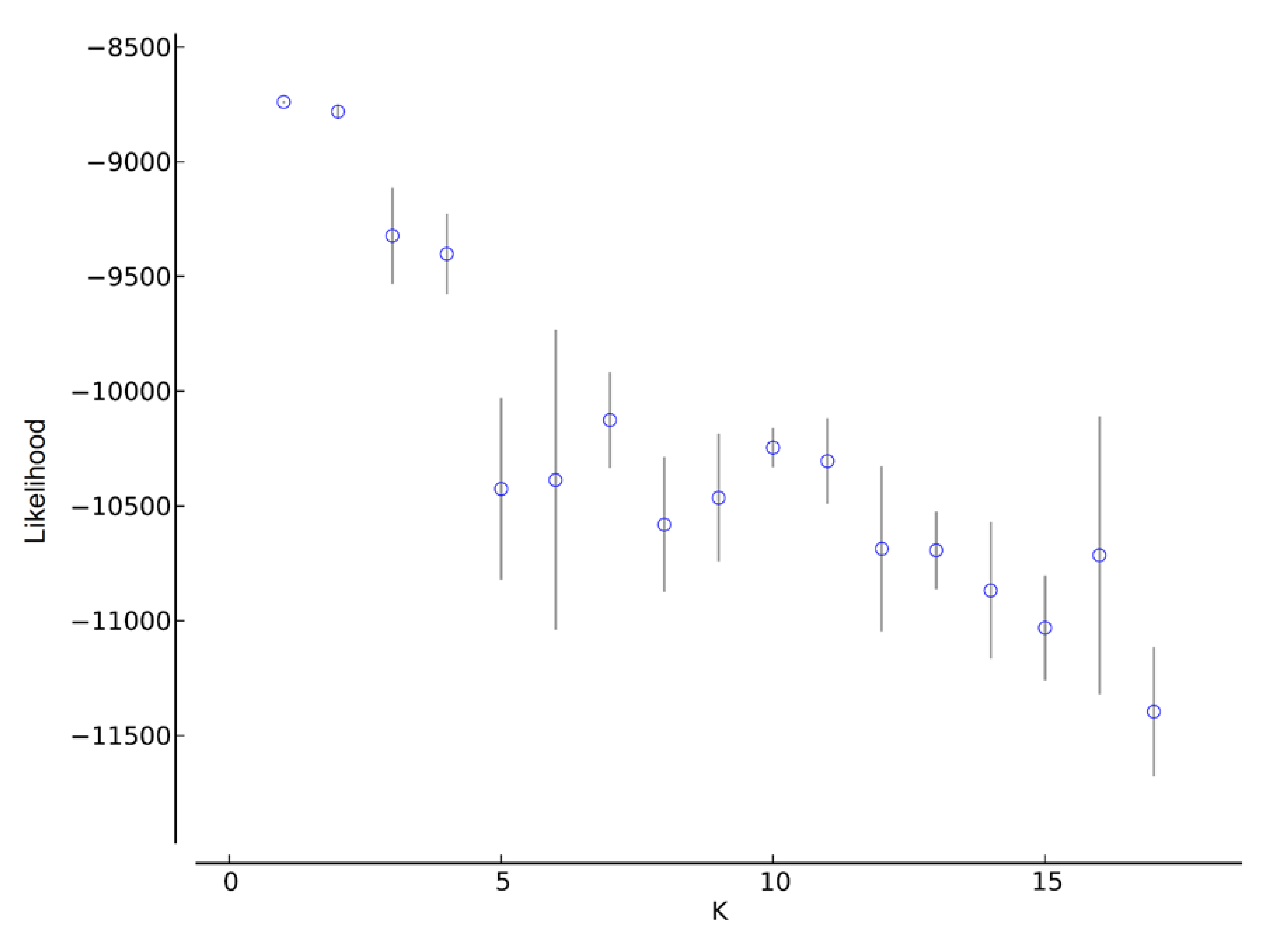

Supplement: S5 Fig — Standard error bars are also indicated. (TIFF) [file pone.0203866.s010.tiff]
